# A Systematic Review of Anchored and Unanchored EB-FRP Systems for Tension Strengthening of Concrete Structures

**DOI:** 10.3390/polym18131598

**Published:** 2026-06-26

**Authors:** Junrui Zhang, Enrique del Rey Castillo, Mohammad Sadegh Salimian Rizi, Tingting Yu

**Affiliations:** Department of Civil and Environmental Engineering, University of Auckland, Auckland 1023, New Zealand

**Keywords:** EB-FRP, FRP anchors, debonding, reinforced concrete, systematic review

## Abstract

Externally bonded fiber-reinforced polymer (EB-FRP) systems have been extensively investigated for tension strengthening concrete structures. Interpretation of the available evidence remains challenging because experimental methods, specimen scales, material systems, anchorage configurations, and reporting practices vary substantially across the literature. This systematic review synthesized 174 peer-reviewed studies published between 1994 and 2026, comprising 3908 experimental test results and 42 analytical formulations addressing unanchored and anchored EB-FRP systems. Review findings showed that bond performance in unanchored systems is governed primarily by FRP stiffness, bond geometry, concrete properties, adhesive behavior, surface preparation, and environmental exposure. These parameters influence bond capacity, debonding strain, effective bond length, and failure mode. Anchored configurations consistently enhanced force transfer, delayed premature debonding, and improved load-carrying capacity relative to unanchored systems. Unanchored systems dominated the available evidence base with 3162 test results, whereas only 96 multi-anchor system tests were identified, highlighting limited understanding of anchor interaction and load redistribution mechanisms. CFRP represented the dominant material system, while substantially fewer studies investigated GFRP, BFRP, and AFRP systems. Existing strength models generally captured specific failure mechanisms within their calibration ranges but demonstrated limited transferability across different geometries, loading conditions, anchorage configurations, and environmental conditions. Limited evidence remains available for scale transfer, durability degradation, anchor strip interaction, and multi-anchor load sharing under field-representative conditions. Future research should focus on standardized benchmarking procedures, large-scale validation programs, durability-informed design approaches, experimentally validated numerical modeling, and unified design provisions for EB-FRP strengthening systems.

## 1. Introduction

**Figure 1:**
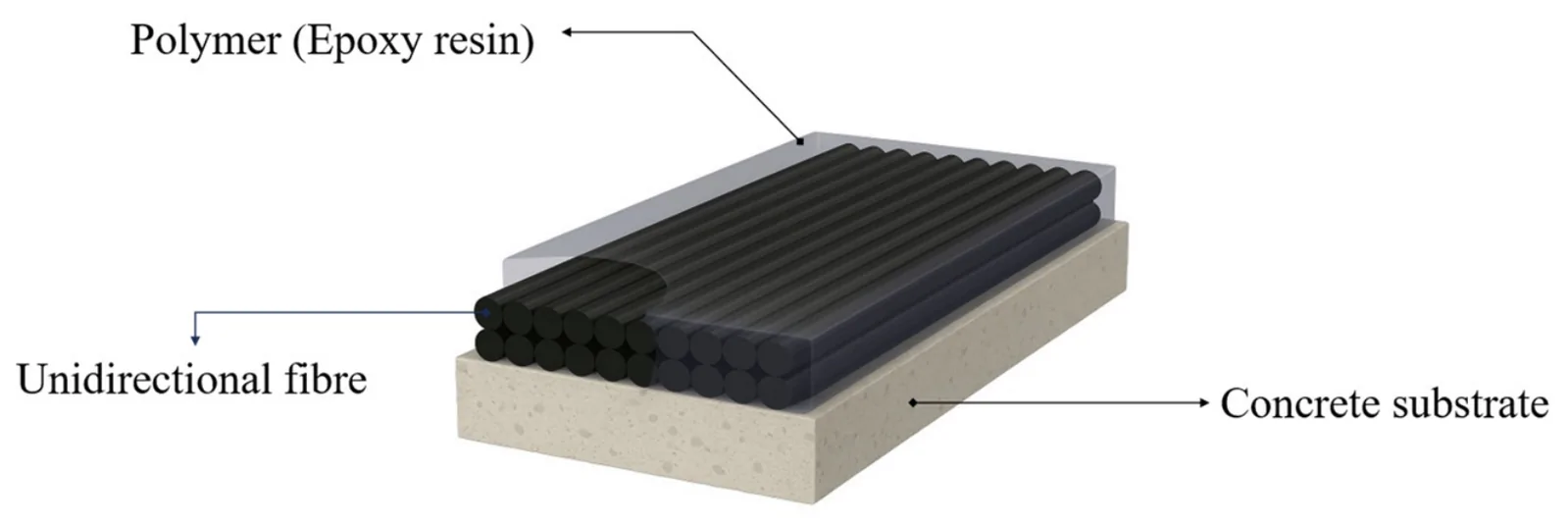
Schematic of a typical unidirectional FRP composite bonded to a concrete surface using epoxy resin.

Externally bonded fiber-reinforced polymer (EB-FRP) systems have become popular in the rehabilitation of deteriorated concrete infrastructure due to their high tensile strength, corrosion resistance, and ease of installation without adding significant weight [1,2]. These systems offer practical advantages for flexural strengthening of beams [3,4,5], axial confinement of columns [6,7,8], and tensile enhancement of slabs [9,10,11] and shear walls [12,13,14]. EB-FRP systems are also widely applied in seismic and blast retrofitting, where rapid installation and minimal section enlargement are critical [15,16]. A typical EB-FRP configuration uses unidirectional fibers bonded to concrete substrates with epoxy resin, as shown in Figure 1. Load is transferred through shear stress along the adhesive–concrete interface, where stiffness mismatch between the materials concentrates stress near the loaded end of the sheet, often triggering premature debonding failures [17,18]. Debonding failures typically occur in three principal forms: cohesive failure within the concrete substrate, cohesive failure within the adhesive layer, and adhesive failure at the concrete, adhesive, or adhesive–FRP interface [19,20]. Among these, failure within a thin surface layer of concrete, commonly referred to as concrete cover separation, is the most frequently observed and often governs overall system performance. Adhesive and interfacial failures are less prevalent but can become critical when surface preparation, adhesive quality, or bond continuity is inadequate. All three modes are brittle and result in limited energy dissipation, which reduces the effective utilization of FRP tensile strength. A clear understanding of the conditions governing each mode is essential for effective FRP system design.

Research has established that EB-FRP system performance is further influenced by parameters such as strip geometry and the mechanical properties of the FRP and concrete materials [11,21]. Insufficient control of these variables can diminish bond capacity and cause premature failure. Environmental conditions, including elevated temperature and moisture exposure, also accelerate degradation, particularly in adhesive-dependent systems [22,23]. These effects are especially problematic in high-performance applications involving stiff or thick FRP laminates, which intensify interfacial stress gradients and reduce resistance to debonding. Several test methods have been developed to assess EB-FRP bond performance under tension. These include direct tension tests [24], tension-face flexural tests (e.g., three- or four-point bending) [25,26], and single or double lap-shear tests [27,28]. Each method imposes distinct interfacial stress states that influence bond characteristics and failure mechanisms. A systematic understanding of how test setup, material processing, and surface preparation affect test results is essential to ensure reliable performance evaluation.

**Figure 2:**
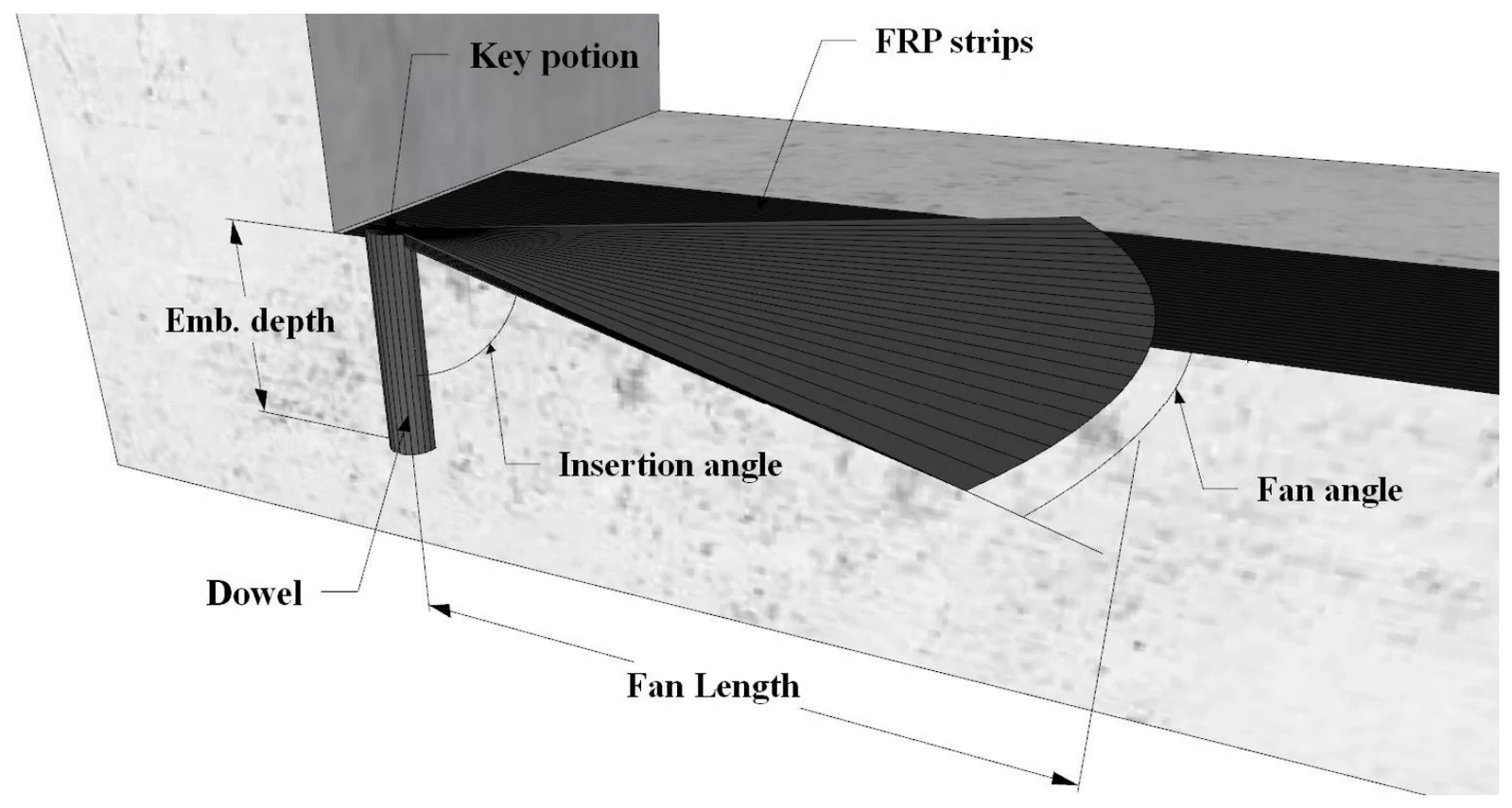
Schematic of the geometry and layout of a fan-shaped fiber anchor in an EB-FRP system.

Fiber anchors have been introduced to enhance bond capacity by providing mechanical confinement and delaying debonding [29,30,31]. Among various configurations such as bow-tie [32], circular [33], or through-anchors [34], the fan-shaped anchor remains the most widely adopted due to its proven effectiveness and ease of fabrication [35]. These anchors are commonly constructed from the same FRP material and are embedded into the concrete to form a dowel/fan system. Figure 2 shows the typical layout of the dowel/fan anchor system embedded in concrete. The fan portion distributes load to surrounding concrete, while the dowel provides axial restraint. Anchor efficiency is governed by embedment depth, insertion angle, fan width, and spatial arrangement. Many existing studies have evaluated shallow and small-scale anchors, constrained by the physical limitations of retrofit applications [29,32,36,37,38,39,40,41,42]. For instance, slab toppings often range from 50 to 75 mm in thickness, restricting embedment depth [43]. Anchor performance is typically evaluated through direct tension (pullout) [38] or lap-shear tests [43]. Improper test configuration or design detailing can lead to undesired failure modes such as anchor pullout or concrete cone detachment, rather than the more predictable fiber rupture at the fan–concrete interface [44].

**Figure 3:**
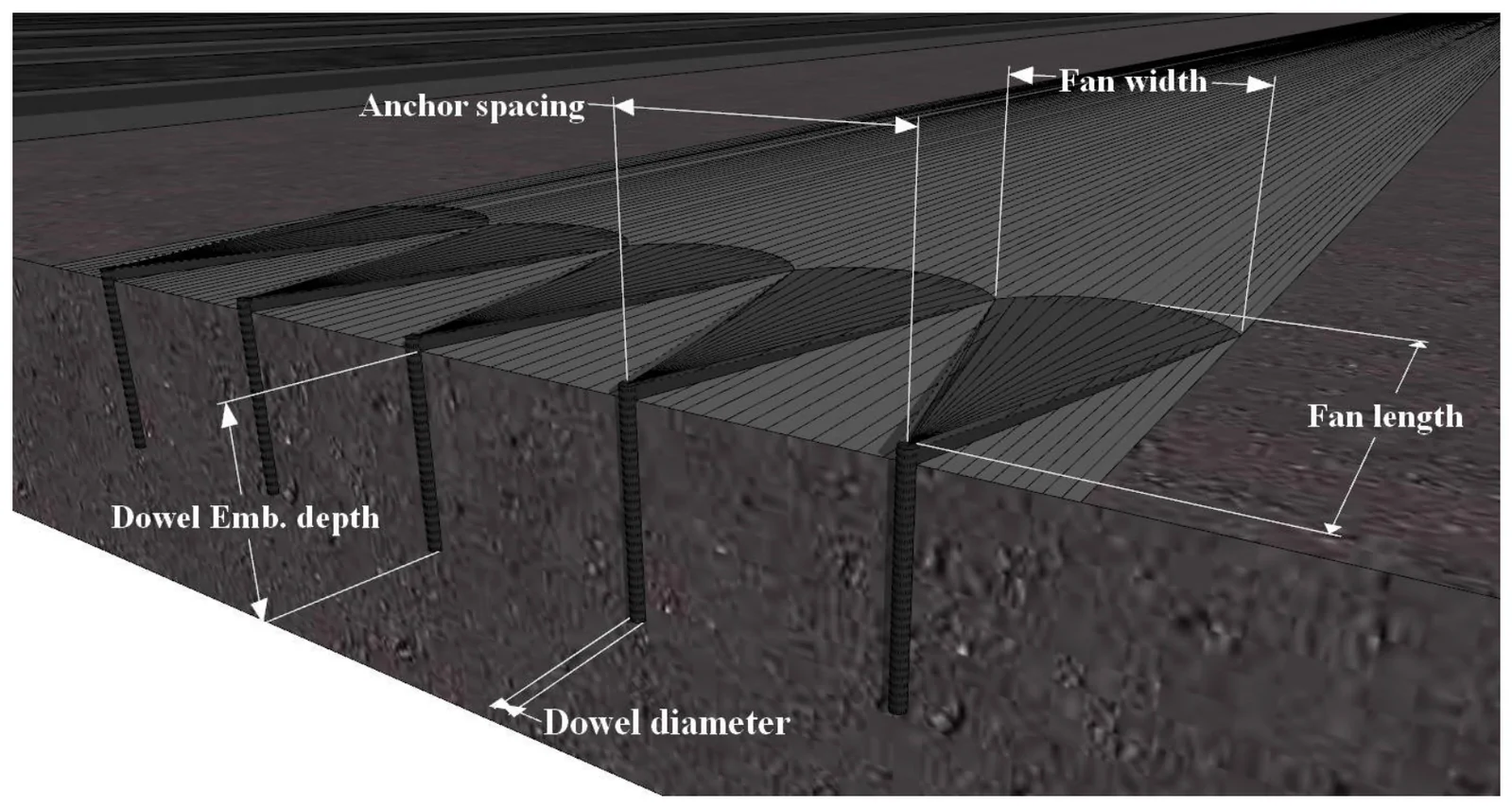
Schematic of a multi-anchored EB-FRP system.

Multi-anchor systems introduce additional complexity by altering load paths and reducing the effective bond length per anchor. Figure 3 shows a multi-anchored EB-FRP system used in concrete diaphragm retrofitting. The interaction between anchor fans creates stress redistribution effects that remain poorly characterized. Parameters such as FRP stiffness, anchor density, sheet overlap, and concrete modulus collectively influence strain gradients and peak interfacial stress [45,46]. Current design codes remain inadequate for guiding EB-FRP systems that incorporate anchors. ACI 440.2R-17 [47] adopts simplified assumptions that omit anchorage mechanics and fail to reflect the nonlinear interfacial behavior observed in experiments [14]. Similarly, the CNR-DT 200 [48] guideline proposes empirical bond strength equations but lacks provisions for anchored systems [20]. The fib Bulletin 90 [49] extends the fib Bulletin 14 model database but retains a primary focus on unanchored, small-scale configurations. Similarly, existing fracture mechanics-based theoretical models, primarily developed from tests on thin and short FRP strips and small, shallow anchors under bending or lap-shear configurations [50], lack general applicability to realistic field-scale conditions. Most available predictive models for isolated anchored systems are empirical and calibrated to narrow parameter ranges, limiting their usefulness for broader structural applications. These limitations highlight the need for a systematic review to consolidate findings, strengthen modeling generalizability, and support more practical and transferable design approaches.

Data acquisition systems (DAQs) play a critical role in understanding EB-FRP anchorage behavior. Conventional instrumentation such as strain gauges and LVDTs captures only localized responses and fails to resolve distributed slip or crack development [51,52,53]. Advanced systems such as digital image correlation (DIC) and embedded fiber optic sensors enable full-field strain visualization and high-resolution tracking [54,55,56]. Despite their advantages, these tools remain underutilized due to cost, setup complexity, and limited access in large-scale testing. Current studies often employ hybrid data acquisition approaches to combine the strengths of multiple systems, but a lack of standardization and limited literature coverage leave open questions regarding optimal configurations [57,58,59,60]. Further investigation is required to assess how integrated DAQ strategies contribute to performance evaluation.

This systematic review addresses these limitations by synthesizing and categorizing experimental studies using a PRISMA-guided approach [61,62,63,64]. Key research questions include the influence of FRP anchors on interfacial behavior, the generalizability of existing bond–slip models, and the limitations of current design standards. A Complexity of Investigation (COI) classification is applied to benchmark experimental rigor, instrument precision, and model sophistication across the published literature. The goal is to establish a foundational database that enables improved analytical models, informed design guidelines, and optimized anchorage configurations for EB-FRP systems under tension. The research gap addressed by this review is therefore not only the absence of additional test data, but the lack of a traceable, metric-based synthesis that connects test configuration, FRP material, bond geometry, environmental exposure, anchorage configuration, and model validation to comparable performance outcomes. In particular, the literature still lacks normalized evidence for scale transfer, durability-sensitive bond degradation, anchor–strip interaction, multi-anchor load sharing, and the accuracy limits of empirical strength models.

## 2. Methodology

### 2.1. PRISMA-Guided Review Framework

**Figure 4:**
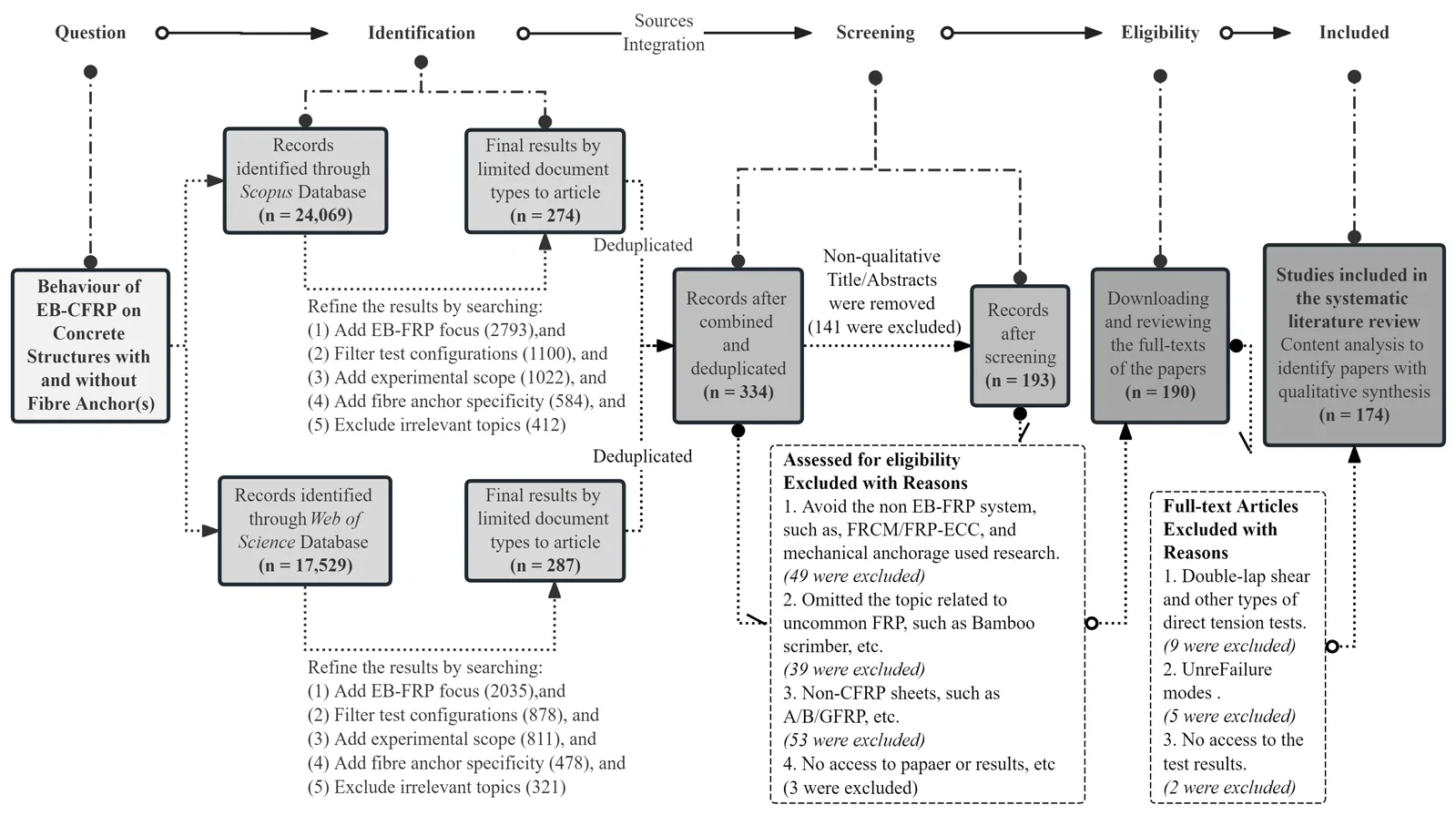
The PRISMA flowchart.

This systematic literature review synthesizes and evaluates experimental research on EB-FRP systems used to strengthen concrete structures in tension. The review focuses on parameters influencing interfacial performance and experimental test configurations. A systematic review approach was adopted to reduce the subjectivity often found in narrative reviews and to improve reproducibility in study identification, coding, and synthesis. Through this approach, the review maps research trends and links database outputs to ongoing developments in EB-FRP system behavior. The methodology follows the PRISMA (Preferred Reporting Items for Systematic Reviews and Meta-Analyses) 2020 checklist [60,61,62,63], which supports structured evidence identification and synthesis. Five main stages guided the review process: (1) framing research questions, (2) identifying relevant studies, (3) screening for quality and relevance, (4) conducting full-text eligibility checks, and (5) summarizing and interpreting findings. These stages are outlined in Figure 4 and elaborated in the following subsections.

### 2.2. Framing Research Questions

To guide the review, three primary research questions (RQs) were formulated to evaluate the performance of EB-FRP systems for concrete strengthening, with and without the use of fiber anchors:

RQ1. How do non-anchored EB-FRP systems perform in strengthening concrete under axial tension, measured by ultimate bond capacity, bond strength or capacity per bonded width, debonding strain or strain profile, effective bond length, fracture energy or load–slip response where reported, and observed failure mode?

RQ2. How do fiber anchors influence bond behavior and load-transfer efficiency in EB-FRP systems?

RQ3. What are the current limitations and future research needs for EB-FRP systems, both with and without fiber anchors?

**Figure 5:**
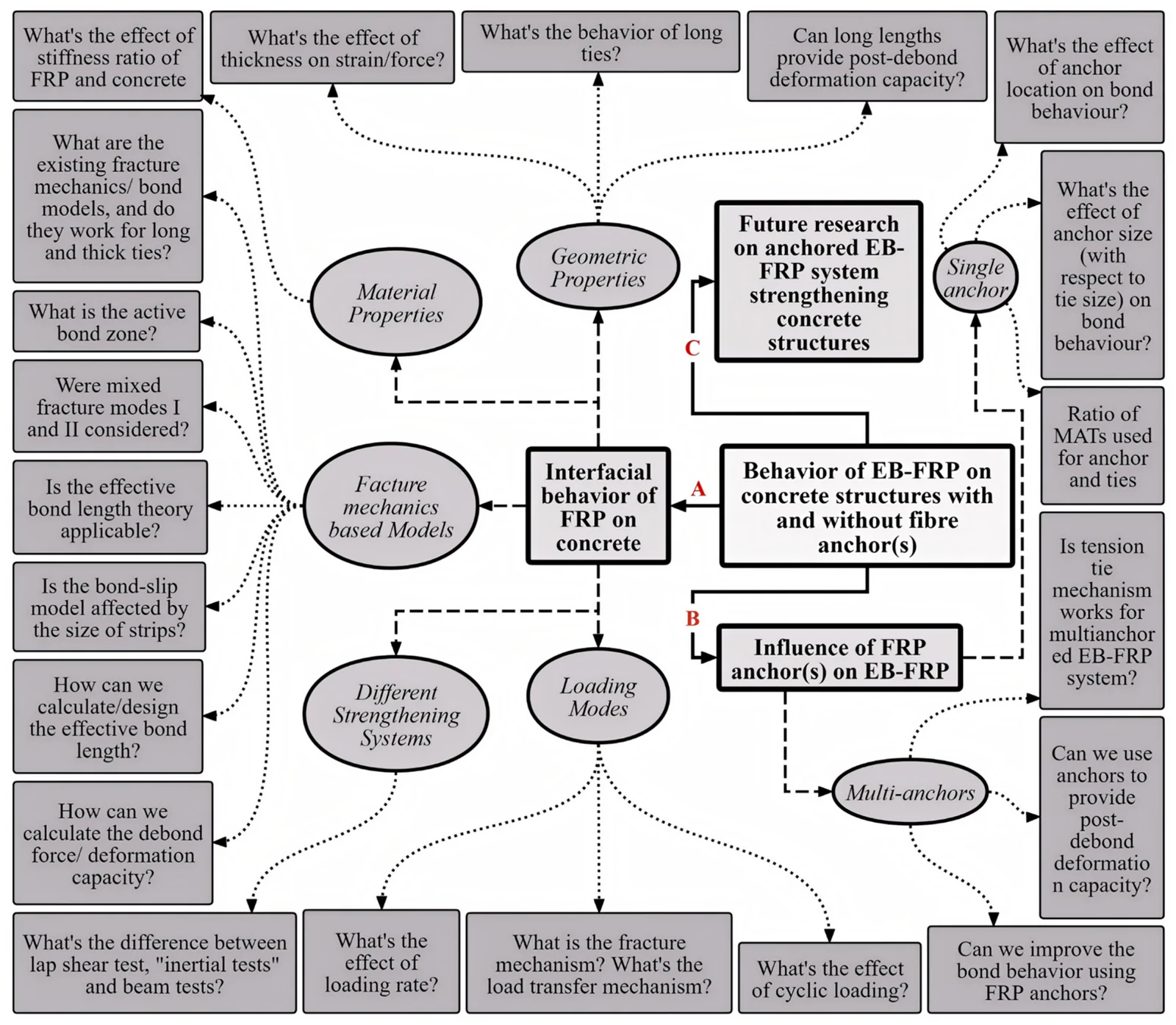
Mind map linking the research questions to major EB-FRP review themes. The red labels A, B, and C denote the three principal research pathways considered in this review: (A) interfacial behavior of FRP on concrete without anchors, (B) influence of FRP anchor(s) on EB-FRP systems, and (C) future research needs and knowledge gaps in end-anchored FRP strengthening of concrete structures.

The research questions define the scope of the review by focusing on the performance of bonded FRP strips, the contribution of FRP anchors to interfacial performance, and areas where further research is needed. Figure 5 shows a mind map that links related questions to major themes in the literature, including fracture mechanics, geometric effects, anchor layout, and bond failure mechanisms. For traceability, RQ1 was evaluated through extracted performance indicators rather than a single generic performance label. Direct comparisons used reported ultimate load, normalized capacity per FRP width where sufficient geometry was available, FRP strain or debonding strain where measured, effective bond length, bond–slip or load–slip response, fracture energy, and failure mode. RQ2 used the same base indicators and added anchor geometry, embedment depth, fan angle, insertion angle, dowel diameter, anchor spacing, and single- versus multi-anchor configuration. RQ3 was derived from evidence gaps revealed by these coded variables, especially missing large-scale, durability, and model-validation data.

The literature search used two academic databases, Scopus and Web of Science. Identical search strategies were applied to both databases using structured keyword combinations tailored to the research topic. This dual-database approach ensured comprehensive coverage and helped identify studies across different publishers. In addition to database searches, reference lists of selected papers were reviewed to identify further relevant studies through citation tracking.

### 2.3. Literature Search Strategy

The literature search targeted experimental studies on EB-FRP systems for concrete strengthening, with and without FRP anchors. Scopus and Web of Science were selected as the primary and secondary databases, respectively. Identical search strategies were applied across both platforms to ensure consistency. The search included all relevant journal articles published up to 17 February 2026, without restrictions on earlier publication dates. Only journals indexed in the SCImago Journal Rank (SJR) database were considered. The SJR system provides broader coverage than other ranking indicators, including Journal Impact Factor (JIF), Eigenfactor Score (ES), and H5 [65]. A total of 302 civil and structural engineering journals were included, consisting of 80 Q1, 80 Q2, 75 Q3, and 67 Q4 journals. Strict eligibility criteria were applied to maintain the quality and relevance of the included studies. Each article was reviewed based on its title, abstract, and methodology. Studies were retained only if they met all of the following conditions:

1. Published in any year, up to 17 February 2026.
2. Focused on EB-FRP strengthening of concrete, with and without FRP anchors, as indicated in the title, abstract, or keywords.
3. Reported experimental work based on direct tension or lap-shear tests; studies using numerical models, simulations, or finite element analysis (FEM) were excluded.
4. Written in English and published in final form as peer-reviewed journal articles.

The search process was designed around the three primary research questions. Keywords were selected in advance to reflect core themes of EB-FRP behavior, interfacial bonding, and anchorage performance. In the first stage, broad search terms such as “FRP and concrete” and “Fiber/Fiber Reinforced Polymer/Plastics and Concrete” were used to ensure topic coverage. This initial stage yielded 24,069 results from Scopus and 17,529 from Web of Science, reflecting the growing interest in FRP systems over recent decades. Terms such as “CFRP” or “carbon fiber” were intentionally excluded from the early stages to avoid excluding studies involving GFRP or BFRP that may not use those specific terms. The remaining stages applied Boolean operators (AND, OR, and NOT) to progressively refine the results, targeting experimental work on EB-FRP under tension or lap-shear loading and eliminating irrelevant topics. Scopus was prioritized due to its broader coverage, which includes approximately 99.11% of the publications indexed by Web of Science [66]. The full breakdown of search terms and filtering stages is shown in Table 1, which documents the sensitivity of the search result to each keyword-refinement stage. The table should be read from top to bottom: broad FRP–concrete terms establish coverage, EB-FRP and bond-behavior terms increase specificity, material and anchorage terms retain relevant CFRP/GFRP/BFRP/AFRP and anchor studies, and exclusion terms remove numerical, machine learning, non-EBR, and non-bond-focused records before deduplication. This staged reporting makes the effect of each keyword family transparent without changing the final eligibility criteria.

Quantitative examples were extracted retrospectively from the final included studies after completion of the screening process to improve transparency regarding keyword sensitivity. Table 2 preserves the original coverage explanations and residual limitations for each alternative term family. The additional information does not alter the PRISMA workflow, the eligibility criteria, or the screening process. The final study selection process and the number of included studies are presented in the following section. Percentages reported in Table 2 were calculated using the final set of 174 included studies.

In the second search stage, the keywords “externally bonded” and “EB-FRP” were applied to isolate studies focused specifically on the externally bonded reinforcement (EBR) method. This step aimed to eliminate studies on alternative techniques such as near-surface mounted (NSM), grooving methods (GM), or hybrid bonding (HB) systems. Subsequent stages incorporated keywords such as “tension test” and “lap-shear test” to capture experimental configurations relevant to axial and shear performance. During the fourth and fifth stages, additional filters were introduced to capture studies investigating FRP anchor effects and interfacial bond behavior. These terms aligned directly with the review’s first two research questions. Notably, in Stage 5, the Boolean operator used for Web of Science was changed from “OR” to “AND” when combining keywords. This adjustment was necessary due to differences in search engine algorithms, which produced an excessive number of unrelated results when “OR” was used in Web of Science. Scopus retained the original “OR” logic, while Web of Science served a supplementary role in this round. Stage 6 excluded irrelevant studies involving numerical analysis, finite element modeling, machine learning, and algorithm-based approaches. These were filtered using the “NOT” operator applied to keywords such as “finite element,” “simulation,” “machine learning,” and related terms. No restrictions were imposed on publication year or journal tier. The final filter in Stage 7 limited the dataset to English-language peer-reviewed journal articles or review papers. Other types of publications, including conference proceedings, book chapters, and editorials, were excluded. The complete Boolean search strings used in the final round are shown below:

Scopus search string:

TITLE-ABS-KEY ((“FRP” OR “FIBER REINFORCED POLYMER”) AND (CONCRETE OR “REINFORCED CONCRETE” OR RC) AND (“EXTERNALLY BONDED” OR EB-FRP OR “BONDED REINFORCEMENT”) AND (“PULLOUT TEST” OR “LAP SHEAR” OR “DIRECT TENSION” OR “BOND-SLIP” OR “LOAD-SLIP” OR DEBOND*) AND (EXPERIMENT* OR TEST* OR “BOND BEHAVIOR” OR “INTERFACIAL BEHAVIOR” OR “FAILURE MODE” OR “BOND STRENGTH”) AND (“CFRP” OR “CARBON FIBER” OR “STRAIN PROFILE” OR DIC OR “FAN ANGLE” OR “EMBEDMENT DEPTH” OR “FRP ANCHOR*” OR “SPIKE ANCHOR*” OR “FRP TIE*” OR “MECHANICAL ANCHORAGE”)) AND NOT TITLE-ABS-KEY (NSM OR “NEAR SURFACE MOUNTED” OR “MACHINE LEARN*” OR “DEEP LEARN*” OR ALGORITHM* OR SIMULAT* OR “FINITE ELE*”) AND (LIMIT-TO (DOCTYPE,”ar”) OR LIMIT-TO (DOCTYPE,”re”)) AND (LIMIT-TO (LANGUAGE, “English”)).

Web of Science search string:

TS = ((“FRP” OR “FIBER REINFORCED POLYMER”) AND (CONCRETE OR “REINFORCED CONCRETE” OR RC) AND (“EXTERNALLY BONDED” OR EB-FRP OR “BONDED REINFORCEMENT”) AND (“PULLOUT TEST” OR “LAP SHEAR” OR “DIRECT TENSION” OR “BOND-SLIP” OR “LOAD-SLIP” OR DEBOND*) AND (EXPERIMENT* OR TEST* OR “BOND BEHAVIOR” OR “INTERFACIAL BEHAVIOR” OR “FAILURE MODE” OR “BOND STRENGTH”) AND (“CFRP” OR “CARBON FIBER” OR “STRAIN PROFILE” OR DIC OR “FAN ANGLE” OR “EMBEDMENT DEPTH” OR “FRP ANCHOR*” OR “SPIKE ANCHOR*” OR “FRP TIE*”)) NOT TS = (NSM OR “NEAR SURFACE MOUNTED” OR “MACHINE LEARN*” OR “DEEP LEARN*” OR ALGORITHM* OR SIMULAT* OR “FINITE ELE*”) AND DT = (“ARTICLE” OR “REVIEW”) AND LA = (“ENGLISH”).

The search was limited to titles, abstracts, and keywords. After deduplication across both databases, a total of 334 unique journal articles remained for screening and further analysis.

### 2.4. Eligibility Criteria and Screening Process

After finalizing the initial search, the screening process began by reviewing each article’s title and abstract to determine its relevance based on predefined inclusion and exclusion criteria. This process followed a two-round approach to ensure consistency and minimize omission, and the inclusion and exclusion criteria are summarized in Table 3.

Each study was independently reviewed at least twice by the authors. The first round focused on screening titles and keywords, while the second round evaluated abstracts. A shared decision protocol ensured consistency. Any disagreements were resolved through discussion and mutual agreement. Table 4 shows the number of papers excluded at each round along with the reasons.

Numerical and FEM studies were excluded from the primary evidence base because this review compares experimental performance metrics and failure observations that can be traced to physical specimens. Simulation studies are valuable for mechanism exploration and parameter studies, but they depend on assumptions about interface laws, fracture energy, mesh strategy, and calibration data; including them in the same quantitative pool as tests would blur measured outcomes with model outputs. Numerical work is therefore discussed only where it supports interpretation of experimental models, and its systematic synthesis is identified as a separate future research need.

After the first-round screening, 247 articles remained. In the second round, 54 additional articles were removed, leaving 193. Following full-text checks and final consensus between reviewers, 19 more articles were excluded. A total of 174 studies remained for full analysis.

### 2.5. Data Extraction and Thematic Coding

After applying the eligibility criteria, a total of 174 articles were retained for full-text analysis. The process leading to this selection was outlined in Figure 4. Although the initial search identified 41,598 articles across Scopus and Web of Science, most were excluded through keyword refinement and eligibility screening. Articles were eliminated if they used unrelated materials (e.g., bamboo fiber, textile-reinforced mortar, and engineered cementitious composites) or if full-text access was not available [66]. To extract structured information from the included studies, this review adopted a thematic template analysis approach [67]. This method offers flexibility in analyzing qualitative text while being grounded in predefined research questions. Unlike fixed-form coding schemes, the template approach allows iterative adjustment of themes as new patterns emerge during review. Compared to alternative methods like analytical induction [68], interpretive phenomenological analysis [69], discursive psychology [70], and grounded theory [71], template analysis offers improved structure and adaptability for large-scale technical reviews. The PRISMA 2020 checklist was used as a guiding protocol to ensure transparency and reproducibility in the data extraction process [62]. A modified version of the checklist was created for this review (Table 5); items related to formal meta-analysis were not applicable, while the engineering quality controls used for screening, extraction, comparability, and limitations are reported in the methods and synthesis sections. After refining the checklist, 17 applicable items were retained to structure the review process.

Following the modified PRISMA checklist, a thematic coding template was developed and applied across all 174 studies (Table 6). This template included both general metadata and research-specific content. Each paper was categorized according to six main analytical domains.

All coding was done manually through full-text reading, using a spreadsheet-based matrix to maintain consistency and traceability. Studies were grouped according to theme, research question relevance, and contribution type. This structured classification provided the basis for the analytical synthesis that follows in subsequent sections. Quantitative synthesis and normalization were performed at the test-result level where the source papers reported sufficient information. Extracted values were first retained in their reported units, then grouped by COI level, test method, FRP type, bond geometry, loading configuration, and failure mode. Direct pooling was avoided when studies used incompatible boundary conditions or missing geometry. When geometry was available, comparisons emphasized normalized or scale-aware indicators, including capacity per bonded FRP width, FRP stiffness ratio, bond length relative to effective bond length, debonding strain, strain profile shape, load–slip or bond–slip response, fracture energy, normalized anchor area, embedment-depth-to-dowel-diameter ratio, and anchor spacing or count. Where studies reported only ultimate load or qualitative failure observations, those data were used for stratified trend interpretation rather than direct numerical ranking. The normalization framework, comparison criteria, and links between the research questions and extracted evidence are summarized in Table 7.

### 2.6. Complexity of Investigation (COI) Assessment

All 174 included articles were evaluated using a structured thematic template and categorized based on their contribution to the core research questions. To systematically assess the depth, workload, and execution difficulty of each experimental study, a Complexity of Investigation (COI) matrix was developed. This matrix classified studies into three levels, CoI-A, CoI-B, and CoI-C, based on four evaluation aspects: (1) Objectives of the experimental program; (2) data acquisition methods; (3) theoretical contribution or model development; and (4) failure mode characterization and interpretation. The classification criteria and descriptions for each COI level are shown in Table 8.

Each study was assigned a COI level based on its alignment with the four evaluation dimensions. COI-A denotes baseline investigations focused on unanchored EB-FRP systems tested under monotonic direct tension or lap-shear conditions. These studies employed minimal instrumentation and lacked model development or validation. COI-B represents intermediate studies involving FRP-anchored systems applied to concrete, typically with single-anchor configurations. These investigations included limited parameter variation and some use of empirical models or comparative analyses. COI-C reflects advanced studies that combined FRP sheets with multiple anchors, examined system-level behavior under variable conditions, and incorporated detailed instrumentation (e.g., strain field mapping and digital image correlation). These studies often validated or modified analytical models and provided comprehensive failure mode analysis. The COI classification enabled systematic comparison across studies and helped evaluate the experimental and analytical depth contributing to the review’s synthesis. Since some studies addressed more than one objective, thematic categories were treated as analytical labels rather than mutually exclusive bins. Each paper received a primary label based on its dominant experimental purpose and secondary labels for additional variables or model contributions. For example, a study on anchored lap-shear behavior that also proposed a strength equation was counted under the appropriate anchor-configuration objective and also considered in the model-validation discussion. This approach explains why sub-objective classifications can exceed the number of unique studies while the PRISMA corpus remains fixed at 174 papers.

### 2.7. Interpretation and Synthesis

Assessing the overall complexity of an experimental study using only a single COI score can be misleading. Many papers contain multiple test programs that vary in detail, instrumentation, or modeling depth. For instance, a study classified as COI-A might include a basic FRP sheet test and also present extensive parameter variation or high specimen replication in a parallel series, reflecting higher complexity in specific dimensions. The synthesis applies a multidimensional interpretation at the test level to capture differences across experimental programs that a single COI score may overlook. Each test component is evaluated independently. A practical example illustrates the benefit of this approach. A traditional classification based only on study counts might conclude that unanchored EB-FRP systems dominate the available evidence because COI-A contains substantially more test results than COI-B or COI-C. However, the multidimensional interpretation shows that several questions of direct design relevance, including anchor spacing effects, load sharing among anchors, and system-level force redistribution, can only be addressed using COI-C studies. Consequently, the framework distinguishes between the quantity of available evidence and the scope of conclusions that can be drawn from that evidence. This distinction directly influenced the synthesis by preventing high-volume COI-A datasets from being interpreted as evidence for phenomena that can only be evaluated through higher-complexity anchored-system investigations. The four dimensions from the COI framework are repurposed as analytical categories to identify trends, methodological emphasis, and research gaps across all test programs:

(1) Experimental objectives and parameter selection.
(2) Instrumentation and loading protocol.
(3) Data acquisition and post-processing.
(4) Failure mode analysis and theoretical development.

**Figure 6:**
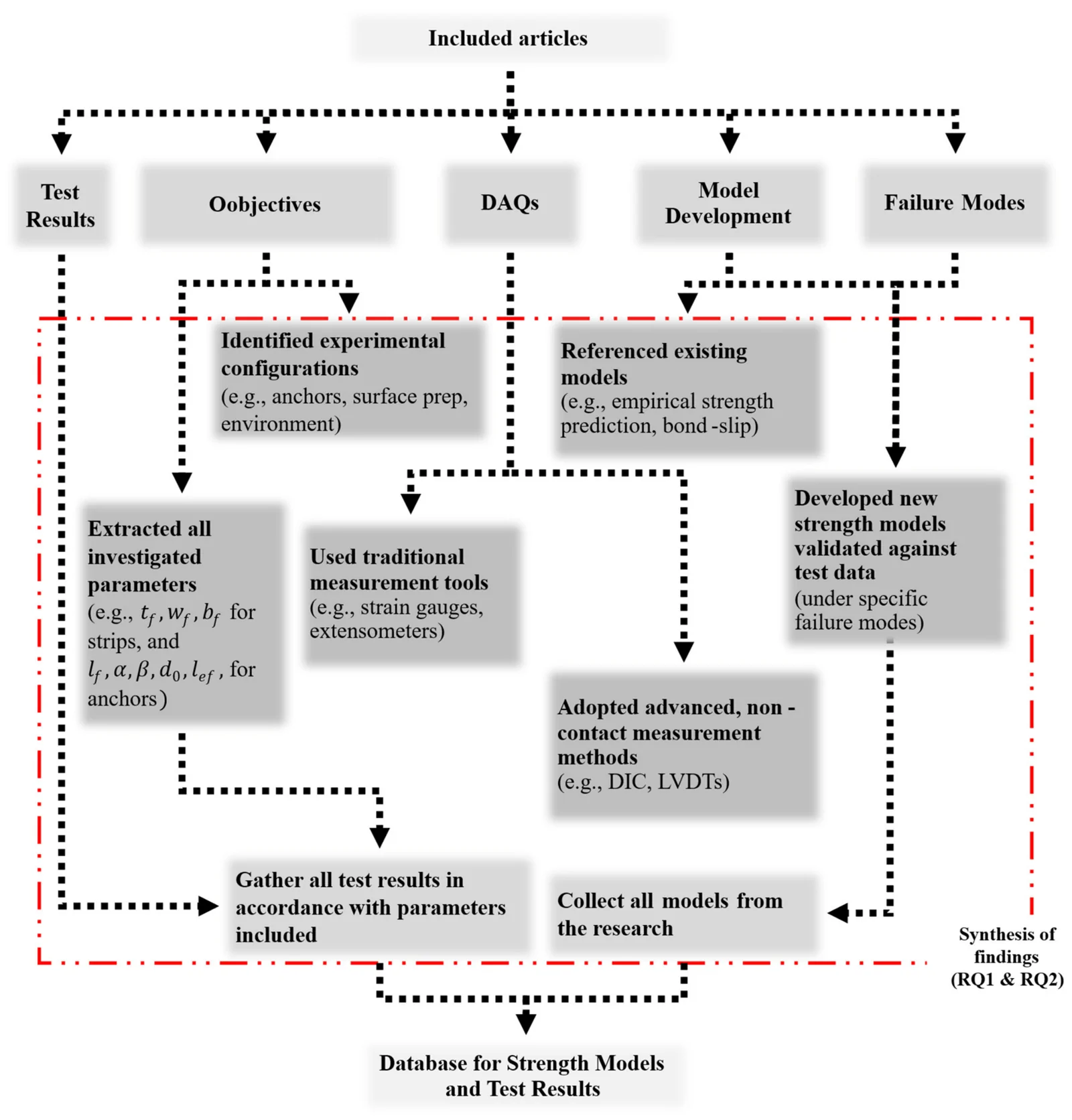
Schematic framework for interpreting and synthesizing test-level findings to address RQs 1 and 2.

A test-level, aspect-based framework supports systematic comparison and helps structure findings for RQs 1 and 2. It also allows for cross-study comparison and supports the organization of bond models, anchorage schemes, and parameter ranges that may inform future benchmarking or database development. Figure 6 shows the synthesis framework, outlining how test-level findings were structured according to these four analytical categories.

Several prior review studies informed the development of this framework. Ref. [50] evaluated over 1000 tests from 143 studies, focusing on axial tensile performance in EB-FRP systems with and without anchors, and benchmarked results against ACI 440.2R, CNR-DT 200, and fib Bulletin 90. Ref. [67] analyzed the influence of FRP anchor configurations, confirming their effect in delaying premature debonding and enhancing force transmission. Ref. [68] compared EB-FRP systems with and without grooving techniques, highlighting how interface geometry, composite stiffness, width ratio, and area ratio influence failure mechanisms. Insights from these reviews guided the development of the analytical dimensions used to interpret the current dataset. The 174 experimental test programs were synthesized using four analytical dimensions, enabling direct comparison of bond behavior in unanchored and anchored EB-FRP systems under tension or lap-shear loading. The interpretation focuses on performance patterns, anchor effectiveness, and experimental limitations, directly addressing RQs 1 and 2, while also contributing to RQ3 by identifying unresolved challenges and outlining future research directions.

## 3. Bibliometric Analysis and Research Landscape

### 3.1. Keywords and Regional Research Network Mapping

Keyword co-occurrence analysis was conducted using VOSviewer (version 1.6.20) to identify dominant research topics and thematic structures across the 174 included studies. This tool applies statistical and network-based methods to generate scientific landscape visualizations that reveal conceptual relationships between key terms. The analysis focused on experimental studies related to EB-FRP systems under direct tension and lap-shear testing. A custom thesaurus spreadsheet was developed to clean and consolidate the keyword dataset. Redundant terms were merged to improve clarity and avoid duplicate entries. For example, variations such as “single lap-shear test” and “single lap-shear tests” were unified, as were “delaminating” and “delamination”. Abbreviations and synonymous terms were consolidated where appropriate. For example, “Glass fiber/fiber,” “GFRP,” and “Basalt fiber reinforced polymer” were grouped under the general term “FRP.” However, CFRP was intentionally retained as a distinct keyword in the network. Due to its dominant role in EB-FRP research, particularly in externally bonded systems, CFRP frequently appears in published studies and was not merged into a broader category. Maintaining CFRP as a separate node allowed its influence and connections in the literature to be visualized more accurately. Terms related to anchorage, such as “spike anchors” and “anchor(s)”, were merged as “FRP anchors” or “fiber anchors”. Generic or irrelevant terms, such as “repair,” “issue,” and “sun,” were excluded.

**Figure 7:**
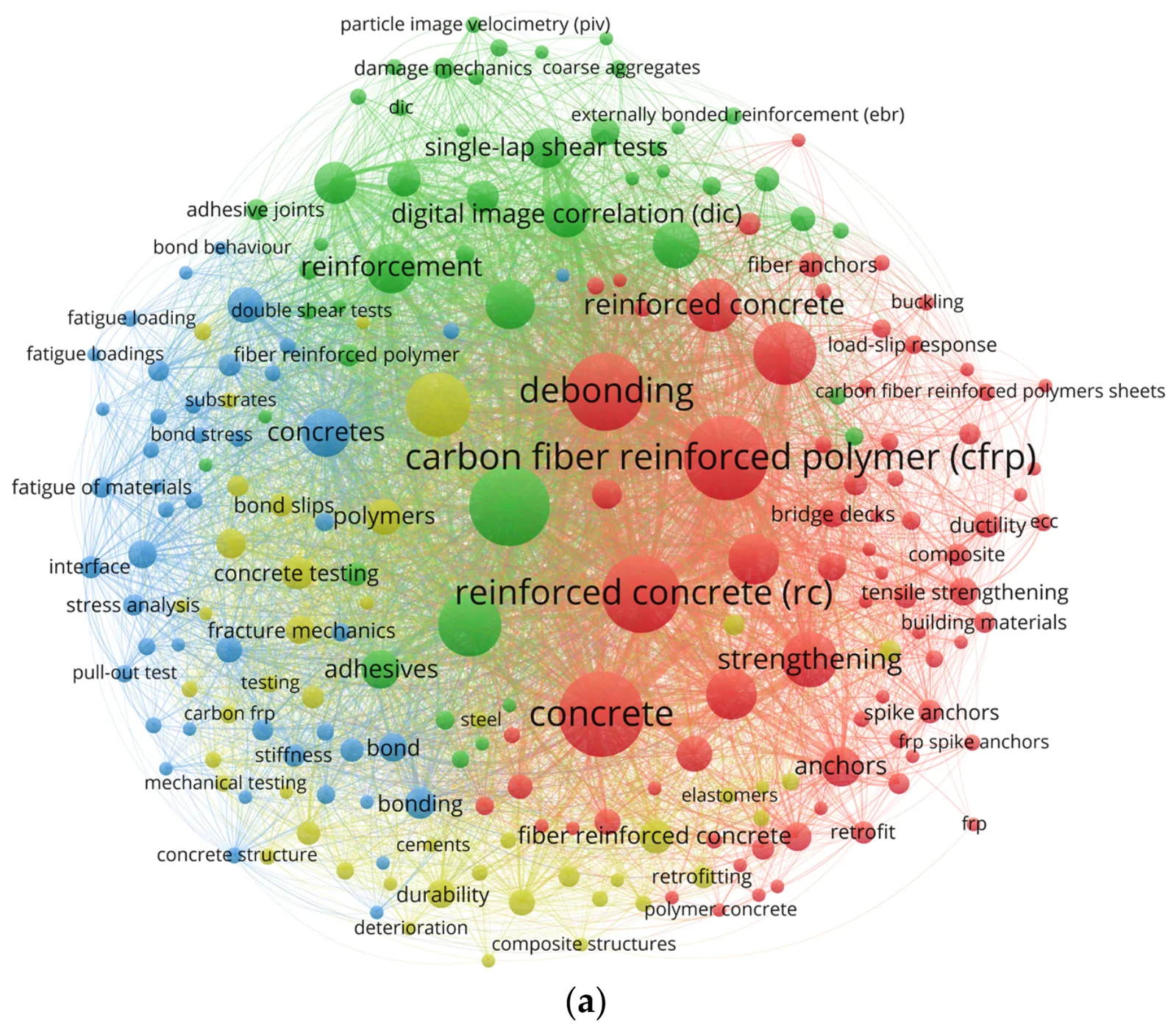

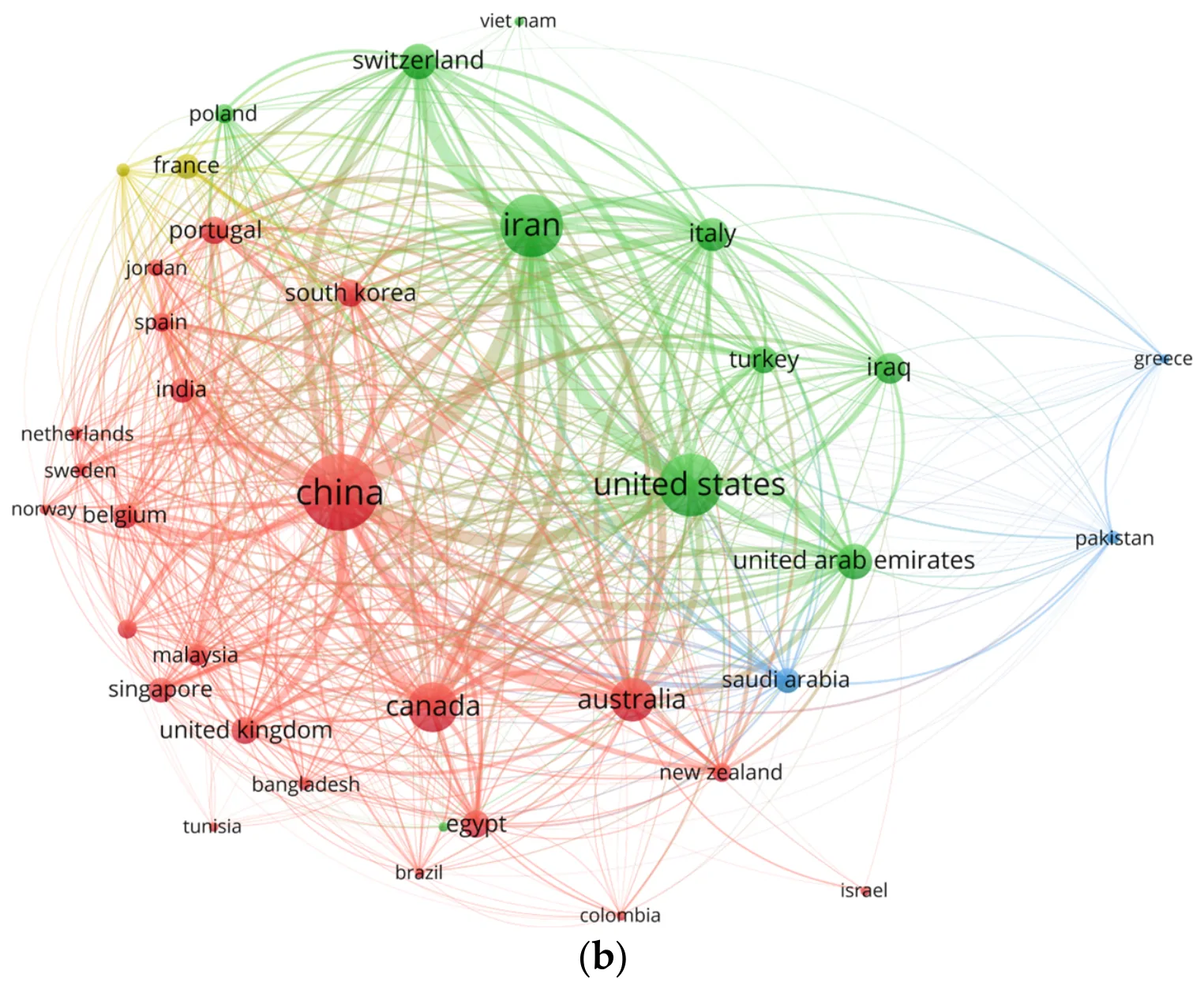
Keywords network visualization: (**a**) keyword co-occurrence network and (**b**) regional research collaboration network for the reviewed studies.

Figure 7a shows the keyword network map after term consolidation. A minimum threshold of three occurrences per keyword was applied, resulting in 214 keywords selected from the original 1453. Prominent terms include “FRP/CFRP”, “strengthening”, “externally bonded”, “(single-) lap shear tests”, “bond behavior”, “anchorage systems”, “concrete/reinforced concrete (RC)”, “debonding”, and “digital image correlation (DIC)”, all of which align with the core themes of this review. In parallel, geographic publication trends were examined through country-level co-authorship mapping. Figure 7b shows the international distribution of EB-FRP research contributions. Node size reflects the number of publications, while link thickness represents co-authorship intensity. China, Iran, and the United States emerged as the most active contributors. Strong collaboration clusters were observed between institutions in Asia, North America, and Europe, with notable co-authorship linkages among China, Iran, Italy, and the United States. The map also reveals regions with limited representation, indicating geographic variation in research activity and uneven levels of engagement with EB-FRP experimental studies. The country-level map was used to describe collaboration and publication concentration, not to infer national differences in test philosophy. Although regional clusters may reflect different equipment, code environments, or preferred test methods, the extracted variables did not support a reliable country-by-country methodological ranking. The review therefore limits country interpretation to research activity and collaboration patterns and treats methodological differences through test type, COI level, and coded experimental variables.

A preliminary comparison of the leading country clusters suggests differences in predominant experimental emphases, although regional trends should not be interpreted as fixed national scientific schools. China and the broader East Asian research cluster accounted for approximately 26.0% of the included studies, followed by the European cluster led by Italy (18.1%), the United States and North American institutions (17.7%), Iran (13.0%), and Australia/New Zealand (5.8%). These five research clusters contributed more than 80% of the included studies, indicating that EB-FRP bond and anchorage research has been concentrated within a limited number of geographical regions. Studies from the China and broader East Asian cluster frequently investigated FRP–concrete bond behavior, bond–slip modeling, and plate or sheet interface testing. Iranian studies commonly examined CFRP sheet-to-concrete bond, EBROG and EBR comparisons, aggregate and concrete effects, and environmental or surface-condition variables. North American studies emphasized design-oriented strengthening, anchored FRP systems, mechanically or fiber-anchored configurations, and structural-member behavior. European contributions, particularly Italian studies, focused on externally bonded reinforcement terminology, interface and fracture mechanics, durability, and guideline-oriented interpretation. Country attribution was based on the first-author or corresponding author affiliation of the included studies. The extracted database does not support a rigorous country-by-country methodological ranking because collaboration networks, co-authorship patterns, and experimental variables remain highly heterogeneous. Regional interpretation is therefore presented only as a descriptive trend. Table 9 summarizes the approximate representation, research emphases, and predominant experimental tendencies associated with the major regional clusters.

### 3.2. Publication Patterns

The publication sources of the 174 included articles are summarized in Table 10, categorized by journal title, publisher, SJR quartile ranking, H-index, impact factor, and publication year. All journals were indexed in the SCImago Journal Rank (SJR) system, with a total of 39 journals represented. Among these, 21 were Q1 journals, five were Q2, and three were Q3; no articles appeared in Q4 journals. The majority of papers, 156 in total (89.7%), were published in Q1 journals, reflecting the field’s emphasis on high-impact, technically rigorous outlets. Q2 journals accounted for 14 papers (8.0%) and Q3 journals for four papers (2.3%). Publication activity increased substantially over time. Only six papers (3.5%) were published before 2003, while more than 83% appeared after 2013. The period from 2018 to early 2026 saw the highest concentration, with 99 articles (56.9%) published, indicating growing research interest in the interfacial behavior of EB-FRP systems under direct tension and lap-shear testing. Journals most frequently publishing on this topic include *Composites for Construction* (ASCE, 34 papers), *Composite Structures* (Elsevier, 27), *Construction and Building Materials* (Elsevier, 25), and *Composites Part B: Engineering* (Elsevier, 21). Together, these four journals contributed over 60% of the reviewed literature. Other notable contributors include *Engineering Structures*, *Engineering Fracture Mechanics*, *Journal of Reinforced Plastics and Composites*, and *ACI Structural Journal*. Elsevier was the leading publisher, accounting for 56.3% of the included papers. The American Society of Civil Engineers (ASCE) followed with 21.8%. Other publishers included SAGE (5%), ACI (4%), Springer (3%), MDPI (2%), Hindawi (2%), and ICE Publishing (2%). Single-paper contributions came from ASTM, Taylor & Francis, Techno Press, Wiley-Blackwell, and the National Research Council of Canada.

**Figure 8:**
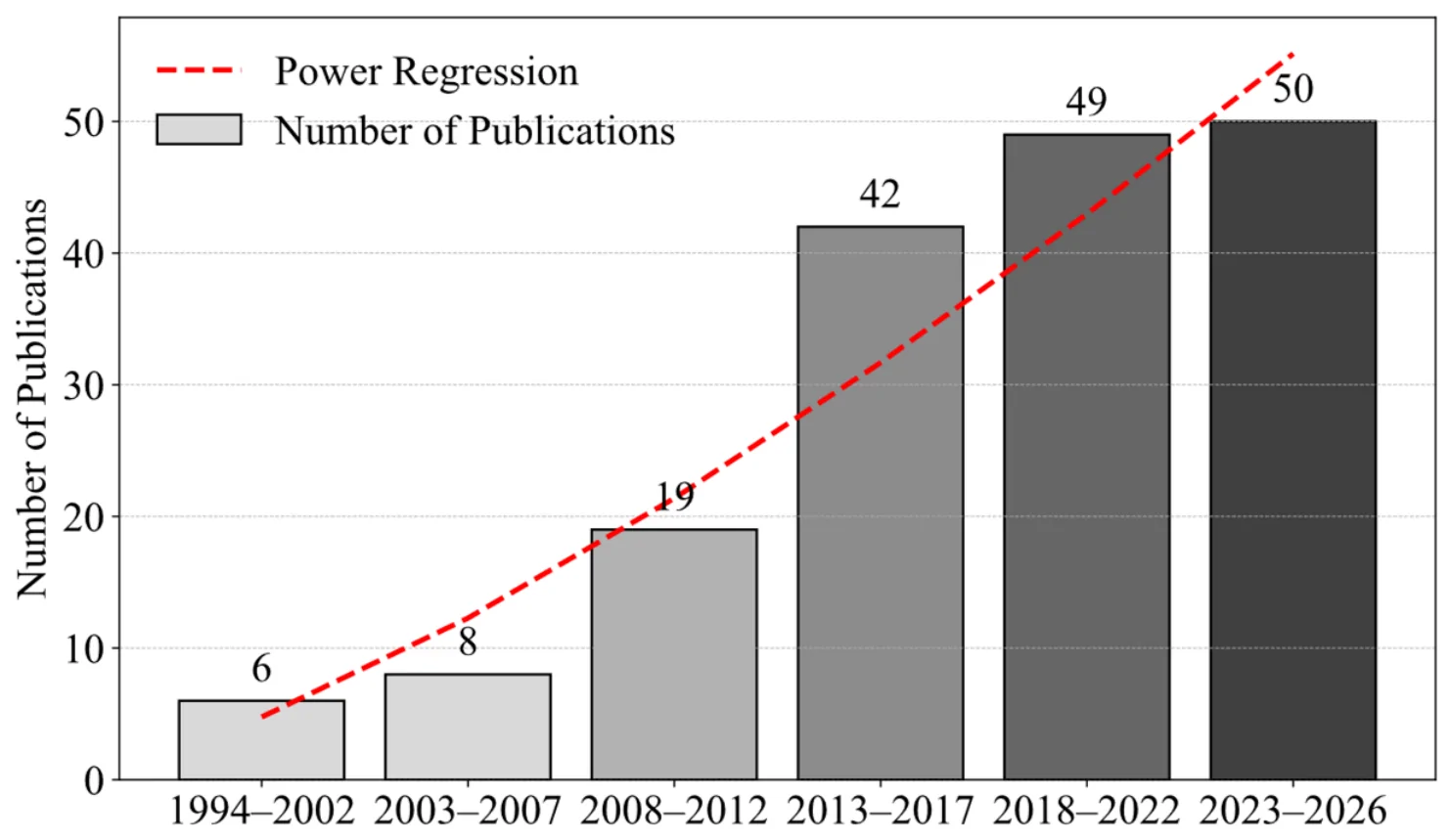
Temporal distribution of publications on EB-FRP bond and anchorage research from 1994 to 2026.

Figure 8 shows the temporal distribution of publications, grouped in five-year intervals from 1994 to 2026. Power and exponential regression models were applied to assess publication growth. The power model produced an *R^2^* value of 0.9577, suggesting a strong fit. Extrapolating from this model, publication volume is expected to continue rising, though a plateau may begin to emerge beyond 2026. As of mid-February 2026, 50 papers had already been published during the current five-year window. Overall, the data confirm both the concentration of EB-FRP bond research in high-ranking journals and its accelerating trajectory in recent years. These trends reflect the field’s increasing technical maturity and broadening relevance within structural engineering research.

### 3.3. COI-Based Categorization

**Figure 9:**
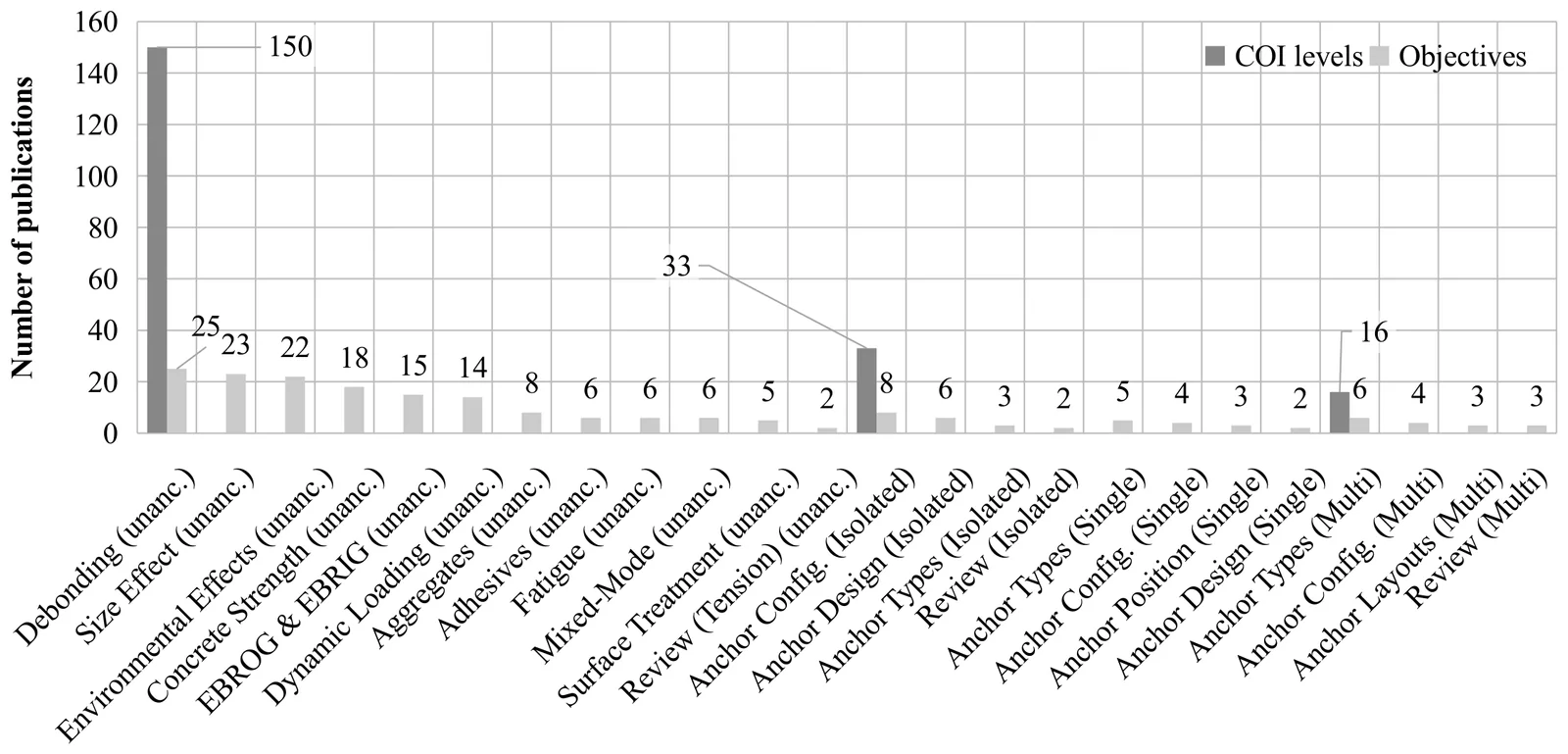
Classification distribution of research objectives.

Figure 9 shows the classification of 174 publications by Complexity of Investigation (COI). Dark columns represent COI levels, and light columns reflect sub-objectives within each level. Most publications (150 out of 174) fell under COI-A, representing studies on unanchored EB-FRP systems subjected to axial tension. Most investigations focused on interfacial bond behavior, including debonding mechanisms, FRP strip size effects, and environmental degradation. Frequently addressed sub-objectives included debonding (25 publications), geometric parameters (23), environmental exposure (22), and concrete strength (18). Fewer studies examined EBROG and EBR systems (15), dynamic effects (14), aggregate properties (eight), adhesives (six), fatigue (six), mixed-mode fractures (six), surface treatments (five), and general reviews (two). Although substantial in quantity, most of these studies investigated isolated parameters under simplified testing conditions. Limited integration across variables restricted the extrapolation of findings to real applications.

The studies involving FRP anchors under axial tension, grouped under COI-B, remained comparatively underrepresented, with 33 publications (19.3%). This category included both isolated anchor tests and single-anchor EB-FRP systems. COI-B1 contained 19 studies that examined isolated anchors without integration into EB-FRP assemblies. Most employed pullout tests or single-lap shear configurations. The primary objectives addressed anchor configuration (eight publications), design methodology (six), and anchorage typologies (three). Two studies provided review-based summaries. These investigations typically relied on idealized test conditions. Although effective in characterizing basic anchor performance, the absence of system-level interaction limited the applicability of results to practical strengthening scenarios. COI-B2 included 14 studies that incorporated single anchors within EB-FRP systems. These investigations examined anchorage typologies (six publications), configuration (three), anchor positioning (three), and design parameters (two). Although more representative of field conditions than COI-B1, inconsistencies in geometry, installation, and substrate conditions prevented meaningful synthesis and comparison across studies. This variation impeded the development of broadly applicable design recommendations.

COI-C represented the highest level of complexity, encompassing 16 studies (9.4%) involving EB-FRP systems with multiple FRP anchors. Research objectives within this group addressed anchor layout strategies (six publications), configuration effects (four), number and positioning of anchors (three), anchorage typologies (three), and system-level reviews (three). These studies introduced added complexity by accounting for scale effects, anchor-to-anchor interactions, and more realistic boundary conditions. Despite these advancements, limited sample sizes and diverse methodologies hindered a comprehensive evaluation of overall system performance. While the total number of reviewed publications remained fixed at 174, many studies investigated more than one objective. For instance, articles categorized under the concrete strength objective in COI-A often also examined environmental exposure or FRP geometry. This overlap led to a total number of sub-objective classifications exceeding the number of unique studies, highlighting the multidimensional nature of the investigations and the need for integrated frameworks capable of capturing parameter interactions across COI levels.

### 3.4. Types of DAQs, FRPs, and Experimental Programs

**Figure 10:**
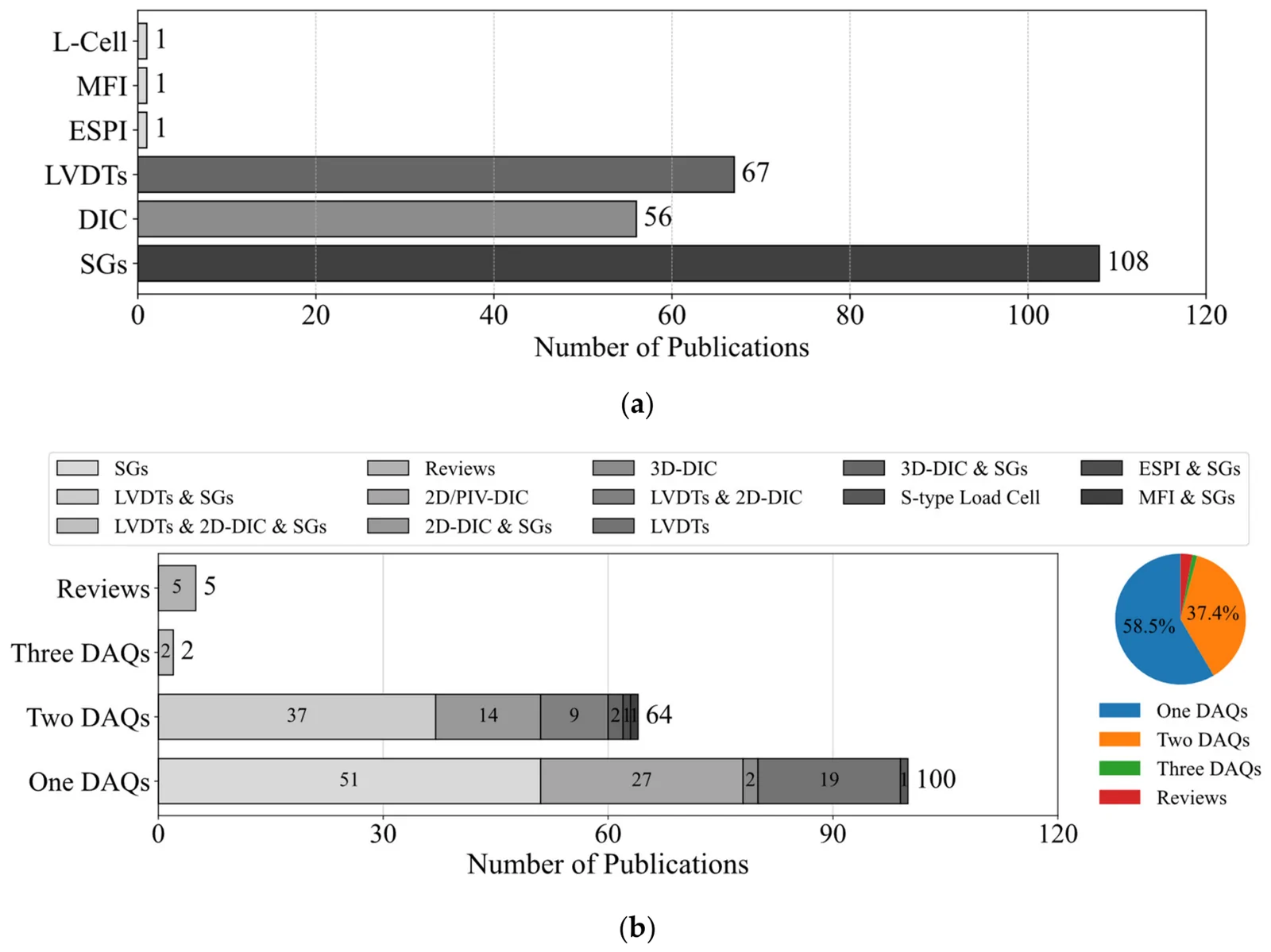
Distribution of DAQ methods used in the reviewed studies: (**a**) types of DAQs and (**b**) distribution of DAQ combinations.

Accurate measurement of deformation or strain is critical in FRP-to-concrete bond testing. Various data acquisition (DAQ) strategies were employed in the reviewed literature, as shown in Figure 10a. The most common method was the use of strain gauges (SGs), adopted in approximately 62.1% of studies (108 out of 174). A significant portion of these studies (52.8%, or 57 out of 108) used SGs only as auxiliary tools to validate measurements at discrete points. Digital image correlation (DIC) was the second most widely used technique, applied in 56 studies (32.7%), either in 2D or 3D configurations, depending on the number of cameras used. DIC enabled full-field strain mapping and often served as the primary DAQ system in recent works [11,21,43,69]. Linear Variable Differential Transformers (LVDTs) were used in 67 studies (39.2%), though only a small subset (19 studies) relied solely on LVDTs for all deformation measurements. Two interferometry-based approaches were identified, with Electronic Speckle Pattern Interferometry (ESPI) and Moire Fringe Interferometry (MFI). Both were used in conjunction with SGs for hybrid strain field capture. Only one study employed S-type load cells as the sole deformation recording tool.

The number and combinations of DAQ devices are summarized in Figure 10b. Just over half of the reviewed studies (58.5%, or 100 out of 151) used a single type of DAQ device. A total of 64 studies (37.4%) employed two DAQ systems simultaneously, while only two studies utilized three. Most combinations (37 publications) involved traditional devices, such as SGs and LVDTs, used to validate data collected from full-field optical systems. Notably, 2D-DIC, 3D-DIC, and Particle Image Velocimetry (PIV-DIC) have shown increasing adoption in configurations involving more than two DAQ systems (27 publications) due to their capacity to resolve continuous strain fields across the bond zone.

**Figure 11:**
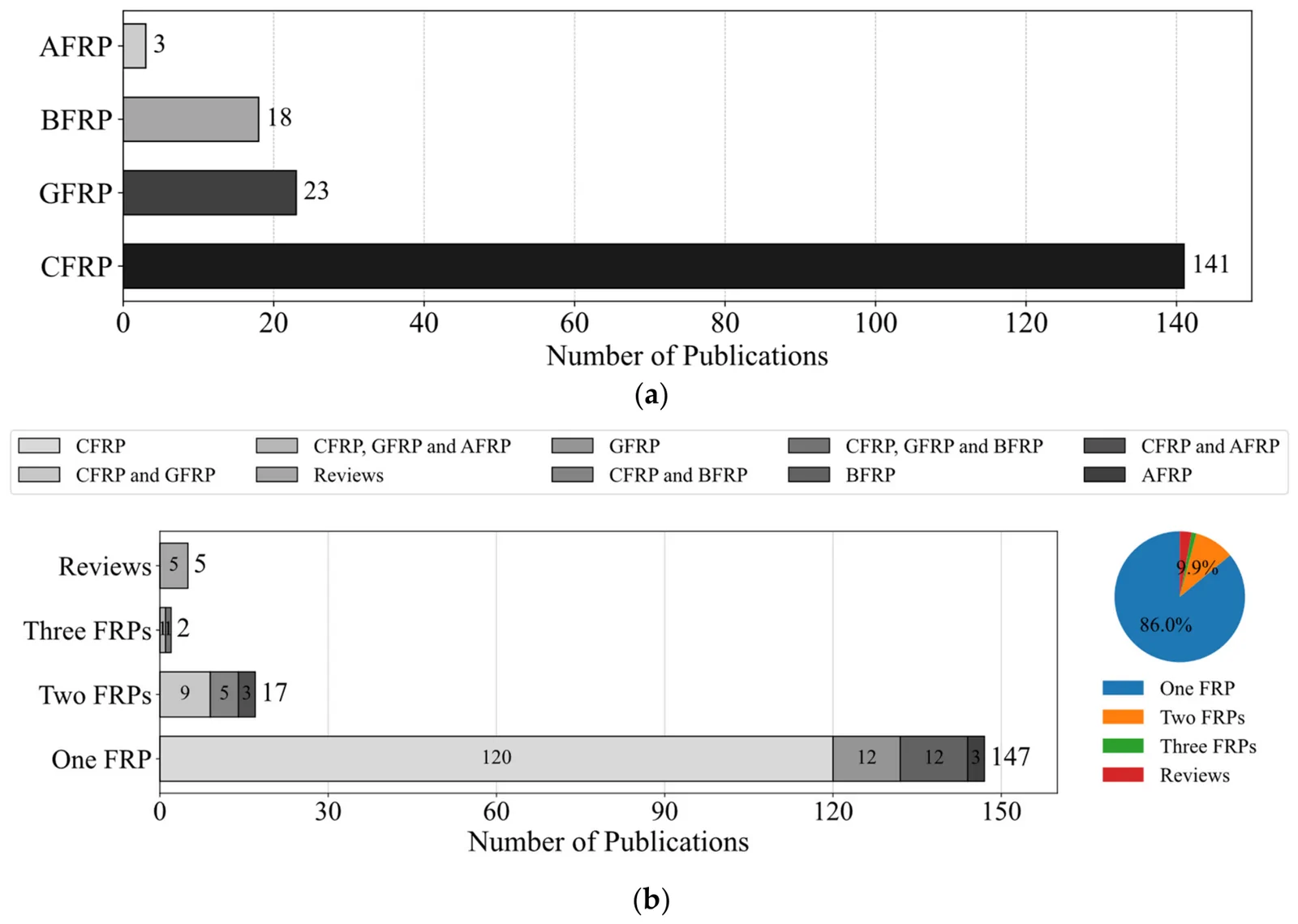
Distribution of FRP material types used in the reviewed studies: (**a**) types of FRPs and (**b**) distribution of FRP combinations.

Varied FRP materials were used in the reviewed experimental programs, as shown in Figure 11. Carbon fiber-reinforced polymer (CFRP) dominated the selection, appearing in 143 studies (83.6%). Other types included Glass FRP (GFRP) in 23 studies (13.5%), Basalt FRP (BFRP) in 18 studies (10.5%), and Aramid FRP (AFRP) in seven studies (4.1%). Some publications employed more than one FRP type for comparative analysis. As shown in Figure 11b, 147 studies (86%) used a single FRP type, while 17 studies (9.9%) used two types, and two studies (1.2%) used three.

The unequal distribution of FRP types affects the strength of material-specific conclusions. CFRP provides the highest-confidence evidence base because it appears in 143 studies and dominates both unanchored and anchored datasets. GFRP, BFRP, and AFRP are represented by 23, 18, and seven studies, respectively, so trends for these materials should be interpreted as indicative rather than definitive. Available studies suggest that differences in stiffness and material behavior may influence load-transfer demand, interfacial stress distribution, and debonding response [70], but the limited number of direct comparative studies prevents robust quantitative comparison among all FRP families. Conclusions that use the general term FRP, therefore, mainly reflect CFRP-dominated evidence, unless a specific comparison among GFRP, BFRP, and AFRP is stated. A summary of the evidence distribution, interpretive confidence, and principal implications for each FRP material system is provided in Table 11.

**Figure 12:**
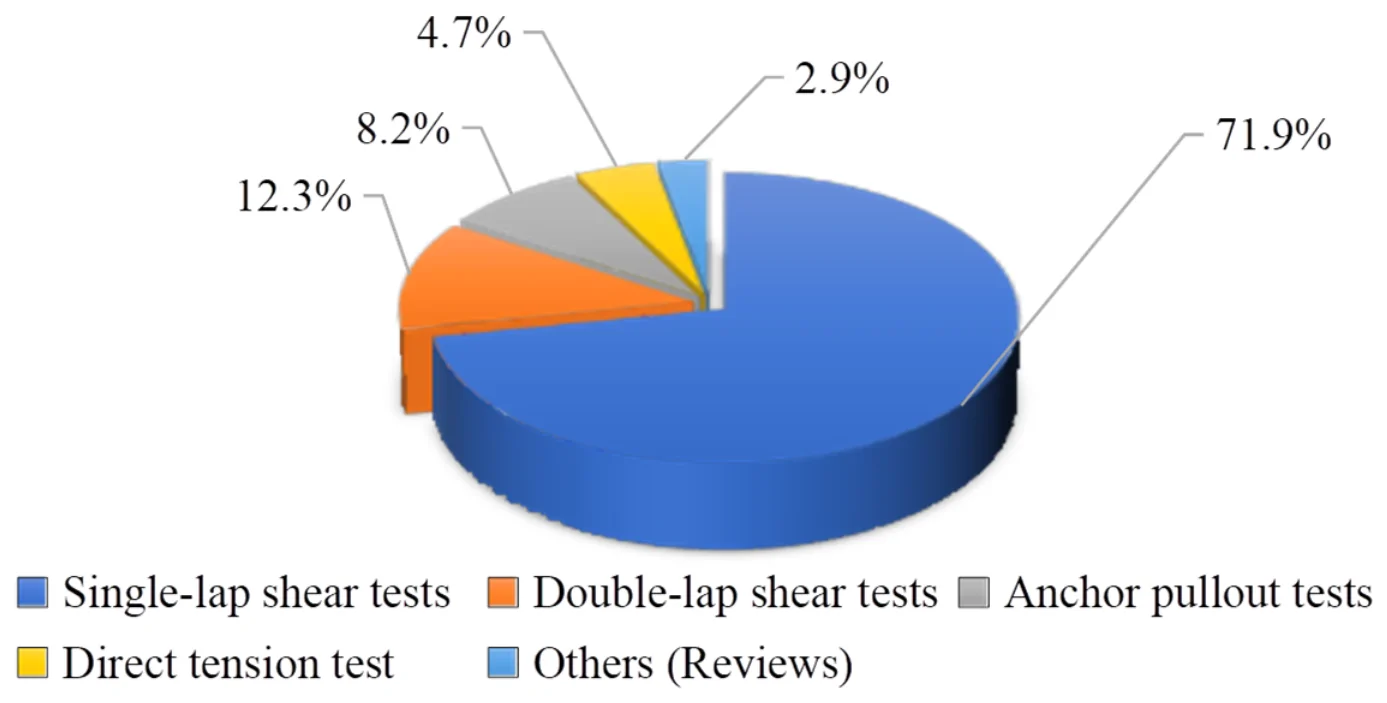
Distribution of experimental programs.

The experimental programs adopted across the reviewed studies are shown in Figure 12. Single-lap shear configurations dominated, as reported in 123 publications (71.9%). Double-lap shear tests appeared in 21 studies (12.3%), primarily investigating interfacial bond behavior between FRP sheets and concrete substrates, between fan-shaped FRP anchors and bonded strips, or isolated bent fiber anchors under shear, corresponding to COI-A, COI-B2, and COI-C levels. Direct tension tests, reported in eight studies (4.7%), applied tensile force through embedded reinforcement in concrete (COI-A) to examine the bond between FRP configurations and reinforced concrete. These tests aimed to replicate realistic loading patterns where concrete acted as the primary structural element. Pullout tests, reported in 12 publications (8.2%), applied uniform axial tension directly on the anchor dowel to isolate anchorage performance (COI-B1). These tests typically assessed anchorage effectiveness, dowel geometry, and fan configurations under controlled slip conditions, or they investigated specific failure patterns.

## 4. EB-FRP Without Anchors (COI-A)

### 4.1. Overview

A systematic analysis of unanchored EB-FRP strengthening systems for concrete is presented in this section under COI-A classification. The discussion addresses RQ1 and integrates bibliometric context to interpret trends in research focus, experimental methods, and theoretical developments. Studies are organized by experimental parameters, mechanical behavior, failure modes, and model contributions, as summarized in Table 12.

### 4.2. Test Methods

**Figure 13:**
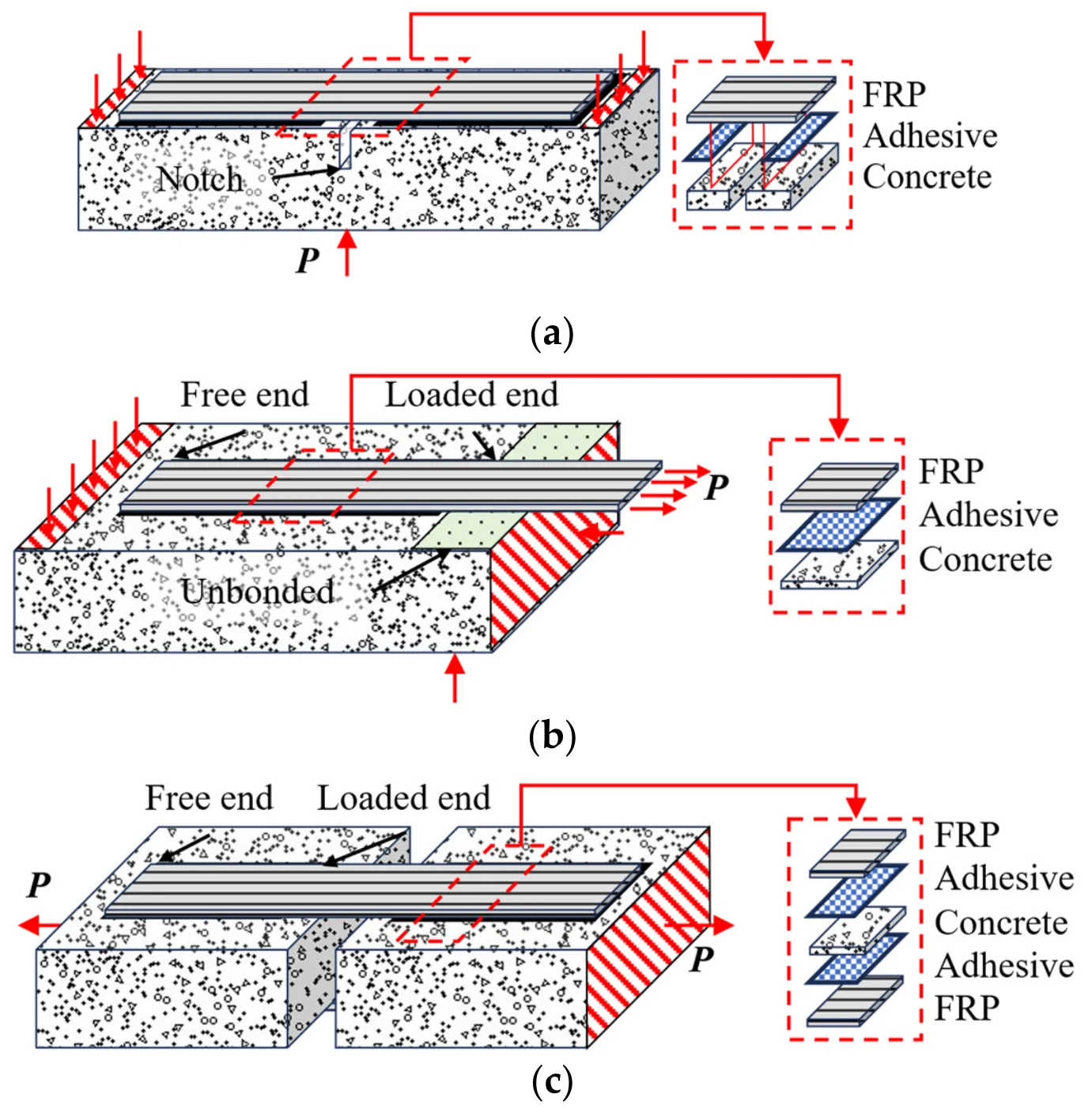
Experimental configurations used for evaluating FRP–concrete bond behavior: (**a**) beam tests (bottom side view), (**b**) single-lap shear tests, and (**c**) double-lap shear tests.

Indirect and direct shear tests are widely used to investigate interfacial behavior and failure mechanisms between FRP and concrete in COI-A studies. Both methods evaluate bond performance and identify conditions that lead to debonding or fracture. The notched beam test shown in Figure 13a is an indirect method where the FRP strip response can be affected by the concrete prism deformation because of the presence of the bending moment [20]. Single-lap and double-lap shear tests are direct shear tests widely adopted in previous research. The single-lap shear test shown in Figure 13b is commonly used for evaluating interfacial behavior due to its relative simplicity [137]. In this configuration, the loaded end refers to the beginning of the bonded area where the load is applied, while the opposite end is known as the free end. The concrete prism is subjected to compression forces at both the loaded and free ends by a restraining frame, such that the concrete becomes a rigid body that cannot crack. An unbonded area is often introduced near the edge of the specimen to prevent undesired concrete cone failure. The double-lap shear tests (Figure 13c) were introduced as an alternative method to eliminate the eccentricity caused by the tensile load applied to the FRP and the reaction force on the concrete, which can lead to out-of-plane deformations that may affect the results. Despite the symmetry of the double-lap test configuration, debonding typically initiates at the weakest interface due to material variability, causing an asymmetry in the interfacial bond. This asymmetry ultimately leads to rotation of the specimen during testing, affecting the accuracy of the results [138]. A comparative summary of the principal test methods, their advantages, limitations, and relevance to practical applications is presented in Table 13.

The test methods are therefore not interchangeable. Single-lap shear tests provide simple force measurement and are well suited to comparative bond-capacity screening, but eccentricity and fixture restraint can affect the local stress field. Double-lap shear tests reduce global eccentricity and are useful for symmetric load transfer, although debonding commonly initiates at the weaker side and may still create rotation. Direct tension tests better represent axial load transfer in members, but are less common and harder to standardize. Pullout tests isolate anchor dowel behavior and are useful for straight-anchor capacity, but they do not by themselves represent anchor–strip interaction in an EB-FRP system. Flexural or notched-beam arrangements can reproduce structural bending effects but introduce additional concrete-prism deformation and are less direct for pure interface comparison. No single test method can be regarded as universally superior because each isolates a different aspect of EB-FRP behavior. Direct tension tests generally provide the closest representation of the axial force-transfer mechanisms encountered in tension-strengthened members because they directly capture force transfer between the FRP and concrete substrate under tensile loading. In contrast, lap-shear and pullout tests remain indispensable for mechanistic investigation because they isolate specific bond and anchorage variables that cannot be easily distinguished in larger structural tests. Flexural and notched-beam configurations provide additional insight into the influence of bending and cracking on interfacial behavior, although the resulting stress state is more complex and less suitable for isolating pure bond mechanisms than direct-tension or shear-based tests.

**Figure 14:**
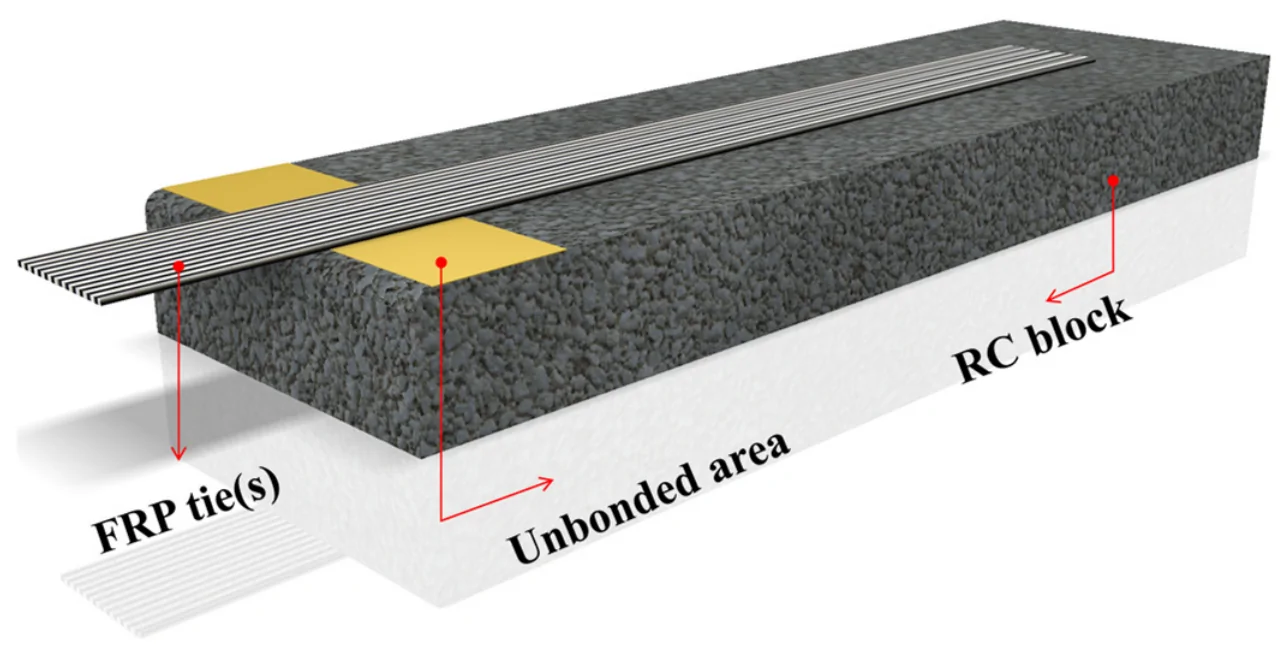
Typical specimen for EB-FRP single lap shearing tests.

Results from the tests provide the basis for interfacial behavior models referenced in design standards, including CNR DT 200 [48], CNGB [139], ACI 440.2R-17 [47] and fib bulletin 90 [49]. The ASTM D8337/D8337M-21 [140] designates the single-lap shear test as the preferred method for determining bond capacity due to straightforward setup and direct measurement of applied forces (Figure 14).

### 4.3. COI-A Dataset

#### 4.3.1. Collected Test Results

**Figure 15:**
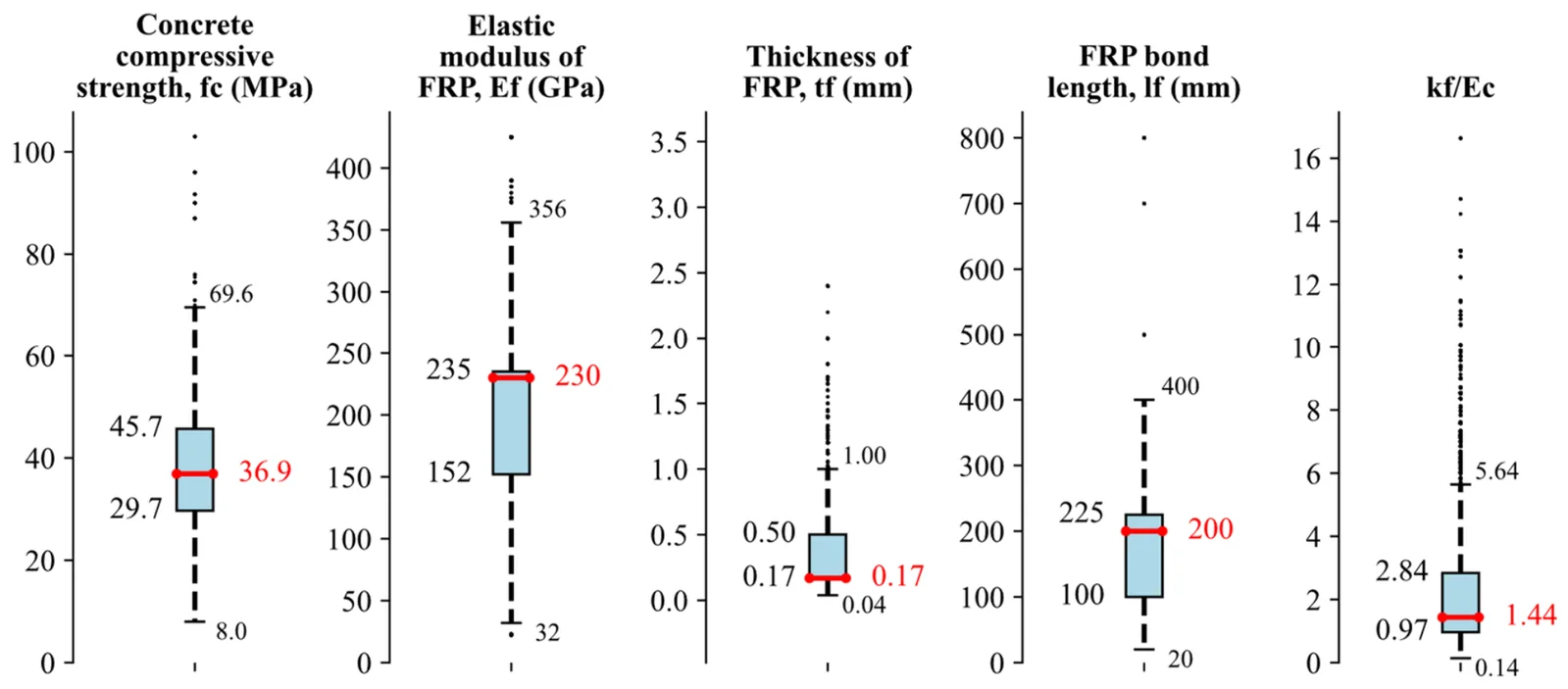
Distribution of key experimental parameters extracted from the literature database. Red lines and values indicate median values of the corresponding parameters. [21,50].

A total of 3162 test results under COI-A were compiled in Ref. [28]. The dataset was built from experimental data extracted from the following included studies [9,22,23,24,27,30,51,52,53,55,56,57,58,59,60,71,72,73,74,75,76,77,78,79,80,81,82,83,84,85,86,87,88,90,91,92,93,94,95,96,97,98,99,100,101,102,103,104,105,106,107,108,109,110,111,112,113,114,115,116,117,118,119,120,121,122,123,124,125,126,127,128,129,130,131,132,133,134,135,141,142,143,144,145,146,147,148,149,150,151,152,153]. The collected parameters include specimen geometry, FRP strip dimensions, material properties (e.g., elastic modulus and tensile strength), and recorded ultimate capacity. Box-and-whisker plots were used to illustrate the spread of the data and highlight the distribution of key experimental parameters, as shown in Figure 15. These visualizations reveal trends and limitations in the existing research. Most studies were based on small-scale test configurations, typically employing short FRP ties (median length: 200 mm), thin laminates (median thickness: 0.17 mm), and low stiffness ratios, with the average value of ${k_{f}/E}_{c}$ approximately 1.4. Here, $k_{f}$ denotes the FRP stiffness ( $NE_{f}t_{f}$ ), and $E_{c}$ is the modulus of elasticity of concrete. These test conditions do not adequately represent real-scale structural behavior. Prior studies [11,69] have shown that large-sized (thicker and longer) single-lap shear tests exhibit markedly different performance characteristics, including increased load capacity and extended post-debonding deformation. The lack of validation and limited availability of large-scale EB-FRP test data continue to constrain the development of models for practical field applications [50].

Parameter-level synthesis indicates that unanchored EB-FRP performance is governed by interacting material, geometric, interface, and exposure variables rather than by any single property. Higher FRP stiffness, greater strip thickness, and wider bonded areas can increase capacity but also intensify interfacial stress gradients, making debonding strain, effective bond length, and failure mode sensitive to adhesive stiffness and concrete surface quality. Increasing bond length improves force transfer only until the effective bond length is reached; beyond that point, additional length may add limited capacity but can change post-peak load–slip behavior. Concrete strength and aggregate structure influence substrate fracture and concrete-cohesive debonding, while surface treatment and adhesive properties control whether failure localizes in the adhesive, at an interface, or in the near-surface concrete. Environmental exposure, including elevated temperature, wet or hygrothermal aging, freeze–thaw cycling, and long-term outdoor exposure, mainly affects adhesive-dependent load transfer and residual bond capacity; because these studies use different exposure protocols, their results are best interpreted as durability risk trends rather than a single pooled degradation factor. The principal exposure mechanisms, recommended reporting metrics, and current comparability limitations are summarized in Table 14.

Field-transfer limits were also made explicit because most included tests remain on a laboratory scale. Laboratory coupon tests isolate mechanisms, but field strengthening must contend with substrate variability, surface preparation quality, adhesive curing, access constraints, construction tolerances, in-service moisture and temperature, and multi-strip or multi-anchor load paths. These factors do not invalidate small-scale tests, but they limit direct extrapolation unless the model or design rule is checked against larger members or field-representative system tests.

#### 4.3.2. Fracture Mode II Theory and Existing Models

**Figure 16:**
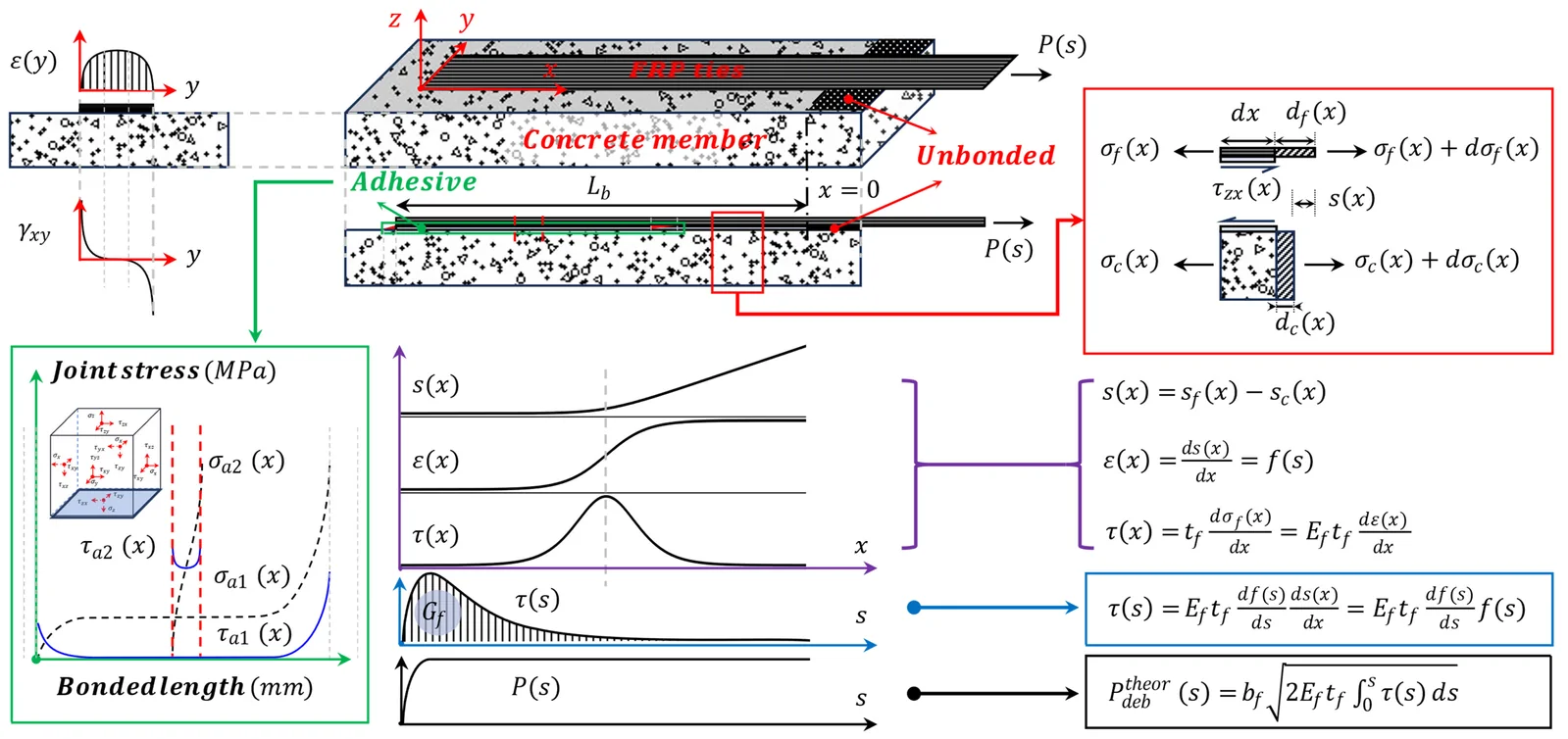
Analytical framework for Mode II fracture of the FRP–concrete interface. The green region illustrates the adhesive layer and stress distribution within the bonded joint, the red region shows the local equilibrium and stress-transfer mechanism, the purple region presents the slip, strain, stress, and bond–slip relationships, the blue arrows indicate the governing analytical relationships, and the black box represents the theoretical debonding force formulation.

Fracture Mode II is a commonly used theory to analyze lap shear tests and evaluate interfacial fracture based on fracture mechanics principles. Figure 16 shows the mechanisms of Mode II fracture within an FRP–concrete interface bonded with adhesive. The load applied, denoted as P(s) , is transmitted from the FRP to the concrete through to the bonded region, generating both normal and shear stresses along the interface. The normal stresses are represented as $\sigma_{f}(x)$ in the FRP and $\sigma_{c}(x)$ in the concrete, while $\tau_{zx}(x)$ represents the shear stress at the interface. As the slip s(x) between the FRP and the concrete increases, these stresses change, making them critical in predicting debonding behavior [154].

The normal strain ε(y) distribution across the width of the interface has parabolic peaks at the center and diminishes toward the edges [155,156], as shown at the top left in Figure 16. The central region carries the majority of the load, while the edges experience minimal deformation. The shear strain $\gamma_{xy}$ is maximal at the edges and decreases toward the center, highlighting that shear deformation is most prominent along the edges of the bond. This emphasizes that load transfer occurs predominantly in the central region of the bond, with shear stresses being concentrated near the edges. The stress distribution within the adhesive joint along the length is shown in the lower left corner of Figure 16 in a green box, which shows two cases. Case 1, the blue solid line ( $\sigma_{a1}$ and $\tau_{a1}$ ), represents a scenario with a short bond length, while case 2, the dashed black line ( $\sigma_{a2}$ and $\tau_{a2}$ ), represents a scenario with a long bond length. As bond length increases, normal stresses $\sigma_{a1}(x)$ become more significant than shear stresses $\tau_{a1}(x)$ in the middle portion, where shear stresses reduce to nearly zero, while normal stresses remain constant. Shear stress peaks appear at the bond ends $L_{b}$ , where debonding typically begins. The shear stress $\tau_{a2}(x)$ curve becomes asymmetrical when the bonded materials have differing rigidity, leading to high stress peaks at the ends of more rigid concrete members, while the less rigid components undergo greater strain, causing strong shear deformation at the edges [157].

The red boxed section in Figure 16 illustrates the equilibrium state at the first bonded line, where normal and shear stresses within the bonded region are balanced. This equilibrium state is crucial for understanding load transfer within the bonded area, and any disruption in this balance leads to debonding at the interface. The interfacial slip s(x) , strain ε(x) , and stress τ(x) profiles are shown on the lower half of the figure. Here, $d_{f}\left(x\right)$ represents the displacement on the surface of the FRP ties, while $d_{c}\left(x\right)$ refers to the rigid movement and displacement occurring in the concrete blocks. The strain ε(x) is defined as the first derivative of s(x) , and the shear stress τ(x) is determined by the second derivative of s(x) and incorporating the material properties of the FRP used. The shear stress τ(x) increases as interfacial slip s(x) increases along the bond length x and reaches its maximum value before the slope of s(x) reaches its peak, which indicates the onset of debonding. The fracture energy $G_{f}$ is defined as the shaded area under the bond–slip relation, representing the energy dissipated along the entire bond length during the loading process. Bond–slip relation τ(s) is derived from τ(x) and s(x) serves as a key indicator used to calculate the energy required to initiate debonding. The mathematical expressions, highlighted in purple and blue, form the basis for predicting debonding strain and failure conditions in FRP–concrete interfaces along the bonded interface. Lastly, the theoretical debonding force $P_{dep}^{theor}(s)$ shown in the black box is derived by integrating the bond–slip relation τ(s) , providing an analytical solution for predicting load–slip response under debonding with concrete fracture in EB-FRP-strengthened concrete systems [137].

**Figure 17:**
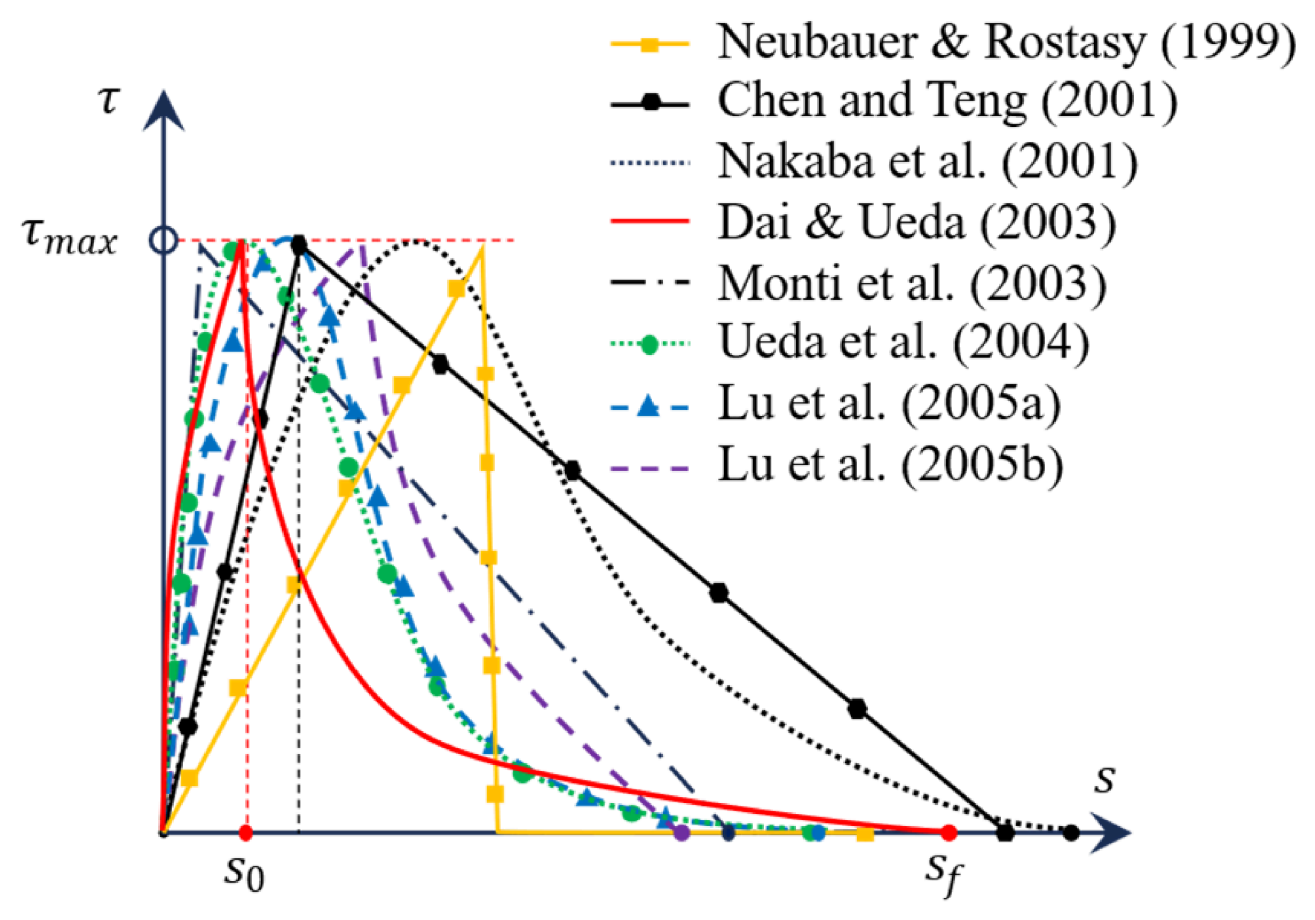
Representative bond–slip curve shapes proposed between 1999 and 2005 [76,99,158,159,160,161,163]. All curves are shape-normalized for comparison purposes, and actual peak stresses vary among models [70].

Substantial experimental, analytical, and computational research has focused on understanding the interfacial behavior between FRP and concrete over the past three decades. The development and refinement of bond–slip models have been central to advancing this understanding. The most commonly used bond–slip models are represented in Figure 17, each with different shapes aimed at capturing the critical aspects of interfacial behavior [57,76,99,158,159,160]. Bilinear models are effective for modeling brittle bond failure as they assume a steep increase in bond stress up to a peak point, followed by a linear decline with increasing slip [158,161]. Tri-linear models introduce a plateau after the peak point to capture a phase of steady stress transfer after the peak bond stress is reached [160], making them suitable for cases where slip behavior is more gradual and debonding occurs progressively. Exponential and nonlinear models represent more complex bond responses [57,159,162], where bond stress rises quickly but decays exponentially or nonlinearly, better reflecting gradual bond degradation under varying conditions. Finally, parabolic/curved models offer a smooth, continuous transition from loading to debonding [97,99], allowing for more accurate predictions of intermediate bond deterioration stages. Each of these models balances simplicity with accuracy, depending on the specific conditions of the FRP–concrete bond. Choosing the appropriate bond–slip model is crucial for predicting debonding behavior and the overall performance of EB-FRP systems, especially in real-world large-sized applications, where accurate modeling of interfacial behavior is essential.

In addition to the widely cited models discussed above, Table 15 provides an overview of 18 other models spanning from 1999 to 2025, including analytical [99,159,164,165,166], empirical [145,160,167,168], and semi-empirical [158,161,162,169] approaches. Analytical models often describe bond–slip relationships by deriving and integrating slip and bond stress profiles under Mode II fracture mechanics, which pertains to shear-dominated interfacial failure modes in FRP–concrete systems (more details below), but they tend to be complex and difficult to apply in practical design contexts. In contrast, empirical models rely on experimental data to establish direct correlations through regression and thus may lack generalizability and fail to capture the underlying mechanics of interfacial behavior. The limitations of both analytical and empirical models highlight the need for a more refined approach.

Semi-empirical models aim to bridge this gap by incorporating key parameters from both experimental findings and analytical formulations. Most existing models are based on small-scale experimental setups, using short FRP ties (median length: 200 mm), narrow thickness (median value: 0.17 mm), and a low stiffness ratio ( ${k_{f}/E}_{c}$ ) of approximately 1.4 (see Figure 15). Here, $k_{f}$ represents the stiffness of the FRP ( $NE_{f}t_{f}$ ), while $E_{c}$ is the modulus of elasticity of concrete. These conditions fail to adequately replicate real-world construction scenarios [50]. Research has shown that large-sized (thicker and longer) single-lap shear tests exhibit different characteristics compared to small-scale tests, such as a higher load-carrying capacity and longer post-debonding deformation [11,69]. As a result, this lack of validation and sufficiently accurate bond–slip models for large-sized EB-FRP systems hinders the precise prediction of load–slip responses in practical applications.

The accuracy of the empirical bond–slip models is further complicated by several factors, such as experimental conditions, material properties, and surface preparation [96]. A significant challenge in these models is that their coefficients are often calibrated empirically, without a complete understanding of their physical significance. This limitation complicates accurate predictions of debonding behavior, especially when applying the models to real-world conditions. Although experimental studies have clarified key bond indicators between FRP and concrete, achieving precise and efficient debonding load predictions for practical design purposes remains a critical focus.

A major factor contributing to the challenge above is the role of material properties in influencing bond behavior. The stiffness of FRP, which is generally much higher than that of concrete, often leads to cracking or fracture at the interface due to the low tensile strength of the concrete [57]. Proper surface preparation is also crucial to ensure effective bonding and minimize the risk of premature failure, especially under tensile loads [51,53,84,170]. However, recent research has shown that when the FRP thickness increases, resulting in stiffer FRP, concrete strength becomes less critical [11,21,69]. In such cases, the debonding load is more influenced by the stiffness of the FRP and the properties of the adhesive layer. Studies have demonstrated that bond strength can be improved with thicker, stiffer FRP and less stiff adhesives [77,151,171]. As a result, models that previously emphasized concrete strength may also need to be reconsidered when addressing large-sized EB-FRP systems.

Despite significant advancements in understanding the interfacial behavior between FRP and concrete, current bond–slip models face several limitations. Analytical models often lack practicality due to their complexity, while empirical models fail to generalize across varied configurations, as they are primarily based on small-scale tests. These models do not adequately account for the distinct characteristics of large-sized FRP systems, which are increasingly used in modern construction. Key challenges include accurately predicting debonding loads and capturing load–slip responses under realistic conditions.

**Table 15:** Existing bond–slip models from 1999 to 2025.

Bond–Slip Model	Ascending Branch $0<s<s_{0}$	Descending $s_{0}<s<s_{f}$	$\tau_{max}$ (MPa)	$s_{0}$ (mm)	$s_{f}$ (mm)	Notes
Neubauer and Rostasy (1999) [160]	$\tau_{max}\left(\frac{s}{s_{0}}\right)$	0	1.8 $\beta_{w}f_{t}$	0.224 ${\beta_{w}}^{2}$	N/A	Linear model (empirical) $\beta_{w}=\sqrt{\frac{1.125(2-b_{f}/b_{c})}{1+b_{f}/400}}$
Nakaba et al. (2001) [76]	$\tau_{max}\left(\frac{s}{s_{0}}\right)\left[\frac{3}{{2+\left(s/s_{0}\right)}^{3}}\right]$		3.5 $f_{c}^{0.19}$	0.065	N/A	Nonlinear model (empirical)
De Lorenzis et al. (2001) [172]	$\tau_{max}\left(\frac{s}{s_{0}}\right)$	$\tau_{max}\left(\frac{s_{f}-s}{{s_{f}-s}_{0}}\right)$	0.0182 $\sqrt{n_{f}t_{f}E_{f}}$	$\frac{G_{f}}{\tau_{max}}$	$\frac{2G_{f}}{\tau_{max}}$	Bilinear model (semi-empirical) $G_{f}=1.06\;N/\mathrm{mm}$
Chen and Teng (2001) [161]	$\tau_{max}\left(\frac{s}{s_{0}}\right)$	$\tau_{max}\left(1-\frac{s-s_{0}}{{s_{f}-s}_{0}}\right)$	$k\sqrt{f_{c}}$	$\frac{\tau_{max}}{k_{s}E_{f}t_{f}}$	$cs_{0}$	k , $k_{s}$ and c are empirical factors based on test materials
Monti et al. (2003) [158]	$\tau_{max}\left(\frac{s}{s_{0}}\right)$	$\tau_{max}\left(\frac{s_{f}-s}{{s_{f}-s}_{0}}\right)$	$1.8\beta_{w}f_{t}$	$2.5\tau_{max}\left(\frac{t_{a}}{E_{a}}+\frac{50}{E_{c}}\right)$	0.33 $\beta_{w}$	Bilinear model (semi-empirical) $\beta_{w}=\sqrt{\frac{1.5(2-b_{f}/b_{c})}{1+b_{f}/100}}$
Savoia et al. (2003) [167]	$\tau_{max}\left(\frac{s}{s_{0}}\right)\left[\frac{2.86}{{1.86+\left(s/s_{0}\right)}^{2.86}}\right]$		3.5 $f_{c}^{0.19}$	0.051	N/A	Nonlinear model (empirical)
Dai and Ueda (2003) [159]	$\tau_{max}{\left(\frac{s}{s_{0}}\right)}^{0.575}$	$\tau_{max}e^{-\beta \left(s-s_{0}\right)}$	$\frac{-1.575\alpha K_{a}+\sqrt{2.481\alpha^{2}{K_{a}}^{2}+6.3\alpha \beta^{2}K_{a}G_{f}}}{2\beta }$ where $K_{a}=G_{a}/t_{a}$	$\frac{\tau_{max}}{\alpha K_{a}}$ $\alpha =0.028{\left(\frac{E_{f}t_{f}}{1000}\right)}^{0.254}$	N/A	Nonlinear model (semi-empirical) $\beta =0.0035{\left(E_{f}t_{f}/1000\right)}^{0.34}$ $G_{f}=7.55{K_{a}}^{-0.449}{f_{c}}^{0.343}$
Ueda and Dai (2004) [163]	2B $G_{f}\left(e^{-Bs}-e^{-2Bs}\right)$ where $B=6.846{\left(E_{f}t_{f}\right)}^{0.108}{\left(\frac{G_{a}}{t_{a}}\right)}^{0.833}$		0.5B $G_{f}$	0.693/B	N/A	Nonlinear model (analytical) $G_{f}=0.446{\left(E_{f}t_{f}\right)}^{0.023}{\left(\frac{G_{a}}{t_{a}}\right)}^{-0.352}{f_{c}}^{0.236}$
Lu et al. precise (2005) [99]	$\tau_{max}\sqrt{\frac{s}{{As}_{0}}+B^{2}}-B$ $A=\left(s_{0}-s_{e}\right)/s_{0}$ $B=s_{e}/2\left(s_{0}-s_{e}\right)$	$\tau_{max}e^{-\alpha \left(\frac{s}{s_{0}}-1\right)}$	$\alpha_{1}\beta_{w}f_{t}$ $\alpha_{1}$ , $\alpha_{2}$ and $\alpha_{3}$ by regression	$\alpha_{2}\beta_{w}f_{t}+s_{e}$ where $s_{e}=\tau_{max}/K_{0}$ $K_{a}=G_{a}/t_{a}$ $K_{c}=G_{c}/t_{c}$ $K_{0}=K_{a}K_{c}/\left({K_{a}+K}_{c}\right)$	N/A	Nonlinear model (analytical) $\beta_{w}=\sqrt{\frac{2-b_{f}/b_{c}}{1+b_{f}/b_{c}}}$ $G_{f}=\alpha_{3}{\beta_{w}}^{2}\sqrt{f_{t}}f\left(K_{a}\right)$ $\alpha =1/\left(\frac{G_{f}}{\tau_{max}}-2/3\right)$
Lu et al. simplify. (2005) [99]	$\tau_{max}\sqrt{\frac{s}{s_{0}}}$	$\tau_{max}e^{-\alpha \left(\frac{s}{s_{0}}-1\right)}$	$\alpha_{1}\beta_{w}f_{t}$ where $\beta_{w}=\sqrt{\frac{2-b_{f}/b_{c}}{1+b_{f}/b_{c}}}$	$0.0195\beta_{w}f_{t}$	N/A	Nonlinear model $G_{f}=0.308{\beta_{w}}^{2}\sqrt{f_{t}}$ $\alpha =1/\left(\frac{G_{f}}{\tau_{max}}-2/3\right)$
Lu et al. bilinear. (2005) [99]	$\tau_{max}\left(\frac{s}{s_{0}}\right)$	$\tau_{max}\left(\frac{s_{f}-s}{{s_{f}-s}_{0}}\right)$	$\alpha_{1}\beta_{w}f_{t}$ with $\beta_{w}=\sqrt{\frac{2-b_{f}/b_{c}}{1+b_{f}/b_{c}}}$	$0.0195\beta_{w}f_{t}$	$\frac{2G_{f}}{\tau_{max}}$	Bilinear model
Pellegrino et al. (2008) [146]	$\tau_{max}\left(\frac{s}{s_{0}}\right)$	$\tau_{max}\left(\frac{s_{f}-s}{{s_{f}-s}_{0}}\right)$	3.1 ${\left(n_{f}t_{f}E_{f}\right)}^{0.32}$	0.075/ ${\left(n_{f}t_{f}E_{f}\right)}^{0.2}$	$\frac{10.5}{{\left(n_{f}t_{f}E_{f}\right)}^{0.6}}$	Bilinear model
Zhou et al. (2010) [164]	$\frac{E_{f}t_{f}}{1+\rho }\frac{\alpha }{\beta^{2}}e^{-\frac{s}{\alpha }}\left(1-e^{-\frac{s}{\alpha }}\right)$ with $\rho =\frac{E_{f}t_{f}b_{f}}{E_{c}t_{c}b_{c}}$		$\frac{E_{f}t_{f}}{4(1+\rho )}\frac{\alpha }{\beta }$	N/A	N/A	Nonlinear model (analytical) α and β were defined by regression
Baky et al. (2012) [165]	$E_{0}s+\left(\frac{\tau_{max}-E_{0}s_{0}}{{s_{0}}^{3}}\right)s^{\beta }$ $\beta =1/\left(1-\frac{\tau_{max}}{E_{0}s_{0}}\right)$	$\tau_{max}e^{-\alpha_{1}\left(\frac{s}{s_{0}}-1\right)}$ $\alpha_{1}=-0.9\tau_{max}s_{0}/G_{f}^{p}$	$\frac{2f_{t}}{-\alpha +\sqrt{\alpha^{2}+4}}$ $\alpha =\frac{\rho_{t}}{\lambda t_{f}}$ , $\lambda =\sqrt{\frac{G_{a}\left(1+\eta \rho \right)}{E_{f}t_{f}t_{a}}}$ , $\eta =\frac{E_{f}}{E_{c}}$ , $\rho =A_{f}/A_{c}$ and $A_{c}$ = $h_{eff}b_{c}$	$\tau_{max}\left(0.35\frac{t_{f}}{G_{f}}+8.5\frac{t_{a}}{G_{a}}+\frac{3h_{eff}}{G_{c}}\right)$	4 $s_{0}$	Nonlinear model (analytical) $E_{0}=1/\left(\frac{t_{f}}{G_{f}}+\frac{t_{a}}{G_{a}}+\frac{16.6}{G_{c}}\right)$ $G_{f}^{p}={\tau_{max}}^{2}\left[\frac{150}{G_{c}}-0.405\left(\frac{t_{f}}{G_{f}}+\frac{t_{a}}{4.25G_{a}}\right)\right]$
Pan and Wu (2014) [166]	ks $\left(0\leq s\leq s_{0}\right)$	$\tau_{max}e^{-\beta \left(s-s_{0}\right)}$ $\left(s\geq s_{0}\right)$	1.131 ${k_{w}}^{2}E_{d}{\left(\frac{f_{c}}{E_{d}}\right)}^{0.19}$ $E_{d}=1\;\mathrm{MPa}$ and d=1 m	$\frac{\tau_{max}h}{G}$ $G=0.247{k_{w}}^{2}E_{d}d{\left(\frac{f_{c}}{E_{d}}\right)}^{0.216}$	N/A	Nonlinear model (analytical) $k_{w}=\lambda^{\prime }+\left(1-\lambda^{\prime }\right)\cdot b_{f}/b_{c}$ $\lambda^{\prime }=1+0.222{\left(\frac{f_{co}}{E_{d}}\right)}^{0.304}$
Ko et al. (2014) [145]	$\tau_{max}\left(\frac{s}{s_{0}}\right)$	$\tau_{max}-\tau_{max}\left(\frac{s-s}{{s_{f}-s}_{0}}\right)$	0.165 $f_{c}$	−0.001 $f_{c}$ + 0.122	−0.002 $f_{c}$ + 0.302	Bilinear model (empirical)
Sun et al. (2017) [168]	$\tau_{max}\left(\frac{s}{s_{0}}\right)$	$\tau_{max}\left(\frac{s_{f}-s}{{s_{f}-s}_{0}}\right)$	$1.35+0.25{\beta_{w}f}_{t}+0.62f_{t}$ $f_{t}=0.62\sqrt{f_{c}}$	$0.016-0.0046{\beta_{w}f}_{t}+0.11\beta_{w}$	$-0.06+(0.88-0.23{\beta_{w}}^{2})\sqrt{\frac{\beta_{w}}{f_{t}}}$	Bilinear model $\beta_{w}=\sqrt{\frac{1.9-b_{f}/b_{c}}{0.9+b_{f}/b_{c}}}$
Li et al. (2022) [154]	$\tau (s)=\frac{E_{f}t_{f}}{1+\rho }\frac{\alpha }{\beta }\left\{\frac{1}{\beta }\left(\frac{e^{\frac{s}{a}}-1}{e^{2\frac{s}{a}}}\right){\left[1+A\frac{2\pi }{T}cos\left(\frac{2\pi }{T}x\right)\right]}^{2}-\left[\frac{1}{1-e^{-\frac{s}{\alpha }}}\right]A{\left(\frac{2\pi }{T}\right)}^{2}sin\left(\frac{2\pi }{T}x\right)\right\}$ where $A=k_{0}\frac{x_{0}}{L_{f}}$ , $k_{0}=\frac{1}{4\beta }{\left(\frac{T}{2\pi }\right)}^{2}$ and $T=L_{e}=2\beta \ln\left(\frac{1+\delta }{1-\delta }\right)$			N/A	N/A	Nonlinear model (analytical) $\rho =\frac{E_{f}t_{f}b_{f}}{E_{c}t_{c}b_{c}}$ $x_{0}=L_{f}+Asin\left(\frac{2\pi }{T}L_{f}\right)+\beta \ln\left(\frac{1}{1+\rho }\frac{\alpha }{\beta }\frac{E_{f}t_{f}b_{f}}{P}-1\right)$ α , β and δ were defined by regression
Lei at al. (2024) [169]	$\tau =\tau_{max}\frac{s}{s_{0}}\frac{n}{(n-1)+{\left(s/s_{0}\right)}^{n}}$ with $n=C_{1}\beta_{w}$ where $C_{1}=1.75+\frac{141.51}{4(t-15.18{)}^{2}+70.33}$	$C_{2}\beta_{w}f_{t}$		$s_{0}=C_{3}\tau_{max}\left(\frac{t}{E_{m}}+\frac{50}{E_{c}}\right)$ $C_{2}=\frac{1}{0.02(t-12.80{)}^{2}+0.45}+0.94\ln t$ $C_{3}=7.53+\frac{16.44}{1+{\left(\frac{t-11.67}{1.60}\right)}^{2}}$	N/A	Nonlinear model (semi-empirical) $\beta_{w}=\sqrt{\frac{4.63-b_{f}/b_{c}}{2.63+b_{f}/b_{c}}}$ t is the thickness of adhesive used in the tests
Zhang et al. (2025) [70]	$\tau_{1}(s)={\tau^{\prime }}_{max}{\left(\frac{s_{1}}{s_{0}}\right)}^{\alpha_{0}}$	$\tau_{2}\left(s\right)={\tau^{\prime }}_{max}e^{-\beta_{0}\left(\frac{s_{2}}{s_{0}}-1\right)}$	$1.32\beta_{w}\frac{f_{t}}{\sqrt{\ln\left(l_{b}\right)}}$	N/A	N/A	$\alpha_{0}=$ 1.9 $\;\times \;10^{-3}A\sqrt{K_{I}}$ , $\beta_{0}={{-0.41\beta }_{w}}^{2}\left({K_{I}}^{-B}+D_{I}B\right)$ , $A=\frac{s_{0}-s_{e}}{s_{0}}$ , and $B=\frac{s_{e}}{2(s_{0}-s_{e})}$

## 5. EB-FRP with Anchors (COI-B and C)

### 5.1. Overview

Studies on anchored EB-FRP systems have been systematically categorized under COI-B and COI-C. The analyses evaluating research objectives (RQ2), FRP materials, DAQs failure modes, and anchor design methodologies were reviewed. Table 16 summarizes the relevant studies and classifies them according to the COI framework.

### 5.2. Test Methods

**Figure 18:**
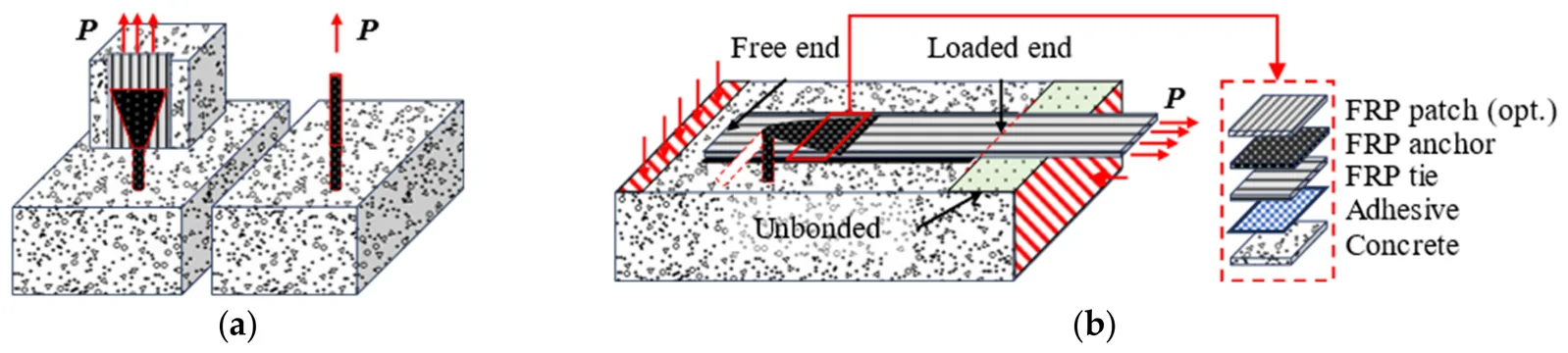
Commonly used test methods for fiber anchors: (**a**) direct tension test (straight anchor) and (**b**) lap shear tests (bent anchor). Red arrows indicate the loading direction, hatched regions denote the bonded or stress-transfer zones, and the different shading patterns identify the constituent materials and bonded interfaces.

Two primary approaches are employed to evaluate fiber anchors in EB-FRP systems: direct tension tests and lap shear tests. Direct tension tests are primarily used for straight anchors and typically focus on isolated anchor behavior, as shown in Figure 18a, often implemented through pullout tests that apply load either to the FRP sheet beneath the anchor fan or directly to the anchor dowel region. Single-lap and double-lap shear tests, shown in Figure 18b, are better suited for bent anchors and are widely applied to assess anchored EB-FRP systems classified under COI-B2. These tests can also evaluate isolated anchors (COI-B1) when an unbonded layer, such as a thin plastic sheet, is introduced between FRP and concrete to eliminate unintended interfacial bonding effects. Both approaches are effective for quantifying anchor capacity, with selection determined by the strengthening configuration. COI-B and COI-C studies encompass results from both test types, focusing on single-anchor configurations relevant to tension member strengthening, where anchors transfer loads from FRP ties into concrete to maintain continuity of the load path.

### 5.3. Failure Patterns

**Figure 19:**
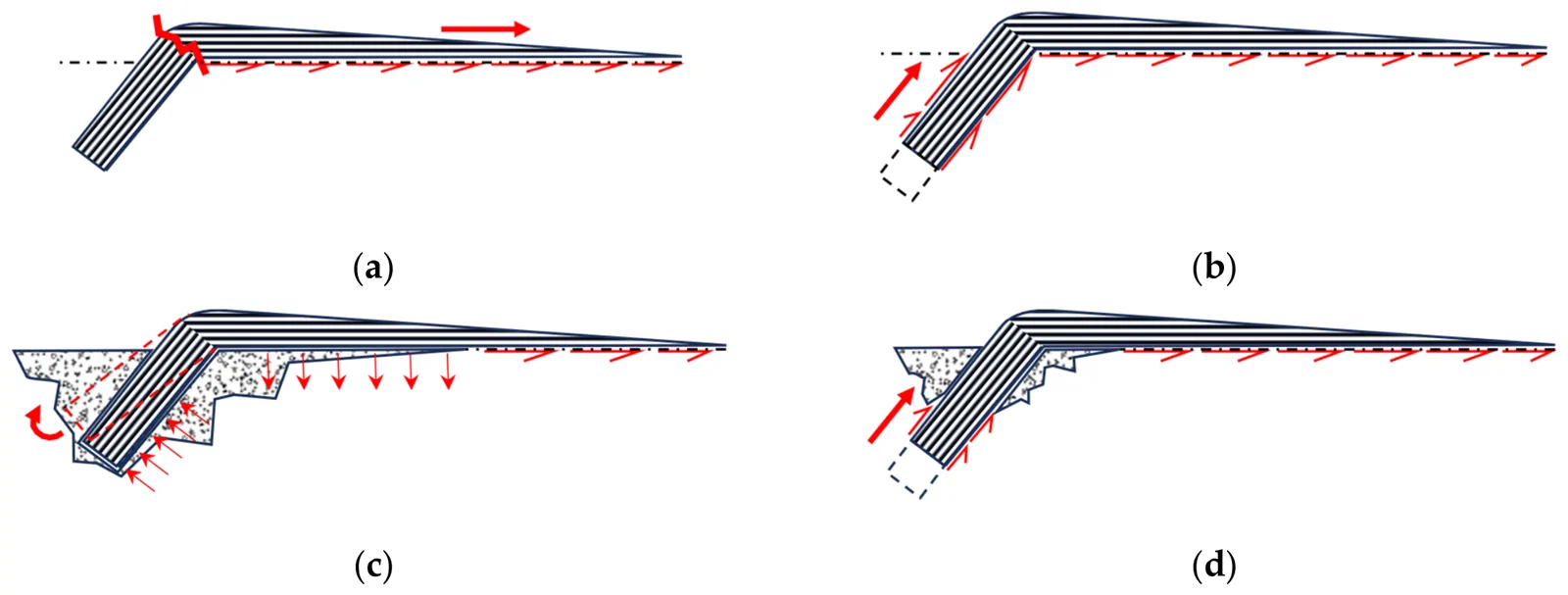
Observed failure modes (e.g., insertion angle with 135-degree): (**a**) anchor rupture, (**b**) anchor pullout, (**c**) concrete pryout, and (**d**) bond mixed (pullout with concrete pryout) [191]. Red arrows indicate load transfer and failure directions, curved arrows illustrate local rotational mechanisms, dashed lines represent failure planes or original anchor positions, and the hatched regions denote damaged or debonded zones.

The failure patterns of fiber anchor under tension can be categorized into rupture, pullout, concrete-related failures, and bond-mixed types [37,38,188], as shown in Figure 19. Rupture failures occur under high tensile stresses and are governed by the tensile strength of the FRP material (Figure 19a). Pullout failures are primarily influenced by dowel geometry, including embedment depth and dowel diameter, as well as the stress distribution within the substrate (Figure 19b). Concrete-related failure modes, such as concrete pryout failure and bond-mixed failure, involve additional mechanisms, including substrate detachment and the interaction between bond–shear stress and concrete strength (Figure 19c,d). Among these, rupture failures are more predictable, governed by the well-characterized tensile strength of the FRP material, with research supporting reliable predictive models and design practices [44,199]. In contrast, pullout failure modes are particularly challenging to predict due to difficulties in defining criteria for pure pullout behavior. Specimens with some concrete attached to the dowel may blur the lines between pullout, concrete pryout, or mixed concrete pryout-bond failure modes.

Deeper anchors are generally preferred for achieving reliable performance, but shallow anchors are often required due to practical constraints, such as thin slabs or geometric limitations. In shallow embedment configurations, pullout failure becomes more prevalent, characterized by mechanisms involving partial substrate detachment and bond–shear interactions. Beyond embedment depth, the manufacturing process of fiber anchors also introduces variability in their structural performance. Handmade anchors, often produced with non-standardized techniques, exhibit inconsistencies in geometry, fiber distribution, and adhesive application, which can significantly affect their mechanical behavior. In contrast, precured anchors offer improved uniformity and reliability due to controlled production processes. However, the impact of these differences on anchor performance, particularly under shear conditions and in shallow embedment scenarios, has not been systematically investigated. Addressing this gap is crucial for developing robust predictive models and ensuring the reliability of EB-FRP systems in structural applications.

### 5.4. COI-B and C Dataset

#### 5.4.1. Collected Test Results

A total of 650 test results under COI-B were compiled [42,200]. The dataset was assembled from experimental studies [30,31,32,35,36,38,39,40,46,153,170,173,180,191,192,193,194,195,201,202,203,204] and includes parameters such as specimen geometry, FRP anchor configuration (i.e., insertion angle, fan angle, dowel diameter, fan length, and embedment depth), material properties (i.e., concrete compressive strength and FRP tensile and elastic moduli), and recorded ultimate capacity. Most investigations focused on isolated anchor tests rather than full-scale anchored EB-FRP systems. Under COI-C, only 96 test results were reported from four published studies [46,196,205,206]. The scarcity of large-scale experimental data continues to limit the development and validation of design models for both single- and multiple-anchored EB-FRP configurations intended for field applications.

For anchored systems, the evidence supports a qualitative hierarchy of what each configuration can show. Isolated straight-anchor pullout tests are useful for dowel embedment, diameter, and concrete-related failure limits, but they do not quantify the combined plate–anchor load path. Bent-anchor lap-shear tests better represent externally bonded systems because they include fan-to-strip force transfer, yet results are sensitive to fan geometry, insertion angle, embedment depth, and whether the anchor is handmade or precured. Single-anchor EB-FRP tests can evaluate local anchorage effectiveness and debonding delay, whereas COI-C multi-anchor tests are needed to assess anchor spacing, load sharing, and system capacity. A review of the available studies indicates that reported capacity increases vary widely because the reference configurations differ in bonded length, FRP stiffness, anchor geometry, specimen size, loading arrangement, and governing failure mode. Consequently, the literature does not currently support a single representative capacity-increase range that can be applied across different anchor configurations. From a design perspective, anchor configuration should be selected together with the target failure mode: rupture-governed anchors are more predictable, while shallow pullout, concrete pryout, and mixed failures require conservative detailing and further validation. The principal anchor configurations, their experimental objectives, practical advantages, and limitations are summarized in Table 17.

#### 5.4.2. Existing Strength Models

Model accuracy was assessed qualitatively by comparing each model’s validation scope, governing failure mode, required input variables, and transferability to configurations outside its calibration set. Models based on FRP rupture are generally more stable when material tensile strength and anchor cross-section are well defined, whereas pullout and concrete-related models are more sensitive to embedment depth, dowel diameter, substrate strength, and boundary conditions. Empirical and semi-empirical equations are useful inside their reported parameter ranges but can become unreliable for shallow embedment, bent anchors, handmade-anchor variability, and multi-anchor systems. The available manuscript-level dataset does not provide enough consistently reported predicted-to-tested ratios, mean error, coefficient of variation, or RMSE values to recalculate a uniform numerical accuracy ranking across all 42 formulations. Accordingly, the comparison is restricted to transparent validity domains and the need for future benchmark datasets with common error metrics. The available evidence for model assessment, applicable validity domains, and key limitations are summarized in Table 18.

Building on this comparison framework, the models summarized in Table 19 span various failure modes and anchor types (straight and bent) developed between 1995 and 2026. Strength models typically define anchor capacity as a function of the fiber-reinforced polymer (FRP) material’s tensile strength ( $f_{f}$ ), and the geometry of the anchor, including fiber cross-section ( $A_{d}$ ), embedment depth ( $h_{ef}$ ), insertion angle ( β ), and fan angle ( α ) [37,38,39,178,179]. Concrete-related failure models emphasize geometric parameters such as embedment depth, cone angle, and dowel diameter, and their effectiveness depends on sufficient anchorage depth for cone development [37,181,182,183]. Mixed concrete pryout–pullout failure models incorporate bond–shear interactions and dowel–concrete interface stresses, with varying levels of accuracy across different embedment depths and loading conditions [36,37,38,187,188,189,190]. Fan debond models primarily investigate the detachment of the FRP fan component and its contribution to overall anchor behavior [174,175,176]. Despite these advancements, existing models inadequately address anchor pullout under lap-shear conditions, particularly in shallow embedment and bent-configured scenarios such as thin toppings on hollow-core slabs. The variability of manufacturing processes, specifically the differences between handmade and precured anchors, remains insufficiently studied. Additional predictive models for ultimate capacity of anchored EB-FRP systems, summarized in Table 20, incorporate both FRP strip debonding behavior and anchor contribution to overall capacity [30,40,180,196]. But interaction between multiple anchors, particularly when anchors are arranged in varied alignments, remains poorly understood. The complexity of load transfer in such configurations creates uncertainty in ultimate capacity prediction and highlights the need for further investigation to establish reliable design approaches.

## 6. Future Research Directions

This systematic review identifies significant research gaps in EB-FRP strengthening systems, particularly in anchored configurations. Research on unanchored systems (COI-A) dominates the literature and represents 3162 of the 3908 compiled test results (80.9%). Nearly all tests were performed on small-scale specimens with short or thin bond lengths and simplified boundary conditions, limiting their applicability to field-scale behavior. Anchored systems (COI-B and COI-C) remain poorly explored, with 746 test results available, mostly from small-scale isolated anchor tests, and just 96 tests specifically on anchored EB-FRP configurations. The lack of large-scale data for both unanchored and anchored systems restricts predictive model calibration and delays the development of reliable design provisions for practical applications. Existing strength and bond–slip models are largely empirical, derived from these small-scale datasets, and do not adequately capture anchor–strip interactions or multi-anchor load-sharing behavior. Variability in anchor fabrication, particularly between handmade and precured anchors, adds further uncertainty. Current design standards, including ACI PRC-440.2 (2023) [207], CNR-DT 200 (2025) [198], and fib Bulletin 90 (2019) [49], provide minimal guidance on anchored systems and omit durability and complex loading considerations. Addressing these limitations should focus on three research domains: expanding experimental scope, advancing data acquisition strategies, and developing theoretical models with unified design guidelines. The resulting evidence-based research agenda is summarized in Table 21, where priorities are ranked according to their direct connection to the review findings. Highest priority is assigned to gaps that currently prevent normalized comparison, reliable model validation, or practical design implementation.

### 6.1. Expansion of Research Scope

Future research on EB-FRP systems should extend beyond the small-scale testing that dominates current datasets. Experimental programs need to address full-scale members, realistic anchorage layouts, and combined loading conditions to reflect structural behavior in practice. Priority areas include systematic evaluation of anchor layout optimization, multi-anchor interaction effects, and the role of load transfer paths in overall system performance. Long-term performance under environmental exposure, fatigue, and dynamic actions also requires dedicated investigation to ensure durability and reliability in field applications.

### 6.2. Advancement of DAQs

Current data acquisition methods rely heavily on strain gauges and LVDTs, which provide localized measurements but do not capture distributed bond–slip or strain fields. Wider adoption of non-contact techniques such as 2D/3D digital image correlation and fiber optic sensing should be pursued to obtain full-field data and enable real-time monitoring of crack initiation and propagation. Integrating these advanced methods with conventional sensors would provide hybrid systems capable of high spatial resolution and temporal accuracy. Establishing standardized protocols for data collection and processing should enhance cross-study comparability and support validation of analytical and numerical models.

### 6.3. Development of Theoretical Models and Design Guidelines

Current models for bond–slip behavior and anchor strength are largely empirical and calibrated to small-scale tests with limited parameter ranges. Such models do not sufficiently represent anchor–strip interactions, multi-anchor effects, or complex load transfer observed in field applications. Future modeling efforts should integrate mechanics-based principles to better capture debonding and failure mechanisms, while also incorporating variability in material properties and strip/anchor manufacturing methods. Developing semi-empirical models that combine mechanistic accuracy with design simplicity would support practical implementation in engineering design guidelines. Unified design guidelines capable of addressing both unanchored and anchored EB-FRP systems, including multiple-anchor configurations and durability considerations, are essential to ensure consistent and reliable application in structural rehabilitation.

## 7. Conclusions

This systematic review synthesized 174 experimental studies published between 1994 and 2026, comprising 3908 test results and 42 analytical formulations addressing unanchored and anchored EB-FRP systems for concrete structures. Experimental evidence, bond behavior, anchorage performance, and strength formulations were evaluated using the COI framework to identify governing mechanisms, limitations of current knowledge, and priorities for future research. The following conclusions can be drawn:

▪ Bond performance in unanchored EB-FRP systems is governed primarily by FRP stiffness, strip geometry, bond length, concrete strength, adhesive properties, surface preparation, and environmental exposure. Ultimate capacity, capacity per bonded width, debonding strain, effective bond length, fracture energy, load-slip response, and failure mode provide the most appropriate basis for comparing bond behavior across experimental studies.
▪ Anchored EB-FRP systems enhance force transfer, delay premature debonding, and improve load-carrying capacity relative to unanchored configurations. Available evidence demonstrates the effectiveness of fiber anchors for improving anchorage performance. Limited understanding remains regarding anchor–strip interaction, anchor spacing effects, load redistribution mechanisms, and multi-anchor system behavior under realistic structural conditions.
▪ Unanchored systems dominate the current evidence base, contributing 3162 of the 3908 compiled test results, representing 80.9% of all experimental data. Anchored configurations contribute 650 COI-B test results and only 96 COI-C multi-anchor system test results. Current understanding of system-level anchorage behavior, therefore remains substantially less developed than understanding of basic bond mechanisms.
▪ CFRP dominates the reviewed literature, appearing in 143 studies, whereas GFRP, BFRP, and AFRP appear in 23, 18, and seven studies, respectively. General conclusions regarding FRP behavior should therefore be interpreted primarily as CFRP-based unless direct comparisons among fiber types are available.
▪ Existing bond–slip and anchor-capacity models capture specific failure mechanisms within calibration ranges but remain predominantly empirical or semi-empirical. Limited transferability across different geometries, loading conditions, anchorage configurations, and environmental exposures restricts broader application. Consistent reporting of predicted-to-tested ratios, coefficients of variation, and other common accuracy metrics remains necessary to support future benchmarking and model validation.
▪ Durability effects and field implementation remain major challenges. Elevated temperature, moisture exposure, wet–dry cycling, freeze–thaw action, fatigue loading, and long-term aging can alter adhesive-dependent load transfer and failure behavior. Available exposure protocols and reporting methods remain too heterogeneous to support reliable generalized degradation relationships.
▪ Future research should prioritize normalized cross-study databases, large-scale and field-representative testing programs, full-field measurement techniques, durability-sensitive design approaches, multi-anchor interaction studies, experimentally validated numerical models, and unified design provisions for unanchored and anchored EB-FRP strengthening systems.

## Figures and Tables

**Table 1:** Stages and results of initial search.

	Database	Scopus	Web of Science
Stages			
**General** **requirements**	Scope	Article title, abstract, keywords	
	Pub. year	From all years to 17 February 2026	
	Doc. type	Journal articles and Review papers	
	Language	English	
**Stage 1** (+) “General topic”	Boolean	*TITLE-ABS-KEY ((“FRP” OR “fiber reinforced polymer”) AND (concrete OR “reinforced concrete” OR RC))*	*TS = ((“FRP” OR “fiber reinforced polymer”) AND (concrete OR “reinforced concrete” OR RC))*
	Results	24,069	17,529
**Stage 2** (+) “Add EB-FRP focus”	Boolean	Previous result *AND TITLE-ABS-KEY (“externally bonded” OR EB-FRP OR “bonded reinforcement”)*	Previous result *AND TS = (“externally bonded” OR EB-FRP OR “bonded reinforcement”)*
	Results	2793	2035
**Stage 3** (+) “Filter test configurations”	Boolean	Previous result AND TITLE-ABS-KEY (“pullout test” OR “lap shear” OR “direct tension” OR “bond-slip” OR “load-slip” OR debond*)	Previous result AND TS = (“pullout test” OR “lap shear” OR “direct tension” OR “bond-slip” OR “load-slip” OR debond*)
	Results	1100	878
**Stage 4** (+) “Add experimental scope”	Boolean	Previous result AND TITLE-ABS-KEY (experiment* OR test* OR “bond behavior” OR “interfacial behavior” OR “failure mode” OR “bond strength”)	Previous result AND TS = (experiment* OR test* OR “bond behavior” OR “interfacial behavior” OR “failure mode” OR “bond strength”)
	Results	1022	811
**Stage 5** (+) “material & fiber anchor response specificity”	Boolean	Previous result AND TITLE-ABS-KEY (“CFRP” OR “carbon fiber” OR “strain profile” OR DIC OR “fan angle” OR “embedment depth” OR “FRP anchor*” OR “spike anchor*” OR “FRP tie*”)	Previous result AND TS = (“CFRP” OR “carbon fiber” OR “strain profile” OR DIC OR “fan angle” OR “embedment depth” OR “FRP anchor*” OR “spike anchor*” OR “FRP tie*”)
	Results	584	478
**Stage 6** (−) “Exclude irrelevant topics”	Boolean	Previous result AND NOT TITLE-ABS-KEY (NSM OR “near surface mounted” OR “machine learn*” OR “deep learn*” OR algorithm* OR simulat* OR “finite ele*”)	Previous result NOT TS = (NSM OR “near surface mounted” OR “machine learn*” OR “deep learn*” OR algorithm* OR simulat* OR “finite ele*”)
	Results	412	321
**Stage 7** (−) “Final language and type filter”	Boolean	Previous result *AND (LIMIT-TO (DOCTYPE,”ar”) OR LIMIT-TO (DOCTYPE,”re”)) AND (LIMIT-TO (LANGUAGE, “English”))*	Previous result *AND (DT == (“ARTICLE” OR “REVIEW”) AND LA == (“ENGLISH”))*
	Results	274	287
**Result after deduplication**		**334**	

**Note:** * denotes a wildcard operator in Scopus and Web of Science used to capture multiple word variations.

**Table 2:** Keyword search robustness check.

Alternative Term Family	Quantitative Examples from the Final Included Studies (N = 174)	How It Is Covered in the Search/Coding Workflow	Residual Limitation
Bonded plate, bonded sheet, externally bonded reinforcement	Bonded plate: 5 studies (2.9%); bonded sheet: 1 study (0.6%); externally bonded reinforcement: 7 studies (4.0%).	Covered by broad FRP–concrete and externally bonded/bonded reinforcement terms, then checked through title, abstract, keywords, and citation tracking.	Exact database hit counts for these alternative terms were not recalculated after screening; the included-study counts indicate low residual risk.
GFRP, glass fiber, BFRP, basalt, AFRP, aramid	GFRP: 23 studies (13.2%); BFRP: 18 studies (10.3%); AFRP: 7 studies (4.0%). CFRP remained dominant with 143 studies (82.2%).	Early broad FRP terms and citation tracking were intended to avoid premature CFRP-only filtering; retained material types were then coded separately.	The final specificity stage visibly emphasizes CFRP-related terms, so material-recall bias is acknowledged as a limitation.
Spike anchor, fiber anchor, FRP tie, mechanical anchorage	Spike anchor: 6 studies (3.4%); fiber/FRP anchor: 24 title-level studies (13.8%) and 33 anchored-study category papers (19.0%); mechanical anchorage: 1 study (0.6%).	Anchor synonyms are included in the final strings and checked during full-text eligibility.	Terminology varies across regions and older papers; citation tracking remains important.
Pull-off, pullout, single-lap shear, lap shear	Pull-off: 1 study (0.6%); pullout: 3 title-level studies (1.7%) and 12 coded pullout-test papers (6.9%); single-lap shear: 123 coded papers (70.7%).	Pullout/lap-shear/direct-tension/bond–slip terms capture the main experimental configurations.	Some structural-member tests may be excluded if they did not report relevant bond behavior; the exact phrase single-lap shear was represented through broader lap-shear coding.

**Table 3:** Criteria of screening.

Criteria	Exclusion	Inclusion
Type of Research	Non-journal publications such as conference proceedings, book chapters, or editorial notes.	Journal articles or review papers written in English and published in final, peer-reviewed form.
Relevance	1. Studies involving numerical models, simulations, or finite element methods. 2. Alternative strengthening systems such as near-surface mounted (NSM), mechanically fastened (MF), hybrid bonding (HB), or grooving methods (GM). 3. Applications involving different materials, including masonry, timber, UHPC, recycled concrete, or FRCM systems. 4. Other test types not directly related to bond behavior, such as beam bending, compression, impact, or freeze–thaw tests	Studies regarding: 1. Experimental studies on EB-FRP strengthening of concrete elements subjected to direct tension or lap-shear loading. 2. Focus on interfacial bond behavior between FRP and concrete, including systems with and without FRP anchors.

**Table 4:** Results of screening and reasons for exclusion.

Exclusion Category	1st Round (Title and Keyword)	2nd Round (Abstract)
Numerical modeling and monitoring methods: *(Numerical analysis, FEM, Bayesian inference, machine learning, support vector machine, neural networks, and SHM)*	15	8
Alternative strengthening strategies: *(NSM, hybrid anchors, lap-spliced bars, mechanical anchors)*	23	3
Incompatible or non-concrete materials *(Masonry/timber strengthened by FRP/Steel/FRCM, UHPC, recycled concrete, FRP coupon tests)*	21	3
Non-bond-specific structural tests: *(Case study, compression test on concrete column and concrete-filled steel tubular, punching test on RC slab-column joints and web opening slab, FRP coupon tests)*	6	0
Irrelevant test types: *(Push-out, freeze–thaw, impact tests, three/four-point monotonic bending, compression tests, cantilever bending tests)*	16	39
Other types of Documents *(Books or Conference proceeding)*	6	1
**Total excluded papers**	87	54
**Articles remaining after screening**	247	193

**Table 5:** Recommended included items for current systematic literature review based on PRISMA Checklist (2020) [63].

Section and Topic	Items No.	Checklist Item	Location Where Item Is Reported
**TITLE**			
Title	1	Identify the report as a systematic review.	Introduction
**ABSTRACT**			
Structured summary	2	Provide a structured summary including, as applicable: background; objectives; data sources; study eligibility criteria, synthesis methods; results; limitations; conclusions and implications of key findings.	Introduction
**INTRODUCTION**			
Rationale	3	Describe the rationale for the review in the context of existing knowledge.	Introduction
Objectives	4	Provide an explicit statement of the research objective(s) or question(s) the review addresses.	
**METHODS**			
Eligibility criteria	5	Specify the inclusion and exclusion criteria for the review and how studies were grouped for the syntheses.	Methodology
Information sources	6	Specify all databases, registers, websites, organizations, reference lists and other sources searched or consulted to identify studies. Specify the date when each source was last searched or consulted.	
Search strategy	7	Present the full search strategies for all databases, registers and websites, including any filters and limits used.	
Selection process	8	Specify the methods used to decide whether a study met the inclusion criteria of the review, including how many reviewers screened each record and each report retrieved, whether they worked independently, and if applicable, details of automation tools used in the process.	
Data collection process	9	Specify the methods used to collect data from reports, including how many reviewers collected data from each report, whether they worked independently, any processes for obtaining or confirming data from study investigators, and if applicable, details of automation tools used in the process.	
Data items	10a	List and define all outcomes for which data were sought. Specify whether all results that were compatible with each outcome domain in each study were sought (e.g., for all measures, time points, analyses), and if not, the methods used to decide which results to collect.	
	10b	List and define all other variables for which data were sought (e.g., participant and intervention characteristics, funding sources). Describe any assumptions made about any missing or unclear information.	
Study risk of bias assessment	11	Specify the methods used to assess risk of bias in the included studies, including details of the tool(s) used, how many reviewers assessed each study and whether they worked independently, and if applicable, details of automation tools used in the process.	
**RESULTS**			
Study selection	16a	Describe the results of the search and selection process, from the number of records identified in the search to the number of studies included in the review, ideally using a flow diagram.	Methodology
	16b	Cite studies that might appear to meet the inclusion criteria, but which were excluded, and explain why they were excluded.	
Study characteristics	17	Cite each included study and present its characteristics.	Bibliometric Analysis and Research Landscape
Results of syntheses	20b	Present results of all statistical syntheses conducted. If meta-analysis was done, present for each the summary estimate and its precision (e.g., confidence/credible interval) and measures of statistical heterogeneity. If comparing groups, describe the direction of the effect.	
**DISCUSSION**			
Discussion	23a	Provide a general interpretation of the results in the context of other evidence.	COI-A, B and C
	23b	Discuss any limitations of the evidence included in the review.	
	23d	Discuss implications of the results for practice, policy, and future research.	Future Directions
**OTHER INFORMATION**			
Support	25	Describe sources of financial or non-financial support for the review, and the role of the funders or sponsors in the review.	Funding Sources
Availability of data, code and other materials	27	Report which of the following are publicly available and where they can be found; template data collection forms; data extracted from included studies; data used for all analyses; analytic code; any other materials used in the review.	Data Availability

**Table 6:** The developed thematic template based on the modified PRISMA.

Analysis Topic	Descriptions
*General information*	Title, authors, journal, year, and country. Data recorded for bibliometric profiling and publication trend analysis.
*Study focuses and objectives*	Research aims and scopes, such as bond performance evaluation, anchor detailing, or interfacial stress characterization.
*DAQ strategies and failure modes*	Instrumentation methods (e.g., strain gauges, LVDTs, DIC, and fiber optics) and reported failure types (e.g., cover separation, debonding, and FRP rupture).
*RQ1 & RQ2—Bond behavior and anchor configuration*	Experimental outcomes on effective bond length, strain distribution, anchorage geometry, and the effect of single vs. multiple anchors.
*Anchor strength modeling and validation*	Collection and comparison of analytical, empirical, or semi-empirical models for predicting anchorage strength under different failure modes. Evaluation of model performance based on experimental data with and without anchors.
*RQ3—Future research directions*	Identified gaps, limitations, and suggestions for further exploration, such as prevailing DAQs, hybrid anchoring, or modified bonding surfaces.
*Other relevant information*	Any additional observations relevant to EB-FRP interfacial performance that extend beyond the core themes.

**Table 7:** Comparability and normalization audit.

Comparison Target	Preferred Extracted/Derived Metric	When Direct Comparison Was Allowed	When Only Stratified Interpretation Was Used
Unanchored capacity	Ultimate load and capacity per bonded FRP width.	Compatible geometry, loading scheme, and failure-mode reporting.	Missing bonded width, incompatible boundary conditions, or mixed failure definitions.
Debonding behavior	Debonding strain, strain profile, effective bond length, load–slip or bond–slip response.	Reported strain or slip fields and identifiable bond length.	Only peak load reported or strain location not specified.
Scale transfer	Bond length/effective bond length, FRP stiffness ratio, specimen dimensions.	Comparable specimen geometry and loading setup.	Coupon and member tests mixed without consistent geometry or boundary data.
Anchor contribution	Normalized anchor area, embedment-depth/dowel-diameter ratio, anchor count/spacing, failure mode.	Same anchor family and comparable control specimens.	Different reference specimens, handmade/precured anchor differences, or system tests mixed with isolated pullout tests.
Model validation	Published predicted/tested ratios, mean error, COV, RMSE, or stated validation domain.	Common error metric or common validation dataset reported.	Equations listed without comparable error statistics.
**Research Question**	**Coded Evidence**	**Comparison Criteria**	**Synthesis Output**
RQ1: unanchored EB-FRP	Ultimate load, capacity per width, strain/debonding strain, effective bond length, load–slip or bond–slip response, fracture energy, failure mode.	FRP type, stiffness ratio, bond length, concrete strength, adhesive/surface preparation, exposure condition, test method and specimen scale.	Parameter-effect trends and limits of direct comparability.
RQ2: anchored EB-FRP	Anchor type, dowel/fan geometry, embedment, insertion/fan angle, anchor count/spacing, ultimate capacity, failure mode.	Isolated versus system tests, straight versus bent anchors, pullout/direct tension/lap shear methods, single versus multiple anchors.	Anchor effectiveness mechanisms and model-validity limits.
RQ3: gaps and design needs	Missing variables, small-scale dominance, underrepresented FRP types, model validation, durability and field transfer.	Evidence coverage and consistency across COI levels.	Prioritized research directions and design implications.

**Table 8:** Complexity of Investigation (COI) classification matrix.

COI Level	Objectives	DAQs	Models	Failure Modes
**CoI-C** **(Advanced)**	Studied multi-anchored EB-FRP systems	Advanced systems (DIC, multi-point strain gauges)	Developed models, validated against existing works	Analyzed with detailed mechanical explanation
**CoI-B** **(Intermediate)**	Investigated single-anchored EB-FRP or isolated anchors	Standard tools (strain gauges, LVDTs)	Applied or adapted models with limited validation	Analyzed qualitatively
**CoI-A** **(Basic)**	Focused on unanchored FRP under pure tension	Minimal DAQs (load–displacement only)	No model development or validation	Described without mechanical interpretation

**Table 9:** Preliminary regional research trends.

Leading Country/Cluster	Records (N = 174)	Observed Emphasis in Retained Studies	Predominant Trial or Analysis Type	Interpretation Limit
China/broader East Asian cluster	26.0%	Large contribution to FRP–concrete bond, bond–slip, plate/sheet interface, and code-oriented studies.	Single-lap or double-lap shear tests, bond–slip modeling, and FRP interface studies.	Country labels reflect first-author affiliation and collaboration clustering. Individual studies remain heterogeneous.
Italy/European cluster	18.1%	Contributions to design guidance, interface mechanics, and durability-related research.	EBR interface tests, analytical modeling, and guideline-oriented interpretation.	Regional attribution remains approximate because collaboration networks overlap.
United States/North American cluster	17.7%	Greater emphasis on structural strengthening applications and design-oriented investigations.	Anchored systems, member-level strengthening tests, and code-related studies.	Results describe broad trends rather than national research schools.
Iran	13.0%	Frequent focus on CFRP–concrete bond behavior, environmental effects, and parameter studies.	Lap-shear bond tests and CFRP sheet parameter investigations.	Several studies originate from closely related research groups.
Australia/New Zealand	5.8%	Contributions to EBR systems, interface behavior, and strengthening applications.	Bond behavior studies and structural strengthening.	Small sample size limits detailed interpretation.

**Table 10:** Distribution of the included articles among different journals and relevant SJR (updated to 17 February 2026).

No.	Journal Names	Publisher	SJR	H-Index	IF	1994–2002	2003–2007	2008–2012	2013–2017	2018–2022	2023–2026	Papers in Total
1	*Composites Part B: Engineering*	Elsevier Ltd.	Q1	227	14.2	1	2	4	6	5	3	21
2	*Cement and Concrete Composites*	Elsevier Ltd.	Q1	212	13.1	-	-	-	1	-	-	1
3	*Construction and Building Materials*	Elsevier Ltd.	Q1	259	8.0	-	1	1	2	11	10	25
4	*Composite Structures*	Elsevier BV	Q1	213	7.1	1	-	2	4	10	10	27
5	*Structures*	Elsevier Ltd.	Q1	70	4.3	-	-	-	-	1	3	4
6	*Engineering Structures*	Elsevier BV	Q1	205	6.4	-	-	2	4	3	3	12
7	*Engineering Fracture Mechanics*	Elsevier BV	Q1	166	5.3	-	1	1	1	1	1	5
8	*Polymers*	MDPI AG	Q1	167	4.9	-	-	-	-	1	2	3
9	*Journal of Composites for Construction*	ASCE	Q1	130	2.9	2	3	6	6	8	9	34
10	*International Journal of Solids and Structures*	Elsevier Ltd.	Q1	207	3.8	-	-	-	-	1	1	2
11	*Journal of Reinforced Plastics and Composites*	SAGE Publications Ltd.	Q1	94	2.2	-	-	-	3	-	-	3
12	*Journal of Materials in Civil Engineering*	ASCE	Q1	149	3.1	-	-	-	1	-	-	1
13	*Materials and Structures*	Springer Netherlands	Q1	147	3.4	-	-	-	1	-	-	1
14	*IJCSM*	Springer Science	Q1	55	3.8	-	-	-	1	-	-	1
15	*International Journal of Adhesion and Adhesives*	Elsevier Ltd.	Q1	117	3.5	-	-	-	1	2	-	3
16	*Structural Concrete*	Wiley-Blackwell	Q1	63	3.3	-	-	-	1	-	-	1
17	*Arabian Journal for Science and Engineering*	Springer Berlin	Q1	81	2.6	-	-	-	1	-	-	1
18	*Journal of Engineering Mechanics*	ASCE	Q1	149	3.3	-	1	-	-	-	-	1
19	*Case Studies in Construction Materials*	Elsevier Ltd.	Q1	77	6.6	-	-	1	1	2	3	7
20	*Journal of Structural Engineering*	ASCE	Q1	189	3.7	-	-		1	-	1	2
21	*Advances in Structural Engineering*	SAGE Publications Inc.	Q1	62	2.6	-	-	1	-	-	-	1
**All Q1 journals by SCImago journal rank (21 listed journals)**						**4**	**8**	**18**	**35**	**45**	**46**	**156**
22	*Materials*	MDPI AG	Q2	191	3.2	-	-	-	-	1	1	2
23	*Magazine of Concrete Research*	ICE Publishing	Q2	80	2.7	-	-	-	2	-	-	2
24	*International Journal of Polymer Science*	Hindawi Limited	Q2	65	3.4	-	-	-	-	2	1	2
25	*ACI Structural Journal*	ACI	Q2	142	1.7	2	-	1	1	-	1	10
26	*Journal of Composite Materials*	SAGE Publications Ltd.	Q2	115	2.4	-	-	-	1	-	1	1
**All Q2 journals by SCImago journal rank (5 listed journals)**						**2**	**0**	**1**	**4**	**3**	**4**	**14**
27	*Canadian Journal of Civil Engineering*	NRCCanada	Q3	78	1.1	-	-	-	1	1	-	2
28	*Journal of Testing and Evaluation*	ASTM	Q3	50	0.8	-	-	-	1	-	-	1
29	*Australian Journal of Structural Engineering*	Taylor and Francis Ltd.	Q3	26	0.9	-	-	-	1	-	-	1
**All Q3 journals by SCImago journal rank (3 listed journals)**						**0**	**0**	**0**	**3**	**1**	**0**	**4**
**Total number of papers**						**6**	**8**	**19**	**42**	**49**	**50**	**174**

**Table 11:** FRP material comparison.

FRP Type	Representation in Reviewed Papers	Interpretive Confidence	Main Synthesis Implication
CFRP	143 studies	High	Most general FRP conclusions are CFRP-weighted; higher stiffness can increase load-transfer demand and interfacial stress concentration.
GFRP	23 studies	Moderate to limited	Useful for lower-modulus comparisons and lower cost, but fewer anchored-system validations limit generalization.
BFRP	18 studies	Limited	Evidence supports inclusion as a distinct material family, but trends remain sensitive to geometry, adhesive, and concrete properties.
AFRP	7 studies	Very limited	High toughness and fatigue resistance; insufficient for broad independent conclusions

**Table 12:** Comprehensive categorization of reviewed papers at the COI-A level.

Level of COI	Objectives	Tests 1	FRPs 2	Data acquisition Strategies 3	Failure Modes Observed 4	Theory Development	Included Papers
**COI-A**	Effects of bonding adhesives	S/D	AFRP BFRP CFRP	2D-DIC & SGs 2D-DIC & πGs LVDTs & SGs	Concrete failure FRP strips Debonding (FA/CA/DC)	Validated guidelines (ACI/fib/HB/CNR) Bond–slip models	[57,58,59,60]
	Effects of aggregates/ volume fraction	S	BFRP CFRP	PIV-DIC 2D-DIC & SGs LVDTs & SGs	Strips debondin (CA/DC) Strips rupture	Bond strength models Bond–slip models Effective bond length	[71,72,73,74,75]
	Size effects (Thickness/Width of Fiber, Bond length, etc.)	S/D/T	AFRP BFRP CFRP GFRP	2D-DIC & SGs MFI & SGs LVDTs & DIC LVDTs & SGs Only SGs	Concrete fracture Debonding (FA/DC/CA) Fiber rupture	Bond strength models (Debonding strain) Bond–slip models Effective bond length Fracture energy	[24,27,76,77,78,79,80,81,82,83]
	Effects of Surface treatments	S/P	CFRP	Only LVDTs LVDTs & SGs	Debonding (FA/CA)	Summary of theories Bond strength models	[51,52,53]
	Effects of Concrete strength	S/D/P	AFRP BFRP CFRP GFRP	2D-DIC Only ESPI SGs & πGs LVDTs and/or SGs S-type load cell	Concrete splitting Debonding (FA/CA) Fiber rupture	Bond strength models Bond–slip models Effective bond length Fracture energy	[51,76,79,84,85,86,87,88,89,90]
	Debonding Mechanism	S/D/T	CFRP GFRP	PIV/3D-DIC Only MFI/EPSI LVDTs & DIC LVDTs & SGs Only SGs	Concrete failure Strips debonding (FA/DC/CA) Strips rupture	Bond strength models Bond–slip models Effective bond length Interfacial fracture energy	[56,84,85,91,92,93,94,95,96,97,98,99,100,101]
	Comparison between EBROG and EBR	S/D	CFRP GFRP	2D/PIV/3D-DIC S-type load cell SGs & πGs	Concrete failure (Wedge-shaped) FA or DC Strips rupture	Validated theories Effective bond length Fracture energy Bond strength models	[55,89,90,102,103,104,105,106,107]
	Environmental exposure conditions	S/D/T	CFRP GFRP	2D/PIV-DIC & SGs 3D-DIC & SGs LVDTs & SGs LVDTs Only SGs	Concrete failure (Splitting) Strips debonding (FA/CA/DC) Strips rupture	Validated theories (Gibson et al. model) Bond–slip models Bond strength models Fracture energy	[22,23,108,109,110,111,112,113,114,115,116,117,118,119,120]
	Dynamic behavior	S/D	BFRP CFRP	2D/PIV-DIC Only SGs LVDTs & SGs	Concrete failure DC or CA Strips rupture	Bond–slip (Dynamic) Bond strength models Effective bond length	[86,87,88,121,122,123,124,125,126]
	Fatigue behaviors	S/D	CFRP	LVDTs & DIC LVDTs & SGs	FRP strips debonding (FA/CA/DC)	Bond–slip (Fatigue) Bond strength models	[127,128,129,130]
	Mixed-mode (Failure/loading)	S/D	AFRP CFRP	2D-DIC only LVDT & SGs	Concrete peeling FA/CA/DC	Bond–slip (EBROG) Bond strength models	[131,132,133,134]
	Reviews	Not specific					[18,50,135,136]

**Notes:** 1. **S** means **S**ingle lap-shear tests, **D** means **D**ouble lap-shear test, **T** refers to direct **T**ension test, and **P** refers to **P**ullout tests. 2. **A/B/C/GFRP** refers to **A**ramid/**B**asalt/**C**arbon/**G**lass **F**iber-**R**einforced **P**olymers. 3. **SG**s refers to **S**train **G**auges, similar **πG**s means **π**-type **G**auges, **2D/3D/PIV-DIC** means 2/3 Dimensional/**P**article **I**mage **V**elocimetry-**D**igital **I**mage **C**orrelation, **LVDT** refers to **L**inear **V**ariable **D**isplacement **T**ransformer, **ESPI** means **E**lectronic **S**peckle **P**attern **I**nterferometry, and **MFI** refers to **M**oire **F**ringe **I**nterferometry. 4. **FA**, **CA,** and **DC** are three types of debonding failure modes; they are **F**iber-**A**dhesive layer/**C**oncrete-**A**dhesive layer/**D**ebonding in **C**oncrete substrate, which happen in different locations of the interfacial bond cohesive zone.

**Table 13:** Test-method decision matrix.

Performance Question	Most Informative Test Type	Main Advantage	Main Limitations for Field Transfer
Basic FRP–concrete bond capacity	Single-lap or double-lap shear.	Efficient isolation of interface behavior and debonding initiation.	Boundary restraint and eccentricity can differ from structural members.
Axial load transfer in tension strengthening	Direct tension test.	Closer to axial force-transfer demand.	Less common and harder to standardize across laboratories.
Anchor dowel capacity	Pullout/direct anchor test.	Separates embedment, dowel diameter, and concrete failure variables.	Does not capture fan–strip interaction or multi-anchor load sharing.
Member-level bending influence	Flexural or notched-beam setup.	Represents bending-induced interface demand.	Concrete-prism deformation and cracking complicate direct bond comparison.
System behavior with multiple anchors	COI-C multi-anchor EB-FRP system test.	Captures anchor spacing, redistribution, and system capacity.	Sparse evidence base; only 96 test results in the reviewed corpus.

**Table 14:** Durability and exposure synthesis.

Exposure Factor	Likely Affected Mechanism	Outcome Metrics to Report	Current Comparability Limit
Elevated temperature	Adhesive stiffness, glass-transition proximity, and interface fracture behavior.	Residual capacity, strain profile, failure mode, and temperature history.	Different temperature levels, durations, and heating protocols.
Moisture/wet–dry/hygrothermal aging	Adhesive-interface degradation and concrete surface-zone weakening.	Residual capacity, debonding strain, and bond–slip response.	Different moisture histories and aging durations.
Freeze–thaw exposure	Near-surface concrete damage and interface deterioration.	Residual capacity, failure-mode shift, and crack/interface observations.	Limited matched control specimens and inconsistent cycle protocols.
Long-term outdoor exposure	Combined thermal, moisture, UV, and aging effects.	Residual capacity and service-age context.	Site-specific exposure histories prevent pooled degradation factors.

**Table 16:** Comprehensive categorization of reviewed papers.

Level of COI	Objective	Test Type ^1^	FRP Type ^2^	Data Acquisition Strategies Used ^3^	Failure Modes Observed ^4^	Theory Development	Included Papers
**COI-B** Single-anchored and isolated anchor	Alignments	S	CFRP	LVDTs & SGs	Plate debonding Anchor rupture	Predictive model (load-slip) Bond model (fan to sheet)	[173,174,175,176]
	Configurations	S/T/P		Only DIC/ LVDTs & SGs	Concrete failure FRP strip fracture Anchor rupture	Anchor design Predictive model (rupture)	[30,37,38,39,177,178]
	Strength models	S		Built-in Load cell DIC & Load cell	Concrete cone Fiber rupture Dowel pullout	Predictive model (rupture) Straight/bent anchor design	[35,179,180,181,182,183]
	Typologies	S/D/P	CFRP GFRP	Only SGs LVDTs & SGs	Plate debonding Plate rupture Anchor rupture Anchor pullout	Bond–slip model Strength model	[36,184,185,186,187,188,189,190,191]
	Reviews	Not specific					[67]
**COI-C** Multi-anchored	Alignments	S	CFRP	Only LVDTs LVDTs & SG	Concrete failure (Cover separation) Plate debonding Anchor rupture Anchor pullout	Validated models (ACI) Anchored EB-FRP design	[30,40,45,46]
	Configurations	S/T		3D-DIC & SGs LVDTs & SGs	Concrete peeling Plate debonding Anchor rupture Anchor pullout	Anchor design Predictive model (load-slip)	[180,192,193]
	Typologies	S		Only SG LVDTs & SGs	Plate debonding Plate rupture Anchor rupture Anchor pullout	Predictive model (strength) Anchored EB-FRP design	[32,170,194,195,196]
	Reviews/codes	Not specific					[50,197,198]

**Table 17:** Anchor-configuration comparison.

Configuration/Test Focus	What It Can Support	Practical Advantage	Key Limitation
Straight isolated anchor pullout	Embedment depth, dowel diameter, and concrete-related failure limits.	Clean isolation of dowel/concrete mechanisms.	Does not represent EB-FRP plate or sheet load sharing.
Bent fan-anchor lap shear	Fan–strip force transfer and debonding delay.	Closer to retrofit detailing than pure pullout.	Sensitive to fan geometry, insertion angle, and fabrication quality.
Single-anchor EB-FRP system	Local anchorage effectiveness relative to an unanchored control.	Useful for practical detailing of one anchor.	Capacity increase is reference-specimen-dependent.
Multi-anchor EB-FRP system	Anchor spacing, interaction, redistribution, and system capacity.	Most relevant to field-strengthening layouts.	Small evidence base; load sharing remains under-validated.

**Table 18:** Model-accuracy evidence matrix.

Model Family	Accuracy Evidence That Can Be Compared	Validity Domain	Remaining Gap
FRP rupture models	Material strength and anchor cross-section can be checked where reported.	More stable when rupture governs and tensile properties are well defined.	Less reliable when mixed pullout/concrete failures occur.
Pullout/bond–shear models	Reported ultimate capacity and embedment variables.	Useful for isolated anchors with comparable embedment and dowel geometry.	Sensitive to boundary condition, shallow embedment, and interface assumptions.
Concrete cone/pryout models	Concrete strength, cone geometry, embedment depth, failure mode.	Applicable when concrete-related failure is observed.	Cone development and substrate condition vary across tests.
Anchored EB-FRP system models	System capacity where control specimens and anchor layouts are reported.	Most relevant to design when strip debonding and anchor contribution are both included.	Few multi-anchor validations and inconsistent error metrics.

**Table 19:** Existing isolated anchor strength models from 1995 to 2026 (COI-B).

Reference	Failure Modes	Types of Anchors	Strength Models	Notes
Fuchs et al. (1995) [181] Eligehausen et al. (2006) [182]	Concrete fails	Straight	$N_{cc}=14.7h_{c}^{1.5}\sqrt{f_{c}^{\prime }}$	The cone angle is 35 degrees
ACI 349-85 (1997) [183]	Concrete fails	Straight	$N_{cc}=f_{ct}A_{proj}$ , $f_{ct}=\frac{\sqrt{f_{c}}}{3},A_{proj}=\pi \left(h_{c}^{2}+h_{c}\cdot d_{0}\right)$	The cone angle is 45 degrees
Cook et al. (1998) [188]	Pullout	Bent	$N_{po}=\tau_{r}\pi d_{0}h_{ef}$	4 ≤ *hef*/*d*0 ≤ 20
Özdemir (2005) [36]	Mixed (CF+PO)	Straight	$N_{cc}=0.33\sqrt{f_{c}^{\prime }}h_{ef}\left(d_{0}+h_{ef}\right)\pi \;\;\;\;\;\;\;\;\;\left(h_{ef}<50\;\mathrm{mm}\right)$ $N_{cb}=0.33\sqrt{f_{c}^{\prime }}50\left(d_{0}+50\right)\pi +\pi \tau_{a}d_{0}\left(h_{ef}-50\right)$ $\left(h_{ef}>50\;\mathrm{mm}\right)$	$\tau_{r}$ represents the average bond shear stress along the dowel
Ozbakkaloglu and Saatcioglu (2009) [38]	Concrete fails	Straight	$N_{cc}=\pi {\left(f_{ct}\right)}_{exp}\left(h_{c}^{2}+h_{c}\cdot d_{0}\right)$	Modified ACI 349 method
	Mixed (CF + PO)	Straight	$N_{cb}=f_{ct}h_{c}\left(d_{0}+h_{c}\right)\pi +\pi \tau d_{0}(h_{ef}-h_{c})$	$h_{ef}>h_{c}$
	Rupture	Straight	$N_{fr}=\alpha A_{f}f_{f}$ , 0.3≤α≤0.5	Reduction coefficient α on potential manufacturing errors
Kim and Smith (2009) [37]	Concrete fails	Straight	$N_{cc}=12.04h_{ef}^{1.5}\sqrt{f_{c}^{\prime }}$	$17.5\;\mathrm{mm}\leq h_{ef}\leq 100\;\mathrm{mm}$ $10.4\;\mathrm{MPa}\leq f_{c}^{\prime }\leq 100\;\mathrm{MPa}$ $11.8\;\mathrm{mm}\leq d_{0}\leq 20\;\mathrm{mm}$
	Rupture	Straight	$N_{fr}=0.59A_{f}f_{f}$	
	Mixed (CF + PO)	Straight	$N_{cb}=5.64\pi d_{0}h_{ef}\;\;\;\;\;\;(f_{c}^{\prime }<20\;\mathrm{MPa})$ $N_{cb}=10.86\pi d_{0}h_{ef}\;\;\;\;\;\;(f_{c}^{\prime }\geq 20\;\mathrm{MPa})$	
Mahrenholtz et al. (2015) [39]	Rupture	Bent	$N_{fr}=0.06A_{f}f_{f}$	${{76\;\mathrm{mm}}^{2}\leq A}_{f}\leq 201\;{\mathrm{mm}}^{2}$
Llauradó et al. (2017) [40]	Concrete fails	Straight	$N_{u}=\min(N_{cc},N_{cb},N_{u})$ $N_{cc}=9.68h_{emb}^{1.5}\sqrt{f_{c}^{\prime }}$ $N_{cb}=\tau \pi d_{0}h_{emb}$ $N_{u}=\gamma w_{FRP}t_{FRP}f_{FRP}$	τ=4.62 MPa $\left(f_{c}^{\prime }<20\;\mathrm{MPa}\right)$ τ=9.07 MPa $\left(f_{c}^{\prime }\geq 20\;\mathrm{MPa}\right)$ γ=0.59 used when rolled and bundled fibers anchors are tested
Kanitkar et al.(2016) [174]	Fan debond	Straight	$N_{sd}=0.35\tau_{r}A_{f}$	${{6500\;\mathrm{mm}}^{2}\leq A}_{f}\leq 16,800\;{\mathrm{mm}}^{2}$
Llauradó et al. (2018) [178]	Rupture	Bent	$N_{fr}=0.002A_{f}f_{f}\left(h_{ef}+\frac{50\beta }{\pi }r_{b}\right)$ $\left({50\;\mathrm{mm}\leq h}_{ef}\leq 125\;\mathrm{mm}\right)$	${{201\;\mathrm{mm}}^{2}\leq A}_{f}\leq 314\;{\mathrm{mm}}^{2}$ ${0.3\;\mathrm{mm}\leq r}_{b}\leq 2.5\;\mathrm{mm}$ 90°≤β≤135°
del Rey Castillo et al. (2018) [187]	Pullout	Bent	$N_{po}=700h_{ef}-18,000$	$40\;\mathrm{mm}\leq h_{ef}\leq 100\;\mathrm{mm}$
del Rey Castillo et al. (2019, 2026) [179,204]	Rupture	Straight	$N_{fr}=6.25E_{f}\varepsilon_{f}A_{dowel}^{0.56}\left(\frac{90-\alpha }{90}\right)$	15°≤α≤60° 90°≤β≤180° ${28\;{\mathrm{mm}}^{2}\leq A}_{dowel}\leq 84\;{\mathrm{mm}}^{2}$ $E_{f}=253GPa,\varepsilon_{f}=0.98\%$
		Bent	$N_{fr}=3.75E_{f}\varepsilon_{f}A_{dowel}^{0.62}(\frac{90-\alpha }{90}))$	
del Rey Castillo et al. (2019) [175]	Fan debond	Straight	$N_{sb}=Max(5A_{f},\;0.1\sigma_{r}A_{f})$	When $\tau_{r}$ resin bond shear strength not available
Singh et al. (2019) [176]	Fan debond	Bent (single lap shear)	$N_{sd}=0.4\sigma_{r}b_{f}L$ $\;\;(L<L_{cr})$ , $N_{sd}=0.6\sigma_{f}b_{f}t_{f}$ $\;\;(L\geq L_{cr})$	$L_{cr}=3\sigma_{f}t_{f}/2\sigma_{r}$
		Bent (double lap shear)	$N_{sd}=0.6\sigma_{r}b_{f}L$ $\;\;(L<L_{cr})$ , $N_{sd}=1.2\sigma_{f}b_{f}t_{f}$ $\;\;(L\geq L_{cr})$	$L_{cr}=2\sigma_{f}t_{f}/\sigma_{r}$
Boumakis et al. (2022) [189]	Pullout	Bent	$N_{po}=\tau (s,t)\pi d_{0}h_{ef}$ $\tau \left(s,t\right)=\;\tau (s)C(t,t^{\prime })$ where $C(t,t^{\prime })$ is Dirichlet series	τ(s,t) is time-dependent viscoelastic bond–slip relation
Guadagnuolo et al. (2023) [190]	Pullout	Straight	$N_{fr}=\left[1.0746\left(\tau^{*}-\tau \right)+0.0038\right]\pi d_{0}h_{ef}\;\;\;\;\left(fc^{\prime }\leq 28\;\mathrm{MPa}\right)$ $N_{fr}=[1.0674\left(\tau^{*}-\tau \right)+0.0059$ ] $\pi d_{0}h_{ef}\;\;\;\;\left(fc^{\prime }>28\;\mathrm{MPa}\right)$	8.22 $\leq d_{0}\leq$ 21.2132.88 $\leq h_{ef}\leq$ 135 $15\leq f_{c}^{\prime }\leq 58.2$
Zhang et al. (2026) [191]	Pullout	Bent/Straight	$N_{u}=\sqrt{f_{c}^{\prime }}h_{ef}\left[\left(2.35{d_{0}}^{2}+78.4\right)\times 10^{3}+55.6\sqrt{{C_{\beta }d}_{0}}\right]$	$C_{\beta }=sin\beta$ for bent $C_{\beta }=0.085$ for straight

**Table 20:** Existing anchored EB-FRP capacity predictive models from 2013 to 2026 (COI-C).

Models	Capacity of Anchored EB-FRP
Zhang and Smith (2013) [30]	$P_{u}=\alpha \kappa_{m}P_{d},$ where $\kappa_{m}$ is an enhancement factor
Llauradó et al. (2017) [40]	$P_{u}=min(P_{d}+0.58nP_{a},\;1.58P_{d}),$ where n is the number of anchors
Cortez Flores et al. (2020) [180]	$P_{u}=P_{d}+k_{g}P_{a},$ where $k_{g}$ is a reduction factor
CNR DT200 R2/2024 [198]	$P_{u}=min\left(P_{d}+min\left[n\left\{P_{po},\;P_{cc},P_{sd},P_{fr}\right\}\right],\;\frac{f_{f}}{\eta_{a}\gamma_{f1}}b_{f}t_{f}\right),$ where $f_{f}$ , $b_{f}$ and $t_{f}$ are tensile strength, width and thickness of FRP strips
Zhang et al. (2026) [196]	$P_{u}=\alpha P_{d}+\beta \sum P_{a},$ where α=0.93 when $l_{b0}\leq l_{e}$ , or α=0 , and $\beta =1.5\varphi_{a}$ when $l_{a}\leq l_{e}+l_{f}$ , or β=1.77/N.

**Table 21:** Evidence-based future research priorities.

Priority	Evidence Gap	Recommended Study Type	Expected Design Impact
High	Normalized benchmark data across specimen scales, FRP types, and test methods.	Shared experimental database with common geometry, material, and failure-mode fields.	Comparable model validation and less ambiguous design calibration.
High	Multi-anchor interaction and anchor–strip load sharing.	COI-C tests with controlled anchor spacing, fan geometry, DIC/fiber optic strain fields, and repeated specimens.	Design rules for anchor spacing, reduction factors, and system capacity.
High	Durability under temperature, wet–dry, freeze–thaw, fatigue, and long-term exposure.	Matched control/exposure tests with residual capacity, strain, and failure-mode reporting.	Environmental reduction factors and service-life guidance.
Medium	Field-scale transfer from small coupons to members.	Large-scale member tests with realistic substrate conditions and construction variability.	More reliable limits for applying coupon-derived bond models.
Medium	Numerical modeling linked to experiments.	Validated FEM/analytical studies calibrated against full-field test data.	Mechanics-based models that remain practical for design.

## Data Availability

The data presented in this study are openly available in Experimental Database of FRP Bonded Strips/Ties Tests for Externally Bonded FRP Strengthening on Reinforced Concrete Structures (1994–2026) at https://doi.org/10.17608/k6.auckland.24190647.

## Abbreviations

$A_{d}$	Cross-sectional area of anchor dowel (mm^2^)
$A_{f}$	Cross-sectional area of FRP strip (mm^2^)
$b_{c}$	Width of concrete member (mm)
$b_{f}$	Width of FRP strip (mm)
$d_{0}$	Diameter of anchor dowel (mm)
$E_{a}$	Elastic modulus of FRP anchor (MPa)
$E_{f}$	Elastic modulus of FRP strip (MPa)
$f_{c}^{\prime }$	Concrete compressive strength (MPa)
$f_{t}$	Concrete tensile strength (MPa)
$G_{a}$	Adhesive shear modulus (GPa)
$G_{f}$	Fracture energy (GPa)
$k_{f}$	Stiffness ratio of FRP–concrete interface (mm^−1^)
$l_{a}$	Spacing between anchors (mm)
$l_{e}$	Effective bond length (mm)
$l_{b}$	FRP bond length (mm)
$l_{f}$	FRP anchor fan length (mm)
$h_{ef}$	Anchor embedment depth (mm)
s	Interfacial slip (mm)
$s_{f}$	Ultimate slip at failure (mm)
$\tau_{max}$	Peak bond stress (MPa)
$\varepsilon_{d}$	Debonding strain
$P_{d}$	Debonding force (kN)
$P_{u}$	Peak force (kN)
$t_{a}$	Thickness of adhesive layer (mm)
$t_{f}$	Thickness of one-layer FRP strip (mm)
$T_{f}$	Total thickness of FRP strips (mm)
α	Half angle of anchor fan (degrees)
β	Insertion angle of anchor dowel (degrees)
$\beta_{w}$	Width ratio of FRP and concrete substrate
n	Number of FRP strip layers
N	Number of fiber anchors

## References

[1] Teng J., Chen J.-F., Yu T. (2002). FRP-Strengthened RC Structures.

[2] Bank L.C. (2006). Composites for Construction: Structural Design with FRP Materials.

[3] Attari N., Amziane S., Chemrouk M. (2012). Flexural strengthening of concrete beams using CFRP, GFRP and hybrid FRP sheets. Constr. Build. Mater..

[4] Sun W., Jirsa J.O., Ghannoum W.M. (2016). Behavior of Anchored Carbon Fiber-Reinforced Polymer Strips Used for Strengthening Concrete Structures. ACI Mater. J..

[5] Täljsten B. (1997). Strengthening of Beams by Plate Bonding. J. Mater. Civ. Eng..

[6] del Rey Castillo E., Ingham J., Griffith M. (2017). Seismic strengthening of RC columns with straight FRP anchors. Proceedings of the 13th conference on Fiber Reinforced Polymers in Reinforced Concrete Structures (FRPRCS-13).

[7] del Rey Castillo E., Griffith M., Ingham J. (2018). Seismic behavior of RC columns flexurally strengthened with FRP sheets and FRP anchors. Compos. Struct..

[8] del Rey Castillo E., Kanitkar R. (2020). Effect of FRP Spike Anchor Installation Quality and Concrete Repair on the Seismic Behavior of FRP-Strengthened RC Columns. J. Compos. Constr..

[9] Ormeno M., Jing J., Rogers R., Del Rey Castillo E. (2019). Capacity of diaphragm strengthened with FRP: Comparison between ACI 440.2 R and in-situ tests. Proceedings of the 2019 New Zealand Society for Earthquake Engineering and Pacific Conference on Earthquake Engineering.

[10] Hosseini A., Wesson M., Borwankar A., Sumer A., Love J., Moore E. Experimental Investigation Into Strengthening of Concrete Diaphragm Collectors Using Unanchored and Anchored Multilayer FRP Reinforcement. Proceedings of the Structural Engineers Association of California (SEAOC).

[11] Zhang J., del Rey Castillo E., Kanitkar R., D Borwankar A. Behaviour of large FRP ties for seismic strengthening of concrete diaphragms. Proceedings of the Structural Engineering Society New Zealand (SESOC) 2023 Conference.

[12] Mostofinejad D., Anaei M.M. (2012). Effect of confining of boundary elements of slender RC shear wall by FRP composites and stirrups. Eng. Struct..

[13] Woods J., Cruz-Noguez C., Lau D. An Innovative FRP Anchor System for the Seismic Retrofit of Reinforced Concrete Shear Walls. Proceedings of the 10th National Conference in Earthquake Engineering.

[14] Mosallam A.S., Nasr A. (2017). Structural performance of RC shear walls with post-construction openings strengthened with FRP composite laminates. Compos. Part B Eng..

[15] Pampanin S., Bolognini D., Pavese A. (2007). Performance-based seismic retrofit strategy for existing reinforced concrete frame systems using FRP composites. J. Compos. Constr..

[16] Castillo E.d.R., Niroomandi A., Triantafillou T. (2024). Seismic Retrofitting of Realistic Beam–Column Joints with Shear Failure Using FRP Sheets and FRP Anchors. J. Compos. Constr..

[17] Kamiharako A., Shimomura T., Maruyama K., Nishida H. (1999). Analysis of bond and debonding behavior of continuous fiber sheet bonded on concrete. Doboku Gakkai Ronbunshu.

[18] Smith S.T., Teng J. (2002). FRP-strengthened RC beams. I: Review of debonding strength models. Eng. Struct..

[19] Teng J., Smith S.T., Yao J., Chen J.-F. (2003). Intermediate crack-induced debonding in RC beams and slabs. Constr. Build. Mater..

[20] Yao J. (2004). Debonding Failures in RC Beams and Slabs Strengthened with FRP Plates.

[21] Zhang J., del Rey Castillo E., Kanitkar R., Borwankar A.D., Ramprasath R. (2024). Proposed Design Method for EB-FRP Ties Debond Strain Encompassing Short/Long and Thin/Thick Ties. ACI Symp. Publ..

[22] Leone M., Matthys S., Aiello M.A. (2009). Effect of elevated service temperature on bond between FRP EBR systems and concrete. Compos. Part B Eng..

[23] Ferrier E., Rabinovitch O., Michel L. (2016). Mechanical behavior of concrete–resin/adhesive–FRP structural assemblies under low and high temperatures. Constr. Build. Mater..

[24] Yang G., Zomorodian M., Belarbi A., Ayoub A. (2016). Uniaxial tensile stress-strain relationships of RC elements strengthened with FRP sheets. J. Compos. Constr..

[25] Rahimi H., Hutchinson A. (2001). Concrete beams strengthened with externally bonded FRP plates. J. Compos. Constr..

[26] Ahmed A.A., Hassan M., Masmoudi R. (2020). Effect of concrete strength and tube thickness on the flexural behavior of prestressed rectangular concrete-filled FRP tubes beams. Eng. Struct..

[27] Hallonet A., Michel L., Ferrier E. (2016). Investigation of the bond behavior of flax FRP strengthened RC structures through double lap shear testing. Compos. Part B Eng..

[28] Zhang J., Del Rey Castillo E. (2023). Experimental Database of 3162 Unanchored FRP Bonded Strips/Ties Tests for Externally Bonded FRP Strengthening on Reinforced Concrete Structures (1994–2024).

[29] Smith S.T., Hu S., Kim S.J., Seracino R. (2011). FRP-strengthened RC slabs anchored with FRP anchors. Eng. Struct..

[30] Zhang H., Smith S.T. (2013). Fibre-reinforced polymer (FRP)-to-concrete joints anchored with FRP anchors: Tests and experimental trends. Can. J. Civ. Eng..

[31] Zhang J., del Rey Castillo E., Kanitkar R., Barris C., Borwankar A. Tension Strengthening of Reinforced Concrete using Large-Scale EB-FRP with and without Fiber Anchors. Proceedings of the 17th International Symposium on Fiber-Reinforced Polymer (FRP) Reinforcement for Concrete Structures.

[32] Zhang H.W., Smith S.T. (2012). Influence of FRP anchor fan configuration and dowel angle on anchoring FRP plates. Compos. Part B Eng..

[33] Jiang X., Tan C., Qiang X., Han S., Shen A. (2023). An innovative circular anchor system for CFRP plates: Experimental investigation and field application. Case Stud. Constr. Mater..

[34] del Rey Castillo E., Kanitkar R., Smith S. (2019). Practical uses of FRP anchors in New Zealand. Proceedings of the 2019 Conference of the Structural Engineering Society.

[35] Zhang H.W., Smith S.T., Kim S.J. (2012). Optimisation of carbon and glass FRP anchor design. Constr. Build. Mater..

[36] Özdemir G. (2005). Mechanical Properties of CFRP Anchorages. Master’s Thesis.

[37] Kim S., Smith S.T. (2009). Behaviour of handmade FRP anchors under tensile load in uncracked concrete. Adv. Struct. Eng..

[38] Ozbakkaloglu T., Saatcioglu M. (2009). Tensile behavior of FRP anchors in concrete. J. Compos. Constr..

[39] Mahrenholtz P., Park J.-M., Cho J.-Y. (2015). Monotonic and cyclic behaviour of isolated FRP anchors loaded in shear. Compos. Part B Eng..

[40] Llauradó P.V., Ibell T., Gómez J.F., Ramos F.J.G. (2017). Pull-out and shear-strength models for FRP spike anchors. Compos. Part B Eng..

[41] Kanitkar R., del Rey Castillo E., Smith S., Rasheed H. Quality control and assurance for FRP anchors—Current practice and path forward. Proceedings of the Seventh Asia-Pacific Conference on FRP in Structures (APFIS 2019).

[42] Zhang J., del Rey Castillo E. (2025). Experimental Database of 310 Fiber Anchor Tests for Externally Bonded FRP Strengthening on Reinforced Concrete Structures (2005–2025).

[43] Zhang J., del Rey Castillo E., Kanitkar R., Lin S., Allen T., Hogan L., Borwankar A. Failure Modes of Bent FRP Anchor with Shallow Embedment. Proceedings of the 2024 fib Symposium.

[44] del Rey Castillo E., Dizhur D., Griffith M., Ingham J. (2019). Experimental testing and design model for bent FRP anchors exhibiting fiber rupture failure mode. Compos. Struct..

[45] Zhang H., Smith S.T. (2012). FRP-to-concrete joint assemblages anchored with multiple FRP anchors. Compos. Struct..

[46] Flores I.A.C., Gómez J.F., Llauradó P.V. (2019). Influence of multiple anchor arrangement in the behaviour of FRP-to-concrete anchored joints. Compos. Struct..

[47] (2017). Guide for the Design and Construction of Externally Bonded FRP Systems for Strengthening Concrete Structures.

[48] Consiglio Nazionale delle Ricerche (CNR) (2013). Guide for the Design and Construction of Externally Bonded FRP Systems for Strengthening Existing Structures.

[49] Matthys S., EAR fib Working Group (2019). Externally Applied FRP Reinforcement for Concrete Structures.

[50] del Rey Castillo E., Harries K.A., Rogers R., Kanitkar R. (2022). FRP Tension Ties: State-of-the-Art Review of Existing Design Guidance for Debonding Capacity and Applicability to Concrete Diaphragm Seismic Strengthening. J. Compos. Constr..

[51] Ohu R., Jaafar M., Aznieta F., Alwathaf A. (2013). Effect of surface treatment on bond of embedded carbon fiber-reinforced polymer plates. J. Compos. Mater..

[52] Biscaia H.C., Chastre C., Borba I.S., Silva C., Cruz D. (2016). Experimental evaluation of bonding between CFRP laminates and different structural materials. J. Compos. Constr..

[53] Soares S., Sena-Cruz J., Cruz J.R., Fernandes P. (2019). Influence of surface preparation method on the bond behavior of externally bonded CFRP reinforcements in concrete. Materials.

[54] del Rey Castillo E., Allen T., Henry R., Griffith M., Ingham J. (2019). Digital image correlation (DIC) for measurement of strains and displacements in coarse, low volume-fraction FRP composites used in civil infrastructure. Compos. Struct..

[55] Hosseini A., Mostofinejad D. (2013). Experimental investigation into bond behavior of CFRP sheets attached to concrete using EBR and EBROG techniques. Compos. Part B Eng..

[56] Hosseini A., Mostofinejad D. (2014). Effective bond length of FRP-to-concrete adhesively-bonded joints: Experimental evaluation of existing models. Int. J. Adhes. Adhes..

[57] Dai J., Ueda T., Sato Y. (2005). Development of the nonlinear bond stress–slip model of fiber reinforced plastics sheet–concrete interfaces with a simple method. J. Compos. Constr..

[58] Mensah C., Bonsu A.O., Wang Z. (2022). Interfacial behavior of externally bonded BFRP-to-concrete joints using different epoxy adhesives. Int. J. Adhes. Adhes..

[59] Michels J., Staśkiewicz M., Czaderski C., Lasek K., Kotynia R., Motavalli M. (2014). Anchorage resistance of CFRP strips externally bonded to various cementitious substrates. Compos. Part B Eng..

[60] Shi J.-W., Cao W.-H., Wu Z.-S. (2019). Effect of adhesive properties on the bond behaviour of externally bonded FRP-to-concrete joints. Compos. Part B Eng..

[61] Liberati A., Altman D.G., Tetzlaff J., Mulrow C., Gøtzsche P.C., Ioannidis J.P., Clarke M., Devereaux P.J., Kleijnen J., Moher D. (2009). The PRISMA statement for reporting systematic reviews and meta-analyses of studies that evaluate health care interventions: Explanation and elaboration. J. Clin. Epidemiol..

[62] Moher D., Altman D.G., Liberati A., Tetzlaff J. (2011). PRISMA statement. Epidemiology.

[63] Page M.J., McKenzie J.E., Bossuyt P.M., Boutron I., Hoffmann T.C., Mulrow C.D., Shamseer L., Tetzlaff J.M., Akl E.A., Brennan S.E. (2021). The PRISMA 2020 statement: An updated guideline for reporting systematic reviews. BMJ.

[64] Khan K.S., Kunz R., Kleijnen J., Antes G. (2003). Five steps to conducting a systematic review. J. R. Soc. Med..

[65] Ahmad S., Sohail M., Waris A., Elginaid A., Mohammed I. (2018). SCImago, eigenfactor score, and H5 index journal rank indicator: A study of journals in the area of construction and building technologies. DESIDOC J. Libr. Inf. Technol..

[66] Singh V.K., Singh P., Karmakar M., Leta J., Mayr P. (2021). The journal coverage of Web of Science, Scopus and Dimensions: A comparative analysis. Scientometrics.

[67] Muciaccia G., Khorasani M., Mostofinejad D. (2022). Effect of different parameters on the performance of FRP anchors in combination with EBR-FRP strengthening systems: A review. Constr. Build. Mater..

[68] Sanginabadi K., Yazdani A., Mostofinejad D., Czaderski C. (2022). RC members externally strengthened with FRP composites by grooving methods including EBROG and EBRIG: A state-of-the-art review. Constr. Build. Mater..

[69] Zhang J., Kanitkar R., del Rey Castillo E., Harries K.A., Rogers R., Borwankar A. Bond behaviour of FRP for pure axial tension strengthening of concrete. Proceedings of the 11th International Conference on Fiber-Reinforced Polymer (FRP) Composites in Civil Engineering (CICE 2023).

[70] Zhang J., del Rey Castillo E., Allen T., Hogan L., Kanitkar R., Barris C., Carloni C., Borwankar A. (2025). Semi-empirical model for load-slip response of externally bonded FRP strengthened reinforced concrete structures. Case Stud. Constr. Mater..

[71] Mostofinejad D., Sanginabadi K., Eftekhar M.R. (2019). Effects of coarse aggregate volume on CFRP-concrete bond strength and behavior. Constr. Build. Mater..

[72] Sanginabadi K., Mostofinejad D. (2021). Effect of aggregate content on the CFRP-concrete effective bond length: An experimental and analytical study. Compos. Struct..

[73] Yuan C., Chen W., Pham T.M., Hao H. (2018). Bond behavior between basalt fibres reinforced polymer sheets and steel fibres reinforced concrete. Eng. Struct..

[74] Fazli H., Yan D., Zhang Y., He Y., Hu Y. (2022). Influence of coarse aggregate size on the bonding between CFRP Sheets and metakaolin-based geopolymer concrete and ordinary concrete. J. Compos. Constr..

[75] Yuan C., Chen W., Pham T.M., Hao H. (2019). Effect of aggregate size on bond behaviour between basalt fibre reinforced polymer sheets and concrete. Compos. Part B Eng..

[76] Nakaba K., Kanakubo T., Furuta T., Yoshizawa H. (2001). Bond behavior between fiber-reinforced polymer laminates and concrete. Struct. J..

[77] Subramaniam K.V., Carloni C., Nobile L. (2007). Width effect in the interface fracture during shear debonding of FRP sheets from concrete. Eng. Fract. Mech..

[78] Sharma S., Ali M.M., Goldar D., Sikdar P. (2006). Plate–concrete interfacial bond strength of FRP and metallic plated concrete specimens. Compos. Part B Eng..

[79] Biscaia H.C., Silva M.A., Chastre C. (2015). Factors influencing the performance of externally bonded reinforcement systems of GFRP-to-concrete interfaces. Mater. Struct..

[80] Zhu H., Wu G., Shi J., Liu C., He X. (2014). Digital image correlation measurement of the bond–slip relationship between fiber-reinforced polymer sheets and concrete substrate. J. Reinf. Plast. Compos..

[81] Yuan C., Chen W., Pham T.M., Hao H. (2019). Bond behaviour between hybrid fiber reinforced polymer sheets and concrete. Constr. Build. Mater..

[82] Tripi J.M., Bakis C.E., Boothby T.E., Nanni A. (2000). Deformation in concrete with external CFRP sheet reinforcement. J. Compos. Constr..

[83] Woo S.-K., Lee Y. (2010). Experimental study on interfacial behavior of CFRP-bonded concrete. KSCE J. Civ. Eng..

[84] Chajes M.J., Finch W.W., Thomson T.A. (1996). Bond and force transfer of composite-material plates bonded to concrete. Struct. J..

[85] Cao S., Chen J.F., Pan J., Sun N. (2007). ESPI measurement of bond-slip relationships of FRP-concrete interface. J. Compos. Constr..

[86] Yuan C., Chen W., Pham T.M., Hao H., Cui J., Shi Y. (2020). Influence of concrete strength on dynamic interfacial fracture behaviour between fibre reinforced polymer sheets and concrete. Eng. Fract. Mech..

[87] Shen D., Shi H., Ji Y., Yin F. (2015). Strain rate effect on effective bond length of basalt FRP sheet bonded to concrete. Constr. Build. Mater..

[88] Zhang Z., Xiao Y., Zhuge P., Zhang X. (2019). Experimental Investigation on the Interfacial Debonding between FRP Sheet and Concrete under Medium Strain Rate. Int. J. Polym. Sci..

[89] Moghaddas A., Mostofinejad D. (2019). Empirical FRP-concrete bond strength model for externally bonded reinforcement on grooves. J. Compos. Constr..

[90] Zhang P., Hu Y., Pang Y., Feng H., Gao D., Zhao J., Sheikh S.A. (2020). Influence factors analysis of the interfacial bond behavior between GFRP plates, concrete. Structures.

[91] Yao J., Teng J., Chen J.F. (2005). Experimental study on FRP-to-concrete bonded joints. Compos. Part B Eng..

[92] Czaderski C., Soudki K., Motavalli M. (2010). Front and side view image correlation measurements on FRP to concrete pull-off bond tests. J. Compos. Constr..

[93] Ali-Ahmad M., Subramaniam K., Ghosn M. (2006). Experimental investigation and fracture analysis of debonding between concrete and FRP sheets. J. Eng. Mech..

[94] Ceroni F., Pecce M., Matthys S. (2004). Tension stiffening of reinforced concrete ties strengthened with externally bonded fiber-reinforced polymer sheets. J. Compos. Constr..

[95] Carloni C., Subramaniam K.V. (2010). Direct determination of cohesive stress transfer during debonding of FRP from concrete. Compos. Struct..

[96] Wu Z., Yuan H., Yoshizawa H., Kanakubo T. (2001). Experimental/analytical study on interfacial fracture energy and fracture propagation along FRP-concrete i nterface. Spec. Publ..

[97] Lu X., Ye L., Teng J., Jiang J. (2005). Meso-scale finite element model for FRP sheets/plates bonded to concrete. Eng. Struct..

[98] Bilotta A., Di Ludovico M., Nigro E. (2011). FRP-to-concrete interface debonding: Experimental calibration of a capacity model. Compos. Part B Eng..

[99] Lu X., Teng J., Ye L., Jiang J. (2005). Bond–slip models for FRP sheets/plates bonded to concrete. Eng. Struct..

[100] Pham H.B., Al-Mahaidi R. (2007). Modelling of CFRP-concrete shear-lap tests. Constr. Build. Mater..

[101] Wu Y.-F., Jiang C. (2013). Quantification of bond-slip relationship for externally bonded FRP-to-concrete joints. J. Compos. Constr..

[102] Moshiri N., Czaderski C., Mostofinejad D., Motavalli M. (2021). Bond resistance of prestressed CFRP strips attached to concrete by using EBR and EBROG strengthening methods. Constr. Build. Mater..

[103] Mofrad M.H., Mostofinejad D., Hosseini A. (2019). A generic non-linear bond-slip model for CFRP composites bonded to concrete substrate using EBR and EBROG techniques. Compos. Struct..

[104] Ghahsareh F.M., Mostofinejad D. (2022). Effects of groove angle and pattern on cfrp-to-concrete bond behavior of ebrog joints: Comparison of diagonal with longitudinal and transverse grooves. Constr. Build. Mater..

[105] Moghaddas A., Mostofinejad D., Ilia E. (2019). Empirical FRP-concrete effective bond length model for externally bonded reinforcement on the grooves. Compos. Part B Eng..

[106] Tajmir-Riahi A., Moshiri N., Mostofinejad D. (2019). Bond mechanism of EBROG method using a single groove to attach CFRP sheets on concrete. Constr. Build. Mater..

[107] Mostofinejad D., Mofrad M.H., Hosseini A., Mofrad H.H. (2018). Investigating the effects of concrete compressive strength, CFRP thickness and groove depth on CFRP-concrete bond strength of EBROG joints. Constr. Build. Mater..

[108] Quiertant M., Benzarti K., Schneider J., Landrin F., Landrin M., Boinski F. (2017). Effects of ageing on the bond properties of carbon fiber reinforced polymer/concrete adhesive joints: Investigation using a modified double shear test. J. Test. Eval..

[109] Tajmir-Riahi A., Moshiri N., Mostofinejad D. (2019). Inquiry into bond behavior of CFRP sheets to concrete exposed to elevated temperatures–Experimental & analytical evaluation. Compos. Part B Eng..

[110] Firmo J., Correia J., Pitta D., Tiago C., Arruda M. (2015). Experimental characterization of the bond between externally bonded reinforcement (EBR) CFRP strips and concrete at elevated temperatures. Cem. Concr. Compos..

[111] Pan Y., Xian G. (2019). Influence of long-term outdoor exposure in a frigid zone on the CFRP-to-concrete bond behavior. Constr. Build. Mater..

[112] Al-Jaberi Z., Myers J.J., Chandrashekhara K. (2019). Effect of direct service temperature exposure on the bond behavior between advanced composites and CMU using NSM and EB techniques. Compos. Struct..

[113] Subramaniam K.V., Ali-Ahmad M., Ghosn M. (2008). Freeze–thaw degradation of FRP–concrete interface: Impact on cohesive fracture response. Eng. Fract. Mech..

[114] Al-Lami K., Colombi P., D’Antino T. (2020). Influence of hygrothermal ageing on the mechanical properties of CFRP-concrete joints and of their components. Compos. Struct..

[115] Lu Y., Zhu T., Li S., Liu Z. (2018). Bond behavior of wet-bonded carbon fiber-reinforced polymer-concrete interface subjected to moisture. Int. J. Polym. Sci..

[116] Al-Tamimi A.K., Hawileh R.A., Abdalla J.A., Rasheed H.A., Al-Mahaidi R. (2015). Durability of the bond between CFRP plates and concrete exposed to harsh environments. J. Mater. Civ. Eng..

[117] Gravina R., Hadigheh S.A., Setunge S. (2014). Interfacial bond strength of resin-impregnated fibre-reinforced polymer laminates bonded to concrete using vacuum and heat: Experimental study. Aust. J. Struct. Eng..

[118] Foster S., Bisby L. (2008). Fire survivability of externally bonded FRP strengthening systems. J. Compos. Constr..

[119] Carlos T.B., Rodrigues J.P.C. (2018). Experimental bond behaviour of a CFRP strengthening system for concrete elements at elevated temperatures. Constr. Build. Mater..

[120] Harmanci Y.E., Michels J., Chatzi E. (2018). Behaviour of prestressed CFRP anchorages during and after freeze-thaw cycle exposure. Polymers.

[121] Shen D., Ji Y., Yin F., Zhang J. (2015). Dynamic bond stress-slip relationship between basalt FRP sheet and concrete under initial static loading. J. Compos. Constr..

[122] Shen D., Shi X., Ji Y., Yin F. (2015). Strain rate effect on bond stress–slip relationship between basalt fiber-reinforced polymer sheet and concrete. J. Reinf. Plast. Compos..

[123] Yuan C., Chen W., Pham T.M., Hao H., Cui J., Shi Y. (2020). Dynamic interfacial bond behaviour between basalt fiber reinforced polymer sheets and concrete. Int. J. Solids Struct..

[124] Yuan C., Chen W., Pham T.M., Hao H., Cui J., Shi Y. (2019). Strain rate effect on interfacial bond behaviour between BFRP sheets and steel fibre reinforced concrete. Compos. Part B Eng..

[125] Salimian M.S., Mostofinejad D. (2019). Experimental evaluation of CFRP-concrete bond behavior under high loading rates using particle image velocimetry method. J. Compos. Constr..

[126] Yuan C., Chen W., Pham T.M., Hao H., Cui J., Shi Y. (2020). Interfacial bond behaviour between hybrid carbon/basalt fibre composites and concrete under dynamic loading. Int. J. Adhes. Adhes..

[127] Zhang W. (2016). Experimental study on fatigue behaviour of CFRP plates externally bonded to concrete substrate. Struct. Concr..

[128] Zhang W., Yan J.-B. (2016). Fatigue properties of shear–peeling debonding between CFRP plates and concrete. Mag. Concr. Res..

[129] Carloni C., Subramaniam K.V. (2013). Investigation of sub-critical fatigue crack growth in FRP/concrete cohesive interface using digital image analysis. Compos. Part B Eng..

[130] Carloni C., Subramaniam K.V., Savoia M., Mazzotti C. (2012). Experimental determination of FRP–concrete cohesive interface properties under fatigue loading. Compos. Struct..

[131] Zhang W. (2016). Experimental study on shear-peeling bond strength between a CFRP plate and concrete. Mag. Concr. Res..

[132] Alam M.S., Kanakubo T., Yasojima A. (2012). Shear-Peeling Bond Strength between Continuous Fiber Sheet and Concrete. ACI Struct. J..

[133] Ghorbani M., Mostofinejad D., Hosseini A. (2017). Bond behavior of CFRP sheets attached to concrete through EBR and EBROG joints subject to mixed-mode I/II loading. J. Compos. Constr..

[134] Ghorbani M., Mostofinejad D., Hosseini A. (2017). Experimental investigation into bond behavior of FRP-to-concrete under mixed-mode I/II loading. Constr. Build. Mater..

[135] Ghaleh R.Z., Mostofinejad D. (2022). Behaviour of EBRIG CFRP sheet-concrete joint: Comparative assessment with EBR and EBROG methods. Constr. Build. Mater..

[136] Alsuhaibani E. (2025). Nondestructive Testing of Externally Bonded FRP Concrete Structures: A Comprehensive Review. Polymers.

[137] Focacci F., Rahman M.M., D’Antino T., Carloni C. (2024). FRP Strips Externally Bonded to Quasi-Brittle Substrates: Discussions and Advances of Open Research Topics. J. Compos. Constr..

[138] Salomoni V., Mazzucco G., Pellegrino C., Majorana C. (2011). Three-dimensional modelling of bond behaviour between concrete and FRP reinforcement. Eng. Comput..

[139] (2013). Code for Design of Strengthening Concrete Structure (English Version).

[140] (2021). Standard Test Method for Evaluation of Bond Properties of FRP Composite Applied to Concrete Substrate using Single-Lap Shear Test.

[141] Moshiri N., Tajmir-Riahi A., Mostofinejad D., Czaderski C., Motavalli M. (2019). Experimental and analytical study on CFRP strips-to-concrete bonded joints using EBROG method. Compos. Part B Eng..

[142] Bilotta A., Ceroni F., Di Ludovico M., Nigro E., Pecce M., Manfredi G. (2011). Bond efficiency of EBR and NSM FRP systems for strengthening concrete members. J. Compos. Constr..

[143] Ueno S., Toutanji H., Vuddandam R. (2015). Introduction of a stress state criterion to predict bond strength between FRP and concrete substrate. J. Compos. Constr..

[144] Toutanji H., Saxena P., Zhao L., Ooi T. (2007). Prediction of interfacial bond failure of FRP–concrete surface. J. Compos. Constr..

[145] Ko H., Matthys S., Palmieri A., Sato Y. (2014). Development of a simplified bond stress–slip model for bonded FRP–concrete interfaces. Constr. Build. Mater..

[146] Pellegrino C., Tinazzi D., Modena C. (2008). Experimental study on bond behavior between concrete and FRP reinforcement. J. Compos. Constr..

[147] Adhikary B.B., Mutsuyoshi H. (2001). Study on the bond between concrete and externally bonded CFRP sheet. FRPRCS-5: Fibre-Reinforced Plastics for Reinforced Concrete Structures Volume 1, Proceedings of the Fifth International Conference on Fibre-Reinforced Plastics for Reinforced Concrete Structures, Cambridge, UK, 16–18 July 2001.

[148] Czaderski C., Olia S. EN-CORE round robin testing program-contribution of EMPA. Proceedings of the 6th International Conference on FRP Composites in Civil Engineering.

[149] Ko H., Sato Y. (2007). Bond stress–slip relationship between FRP sheet and concrete under cyclic load. J. Compos. Constr..

[150] Toutanji H., Han M., Ghorbel E. (2012). Interfacial Bond Strength Characteristics of FRP and RC Substrate. J. Compos. Constr..

[151] Santandrea M., Imohamed I.A.O., Carloni C. (2020). Width effect in FRP–concrete debonding mechanism: A new formula. J. Compos. Constr..

[152] Täljsten B. (1994). Plate Bonding: Strengthening of Existing Concrete Structures with Epoxy Bonded Plates of Steel or Fibre Reinforced Plastics. Ph.D. Thesis.

[153] Zhang J. (2026). Large-Scale Externally Bonded Fibre-Reinforced Polymer Systems for Tension Strengthening of Existing Reinforced Concrete Structures with and without Fibre Anchors. Ph.D. Thesis.

[154] Li W., Huang P., Chen Z., Cui H., Guo X., Wu B. (2022). Bond–slip model considering the interface shear stress reversal phenomenon and data dispersion for FRP–concrete interface. Eng. Fract. Mech..

[155] Li P.-D., Zhao Y., Tao Z., Jiang C. (2024). Nonuniformity in stress transfer across FRP width of FRP-concrete interface. Eng. Struct..

[156] Li Z., Zhang J., Pan C., del Rey Castillo E. Three-dimensional Numerical Simulation of FRP-to-Concrete Interface Debonding Considering Width Effect. Proceedings of the 17th International Symposium on Fiber-Reinforced Polymer (FRP) Reinforcement for Concrete Structures.

[157] Triantafillou T., Matthys S., Audenaert K., Balázs G., Blaschko M., Blontrock H., Czaderski C., David E., Di Tomasso A., Duckett W. (2001). Externally Bonded FRP Reinforcement for RC Structures.

[158] Monti G., Renzelli M., Luciani P. (2003). FRP adhesion in uncracked and cracked concrete zones. Fibre-Reinforced Polymer Reinforcement for Concrete Structures.

[159] Dai J.-G., Ueda T. (2003). A nonlinear bond stress-slip relationship for FRP sheet-concrete interface. Proceedings of the International Symposium on Latest Achievement of Technology and Research on Retrofitting Concrete Structures.

[160] Neubauer U., Rostasy F. (1999). Bond failure of concrete fiber reinforced polymer plates at inclined cracks—Experiments and fracture mechanics model. Spec. Publ..

[161] Chen J.F., Teng J. (2001). Anchorage strength models for FRP and steel plates bonded to concrete. J. Struct. Eng..

[162] Dai J.-G., Ueda T. (2003). Local bond stress slip relations for FRP sheets-concrete interfaces. Fibre-Reinforced Polymer Reinforcement for Concrete Structures.

[163] Ueda T., Dai J. New shear bond model for FRP-concrete interface-from modeling to application. Proceedings of the Second International Conference on FRP Composites in Civil Engineering-CICE.

[164] Zhou Y.-W., Wu Y.-F., Yun Y. (2010). Analytical modeling of the bond–slip relationship at FRP-concrete interfaces for adhesively-bonded joints. Compos. Part B Eng..

[165] Baky H.A., Ebead U., Neale K. (2012). Nonlinear micromechanics-based bond–slip model for FRP/concrete interfaces. Eng. Struct..

[166] Pan J., Wu Y.-F. (2014). Analytical modeling of bond behavior between FRP plate and concrete. Compos. Part B Eng..

[167] Savoia M., Ferracuti B., Mazzotti C. (2003). Non linear bond-slip law for FRP-concrete interface. Fibre-Reinforced Polymer Reinforcement for Concrete Structures.

[168] Sun W., Peng X., Yu Y. (2017). Development of a simplified bond model used for simulating FRP strips bonded to concrete. Compos. Struct..

[169] Lei M., Wang X., Chen J., Huang H., Lin J., Yan Z., Wu Z. (2024). Bond behavior of the FRP grid-concrete interface with geopolymer mortar as an adhesive. J. Build. Eng..

[170] Niemitz C.W., James R., Breña S.F. (2010). Experimental behavior of carbon fiber-reinforced polymer (CFRP) sheets attached to concrete surfaces using CFRP anchors. J. Compos. Constr..

[171] Alam M., Hussein A. (2013). Size effect on shear strength of FRP reinforced concrete beams without stirrups. J. Compos. Constr..

[172] Nanni A., Miller B., De Lorenzis L. (2001). Bond of fiber-reinforced polymer laminates to concrete. ACI Mater. J..

[173] Zhang H., Smith S.T. (2017). Influence of plate length and anchor position on FRP-to-concrete joints anchored with FRP anchors. Compos. Struct..

[174] Kanitkar R., Smith S.T., Lewis C. (2016). An Experimental Investigation on the Splay Portion of Embedded FRP Tension Anchors.

[175] del Rey Castillo E., Kanitkar R., Smith S., Griffith M., Ingham J. Fan-to-sheet failure mode of FRP anchors. Proceedings of the 14th international Symposium on Fiber-Reinforced for Concrete Structure.

[176] Singh A., del Rey Castillo E., Ingham J. (2019). FRP-to-FRP bond characterization and force-based bond length model. Compos. Struct..

[177] Sun W., Liu H., Wang Y., He T. (2018). Impacts of configurations on the strength of FRP anchors. Compos. Struct..

[178] Villanueva Llauradó P., Gómez J.F., González Ramos F.J. (2018). Influence of the anchor fan position on the performance of FRP anchors. High Tech Concrete: Where Technology and Engineering Meet, Proceedings of the 2017 fib Symposium, Maastricht, The Netherlands, 12–14 June 2017.

[179] del Rey Castillo E., Kanitkar R., Smith S.T., Griffith M.C., Ingham J.M. (2019). Design approach for FRP spike anchors in FRP-strengthened RC structures. Compos. Struct..

[180] Flores I.A.C., Gómez J.F., Llauradó P.V., Ferreira A. (2020). An empirical model to estimate FRP anchored joint strength using spike anchors. Compos. Struct..

[181] Fuchs W., Eligehausen R., Breen J.E. (1995). Concrete capacity design (CCD) approach for fastening to concrete. Struct. J..

[182] Eligehausen R., Cook R.A. (2006). Behavior and design of adhesive bonded anchors. ACI Struct. J..

[183] (1997). Code Requirements for Nuclear Safety Related Structures.

[184] Zhang W., Yan J.-B. (2017). Bond Properties of Additional Anchorage Schemes for CFRP Plate-Concrete Externally Bonded System. ACI Struct. J..

[185] Ceroni F., Pecce M. (2010). Evaluation of bond strength in concrete elements externally reinforced with CFRP sheets and anchoring devices. J. Compos. Constr..

[186] Camli U.S., Binici B. (2007). Strength of carbon fiber reinforced polymers bonded to concrete and masonry. Constr. Build. Mater..

[187] del Rey Castillo E., Dizhur D., Griffith M., Ingham J. (2018). Strengthening RC structures using FRP spike anchors in combination with EBR systems. Compos. Struct..

[188] Cook R.A., Kunz J., Fuchs W., Konz R.C. (1998). Behavior and design of single adhesive anchors under tensile load in uncracked concrete. Struct. J..

[189] Boumakis I., Ninčević K., Marcon M., Vorel J., Wan-Wendner R. (2022). Bond stress distribution in adhesive anchor systems: Interplay of concrete and mortar creep. Eng. Struct..

[190] Guadagnuolo M., Faella G., Frunzio G., Massaro L., Brigante D. (2023). The capacity of GFRP anchors in concrete and masonry structures. Procedia Struct. Integr..

[191] Zhang J., del Rey Castillo E., Kanitkar R., Lin S.-H., Allen T., Hogan L., Borwankar A.D. (2026). Unified Strength Model for FRP Anchors Under Pullout and Concrete-Related Failure Modes. J. Compos. Constr..

[192] Kalfat R., Al-Mahaidi R. (2016). Mitigation of premature failure of FRP bonded to concrete using mechanical substrate strengthening and FRP spike anchors. Compos. Part B Eng..

[193] Ozbakkaloglu T., Fang C., Gholampour A. (2017). Influence of FRP anchor configuration on the behavior of FRP plates externally bonded on concrete members. Eng. Struct..

[194] Alam M.A., Alshaikhly A.S., Mustapha K.N. (2016). An experimental study on the debonding of steel and cfrp strips externally bonded to concrete in the presence of embedded shear connectors. Arab. J. Sci. Eng..

[195] Mostafa A.A., Razaqpur A.G. (2013). A new CFRP anchor for preventing separation of externally bonded laminates from concrete. J. Reinf. Plast. Compos..

[196] Zhang J., del Rey Castillo E., Allen T., Hogan L., Kanitkar R., Borwankar A. (2026). Behavior and Predictive Model of Externally Bonded FRP Strips Anchored to Reinforced Concrete. J. Compos. Constr..

[197] Brena S.F., McGuirk G.N. (2013). Advances on the behavior characterization of FRP-anchored carbon fiber-reinforced polymer (CFRP) sheets used to strengthen concrete elements. Int. J. Concr. Struct. Mater..

[198] CDR (2025). Guidelines for the Design, Execution, and Inspection of Structural 385 Strengthening Interventions Using Fiber-Reinforced Composites.

[199] del Rey Castillo E., Griffith M., Ingham J. (2019). Straight FRP anchors exhibiting fiber rupture failure mode. Compos. Struct..

[200] Zhang J., del Rey Castillo E., Borwankar A. (2026). Experimental Database of 650 Straight and Bent Fiber Anchor Single-Lap Shear Tests Covering Anchor Rupture, Concrete Cone, Combined Cone, and Bond Failures.

[201] Smith S.T., Kim S. (2008). Shear strength and behaviour of FRP spike anchors in FRP-to-concrete joint assemblies. Proceedings of the 5th International Conference on Advanced Composite Materials in Bridges and Structures 2008 (ACMBS-V 2008).

[202] Llauradó P.V., Fernández-Gómez J., González Ramos F.J. (2017). Influence of geometrical and installation parameters on performance of CFRP anchors. Compos. Struct..

[203] Muciaccia G., Khorasani M., Mostofinejad D. (2022). Effect of confinement on the behavior of FRP straight anchors. Bond in Concrete-Bond, Anchorage, Detailing, Proceedings of the 5th International Conference, Stuttgart, Germany, 25–27 July 2022: Proceedings.

[204] Kanitkar R., del Rey Castillo E., Zhang J., Smith S. An Updated Design Approach for Fiber Anchors for Strengthening of RC Structures with FRP Systems. Proceedings of the 17th International Symposium on Fiber-Reinforced Polymer (FRP) Reinforcement for Concrete Structures (FRPRCS17).

[205] Godat A., Ceroni F., Chaallal O., Pecce M. (2017). Evaluation of FRP-to-concrete anchored joints designed for FRP shear-strengthened RC T-beams. Compos. Struct..

[206] Dong K., Gao Y., Yang S., Yang Z., Jiang J. (2023). Experimental investigation and analytical prediction on bond behaviour of CFRP-to-concrete interface with FRP anchors. Case Stud. Constr. Mater..

[207] American Concrete Institute (2023). Design and Construction of Externally Bonded Fiber-Reinforced Polymer (FRP) Systems for Strengthening Concrete Structures—Guide.

